# Organisational-level risk and health-promoting factors within the healthcare sector—a systematic search and review

**DOI:** 10.3389/fmed.2024.1509023

**Published:** 2025-01-17

**Authors:** Magnus Akerstrom, Jens Wahlström, Agneta Lindegård, Inger Arvidsson, Anna-Carin Fagerlind Ståhl

**Affiliations:** ^1^Region Västra Götaland, Institute of Stress Medicine, Gothenburg, Sweden; ^2^School of Public Health and Community Medicine, Institute of Medicine, The Sahlgrenska Academy at University of Gothenburg, Gothenburg, Sweden; ^3^Department of Public Health and Clinical Medicine, Sustainable Health, Umeå University, Umeå, Sweden; ^4^Division of Occupational and Environmental Medicine, Lund University, Lund, Sweden

**Keywords:** risk factors, health-promoting factors, healthcare, organisational-level, occupational health and safety management, prevention

## Abstract

**Introduction:**

The healthcare sector is globally experiencing increasing demands and workplace interventions on an organisational level is sought to create healthy workplaces. The aim of this study was to provide an overview of Nordic research on the work environment and health of healthcare professionals, with a focus on identifying organisational-level risk and health-promoting factors.

**Methods:**

This systematic search and review was based on an analysis of studies published in peer-reviewed journals between 1 January 2016 and 3 January 2023. The selected studies investigate the relationships between organisational-level risk and health-promoting factors and measures of health and well-being among healthcare professionals during ordinary operations. To increase applicability, this systematic search and review was limited to the Nordic countries as they share the same context with a publicly-funded widely accessible healthcare system. A total of 2,677 articles were initially identified, with 95 original studies meeting the criteria for relevance and quality.

**Results:**

Identified organisational risk and health-promoting factors were categorised into five categories: work schedule distribution, operations design and work methods, ergonomic conditions, working conditions and personnel policies, and the organisation’s ethical environment. In addition, two themes across the categories emerged, providing further insight into the implications for practice. The first theme emphasises risk and health-promoting factors in the actions that employers take to fulfil the organisation’s goals. The second theme emphasises risk and health-promoting factors in connection with the ability of employees to do their jobs at a level of quality they deem acceptable.

**Conclusion:**

Several organisational-level risk and health-promoting factors were identified, and the results indicate that the actions that employers take to fulfil the health-care organisation’s goals and promote the ability of employees to provide high-quality care are important for the health and wellbeing of healthcare employees.

## 1 Introduction

High-quality healthcare is essential for social welfare, and attention to the health and wellbeing of healthcare workers is a crucial aspect of this effort. Reduced health in healthcare workers can have adverse effects on the individual healthcare workers. In addition, it may also lead to reduced quality in patient care, the risk of accidents, and challenges attracting and retaining a skilled workforce. Maintaining healthcare workers´ health is especially important since the competition for healthcare professionals is increasing due to an aging population in many societies (1–6). Despite this, healthcare in Europe is recognised as a high-risk sector from an employee wellbeing perspective (7), and healthcare workers report the highest levels of work-related stress compared to other professionals (8). They also experience poor wellbeing (9, 10) and physical symptoms (11).

Challenges within the healthcare sector arise from demands connected to healthcare work, which include contact with distressed and ill patients, work overload, up-to-date learning, and high-quality standards of performance (8). In addition, ongoing medical developments have resulted in growing demands for speed, complexity, and responsibility; an increased administrative burden, and reduced autonomy among healthcare workers (12, 13). In order to maintain healthy workplaces, job demands need to be manageable, and workers need to have access to sufficient resources to balance these demands (14). This challenging situation is not unique to Europe, and the World Health Organization (WHO) estimates that within the healthcare sector alone, there will be a shortfall of 10 million employees globally by 2030 (15).

Creating healthy workplaces requires organisational approaches that aim to improve working conditions and the organisation of work, rather than individual approaches that aim to improve workers’ competencies, knowledge, and coping capacity (16–18). In addition, instead of simply preventing harm, an approach that focuses on promoting employee wellbeing has been recommended as a way to improve working conditions within the healthcare sector (19). Such organisational-level interventions require not just in-depth knowledge of the healthcare sector (i.e., challenges, structure, and processes imbedded in that system and culture) but also knowledge on risk and health-promoting factors (i.e., working conditions that increase the likelihood of illness among employees or reduce the likelihood of health, and increase the likelihood of health among employees or reduce the risk of illness, respectively) that may be targeted (20, 21).

At present, the knowledge of the impact of risk and health-promoting factors on the workplace level within the healthcare sector is extensive, as a wide range of systematic reviews have been performed. These systematic reviews have provided evidence of the associations between burnout and a high workload, time constraints, value incongruence, low level of control, insufficient support from colleagues and managers, lack of collaboration, inadequate rewards, insufficient staffing, shifts exceeding 12 h, limited scheduling flexibility and uncertain employment conditions (22–25); musculoskeletal disorders and pain due to awkward working postures, a large number of patients, administrative work, vibration, and repetitive work (26, 27); and job satisfaction with workload and income, responsibility, recognition, autonomy and collaboration (28, 29).

However, there is still limited knowledge of the underlying causes of the presence or absence of these risk and health-promoting factors (i.e., risk and health-promoting factors on a higher organisational level). Following the principles of the hierarchy of controls for occupational safety and health (30, 31), risks to health and wellbeing should be reduced or eliminated by targeting the organisational level rather than the workplace or individual level. Thus, there is an urgent need to increase knowledge on organisational-level risk and health-promoting factors that may be used to improve the health of employees within the healthcare sector. To increase applicability to practice, this systematic review was limited to the Nordic context (Denmark, Finland, Iceland, Norway and Sweden), where all countries have a publicly-funded, widely accessible healthcare system (32).

The aim of this study was to provide an overview of Nordic research on the work environment and health of healthcare professionals, with a focus on identifying organisational-level risk and health-promoting factors.

## 2 Methods

### 2.1 Study design

Due to the multifaceted nature of organisational-level risk and health-promotive factors, in combination with the absence of earlier systematic reviews that could be used to guide the search, a broad scope that incorporates multiple study types rather than focusing on a single preferred study design had to be used. Thus, this study was carried out as a systematic search and review with a narrative summary (33) and reported according to the Preferred Reporting Items for Systematic reviews and Meta-Analyses (PRISMA) 2020 statement (34); see supporting information on-line Supplementary material (Prisma 2020 checklist). This study has not been reviewed by the Swedish Ethical Review Authority. This is not required for this type of study according to the Swedish Ethical Review Act. Informed consent to participate was not applicable in this study. No protocol exists for this review, since it was first commissioned as a part of a government assignment to the Swedish Agency for Work Environment Expertise and the absence of ethical review requirements.

### 2.2 Inclusion and exclusion criteria

Studies investigating health-related risk and health-promoting factors for healthcare professionals in the Nordic countries that were published in peer-reviewed journals between 1 January 2016 and 3 January 2023 were included. The start date of the searches was a pragmatic choice used to increase the relevance of the included studies by reflecting the current context and normal operations. The search strategy was structured according to SPIDER (sample, phenomenon of interest, design, evaluation, and type of research) as we expected a wide range of study designs, including quantitative, qualitative, and mixed method designs (35). Studies were included if they examined the relationship between health and illness in relation to risk and health-promoting factors at the organisational level, or employees’ experiences of these factors. Descriptive studies that described relationships without examine the relationship between health and illness in relation to risk and health-promoting factors at the organisational level were excluded. Outcomes that cannot be directly seen as an aspect of health or illness have also been excluded, although they may be an outcome of a risk or health-promoting factor and related to health or illness. For example, various performance-related outcomes, such as patient satisfaction, quality of care, or incidents, have been excluded since such outcomes do not directly reflect worker health. Outcomes related to employee turnover, such as the desire to leave or remain in the workplace or organisation, have also been excluded. Finally, studies conducted under non-ordinary or non-generalisable conditions, such as pandemics or crises, have been excluded. Complete inclusion and exclusion criteria can be found in Table 1.

**Table 1:** Inclusion and exclusion criteria structured according to SPIDER (sample, phenomenon of interest, design, evaluation, and type of research).

SPIDER	Inclusion criteria	Exclusion criteria
Sample	Healthcare professionals in the Nordic countries	Healthcare professionals outside the Nordic countries
Phenomenon of interest	Organisational and health-related risk and health-promoting factors that can affect employees’ health and wellbeing, either directly or by affecting job demands and resources at the workplace level	Risk and health-promoting factors at the workplace level. Organisational risk and health-promoting factors that affect staff turnover, as well as willingness to remain at the workplace, or performance-related outcomes, such as patient satisfaction, quality of care, or patient-related incidents
Design	Observational studies under ordinary conditions	Studies under non-generalisable or extraordinary conditions, for example, purely experimental studies, intervention studies, and studies conducted during pandemics and crises
Evaluation	Studies that examined the relationship between health and illness in relation to risk and health-promoting factors at the organisational level	Studies that did not examine the relationship between health and illness in relation to risk and health-promoting factors at the organisational level
Research type	Quantitative, qualitative, and mixed methods studies	Systematic reviews, intervention studies, experimental studies, and grey literature

### 2.3 Information sources and search strategy

Literature searches were developed and conducted by librarians/information specialists to reflect the concept outlined by the project team. A set of key articles were identified before the search process, which were used to generate search terms (MeSH and free-text terms) and test the effectiveness of the strategies in each database. A combination of three different thematic search terms (blocks) were used: (1) population (e.g., “healthcare worker*,” “healthcare personnel*,” “health professional*,” etc.), (2) the phenomenon of interest (e.g., “occupational health*,” “workplace health*,” “employee health*,” etc.), and (3) the context (e.g., “Sweden,” “Norway,” Denmark,” “Finland,”” Iceland.”). The complete list of search terms is presented in the on-line Supplementary material (Search terms). To cover a wide range of disciplines such as healthcare, psychology, and occupational health research, the search was performed using four different databases (Pubmed, Scopus, Cindahl, and PsycINFO) 3 January 2023.

### 2.4 Selection process

Records found during the search phase were exported to a reference management software (EndNote) to identify and remove duplicates. To ensure adequate understanding and consistency in the application of inclusion and exclusion criteria, a calibration exercise was carried out within the project team prior to the formal screening. In this calibration exercise, the project team met to discuss inclusion and exclusion decisions on randomly selected records until adequate consensus and consistency was assessed to be reached within the group. The records were then screened based on the inclusion and exclusion criteria using Covidence, a web-based application for systematic reviews. The initial screening on the title/abstract level and the full-text assessments of each record were done independently by two of the authors (MA, JW, ALA, and/or ACFS). Cohen’s Kappa showed an agreement between 0.33 and 0.60 between the different evaluators. To increase the agreement between evaluators, the project team met up to discuss inclusion and exclusion decisions until adequate consensus and consistency was assessed to be reached. Disagreements were resolved by discussion until consensus was reached.

### 2.5 Quality assessment

The methodological quality of each included study was assessed by two of the authors (MA and JW). To provide a nuanced view of study quality across multiple research designs, in line with the methodology of this systematic search and review, the 2018 Mixed Methods Appraisal Tool (MMAT) was used. MMAT is designed to review the quality of studies with different designs and varying methods (36). When using the MMAT, no scores or overall assessments, such as low/medium/high quality, are calculated; rather, the MMAT provides an in-depth picture of the quality of the studies. The quality review was conducted in two stages. In the first stage, each study was evaluated based on two screening questions (whether there were clear research questions and whether these questions could be investigated using the available data in the study). The assessment of these questions (yes, no, or cannot tell) determined whether the study should be included or excluded due to a lack of methodological quality. In the second stage, the included studies were evaluated using five additional and specific study design questions (with response alternatives: yes, no, or cannot tell), to provide an in-depth picture of the quality of the study. In this stage, the templates for qualitative studies, randomised controlled trials, non-randomised trials, and mixed methods studies were used. Any disagreement during the quality appraisal were resolved by discussion until consensus was reached.

### 2.6 Data extraction

Data extraction was performed by the authors (MA, JW, ALA, and ACFS) and cross-checked to ensure accuracy and consistency in the extracted data by another author (MA or ACFS). To include both information on the identified organisational-level risk or health-promoting factors and contextual and methodological information that may be used to support the analysis, a matrix, including: (A) country representing the context of the study, (B) study aim, (C) study design, (D) study period, (E) study population, (F) Size of the study population (G) outcomes, and (H) identified organisational-level risk or health-promoting factors was used and is provided in the on-line Supplementary material (Data extraction matrix). Any disagreements during data extraction were resolved by discussion until consensus was reached.

### 2.7 Data synthesis

Since a large heterogenicity was expected in the included studies, the data synthesis was conducted with a narrative summary based on which aspects of the organisation of health care had been examined. This categorisation was done jointly by the authors (MA, JW, ALA, IA, ACFS) and was reported together with the more descriptive compilation of the included studies. Finally, overarching themes were also identified across these categories (i.e., meaningful patterns that contribute to a better understanding) (37). An example of the qualitative data synthesis process, including the identification of the categorisation and identification of overarching themes can be seen in Table 2.

**Table 2:** Example of the qualitative data synthesis process.

Theme	Sub-theme	Category	Sub-category	Organisational factor	Quote
Organisational-level risk and health-promoting factors within healthcare	Importance of the organisation’s culture and values and what it communicates to its employees	Operations design and work methods	Risk factor	Operations design; working alone	“There was a significant association between working alone and psychological distress, both in univariate and multivariate models corrected for age and gender”

## 3 Results

### 3.1 Study selection

**Figure 1:**
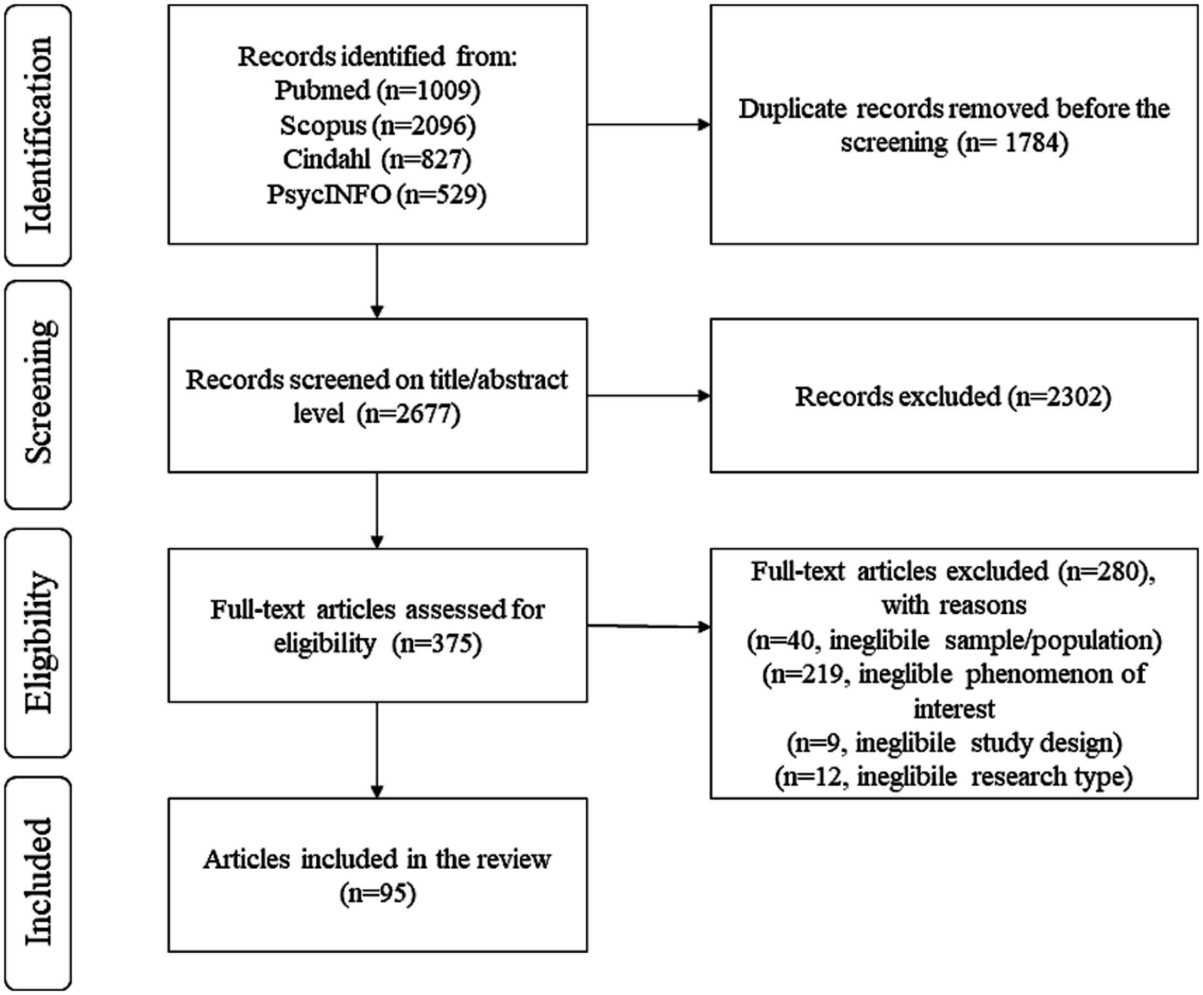
PRISMA 2020 flow diagram of review process.

The database searches identified 4,461 records, and 2,677 record were screened on the title/abstract level after removal of duplicates. Of these, 375 full-text articles were reviewed, and 95 were included for analysis (Supplementary Table S1). A flow diagram of the review process is shown in Figure 1. A list of excluded full-text articles are provided in the on-line Supplementary material (Excluded full-text articles).

### 3.2 Characteristics of the included studies

Most of the included studies had a cross-sectional design (*n* = 34, 36%) or a longitudinal cohort design (*n* = 34, 36%). A total of 20 studies (21%) were qualitative, and two studies (2%) used a mixed methods design. The remaining five studies (5%) used a case–control design or examined the importance of organisational risk and health-promoting factors in connection with natural experiments under normal operations (quasi-experimental intervention or randomised field experiments) (Supplementary Table S1).

Two out of five studies (*n* = 38, 40%) included healthcare professionals from different professional groups, without examining the groups separately or specifying the groups in greater detail. The other studies (*n* = 57, 60%) focused on specific professional groups, including registered nurses (*n* = 37, 39%), physicians (*n* = 9, 9%), healthcare managers (*n* = 5, 5%), midwives (*n* = 4, 4%), dental hygienists and other dental professionals (*n* = 2, 2%), and psychotherapists (*n* = 1, 1%) (Supplementary Table S1).

There was an even distribution of studies from the Nordic countries, with 27 studies (28%) from Finland, 25 (26%) from Sweden, 22 (23%) from Norway and 22 (23%) from Denmark. Iceland was the exception with only one study (1%) (Supplementary Table S1). No overall differences in the identified factors could be seen between the Nordic countries.

A majority of the included studies studied organisational risk factors (*n* = 67, 71%) connected to a wide range of outcomes with a focus on both mental and physical illness and disease (Supplementary Table S1). The remaining studies (*n* = 28, 29%) focused on health-promoting factors connected to outcomes such as job satisfaction, motivation, engagement, etc. (Supplementary Table S1).

### 3.3 Quality assessment results

All reviewed studies were assessed to be of sufficient methodological quality and were included in the systematic review. In the in-depth assessments of the methodological quality of the included studies, most of the qualitative and mixed method studies were found to lack information on whether the authors had quality assured the article using a checklist for reporting the study (such as the “Consolidated criteria for reporting qualitative research” [COREQ] or similar resource). For the quantitative studies (17 out of 75 studies), questions were raised regarding whether the participants were representative of the intended study population due to low response rates and/or a non-random sample of the study population. The checklist for the evaluation of methodological quality is provided in the on-line Supplementary material (Quality assessment).

### 3.4 Organisational-level risk and health-promoting factors

To obtain an overview of the results, all studies were categorised based on which aspects of the organisation of health care had been examined (Supplementary Table S1). Results identified risk and health-promoting factors in the organisation of health care in terms of the distribution of working time schedules (*n* = 39, 36%), design of operations and working methods (*n* = 28, 26%), ergonomic conditions (*n* = 18, 17%), terms of employment and personnel policy (*n* = 13, 12%), and the organisation’s ethical environment (*n* = 10, 9%), Table 3. These categories are described in detail below. In some cases, a single study contained risk and health-promoting factors associated with more than one of these categories, bringing the total number of studies in the above summary to more than 95.

**Table 3:** Summary of the five identified categories of organisational-level risk-and health-promoting factors, and the types of factors associated with each of these categories.

Identified categories	Work schedule distribution	Operations design and work methods	Ergonomic conditions	Terms of employment and personnel policies	The organisation’s ethical environment
Included studies (*n*)^a^	39	28	18	13	10
Types of organisational-level risk-or health-promoting factors	Shift type and distribution of shifts in the short term and over time Number of working hours per shift and week Distribution of shifts and time off/rest	Organisation of the work, alone and with others Objectives and quality management Models/ principles guiding the work	Assistive devices and work tools (physical and cognitive) Physical work environment	Permanent/ temporary employment Salary and other rewards Opportunities to adapt the work Tutorials, courses, training	Communicated policies and guidelines Support for employees in ethical issues Opportunities to perform work in line with ethical values

^aOne study can contribute to more than one category bringing the total number of studies to more than 95.^

#### 3.4.1 Work schedule distribution

A relatively large group of studies (*n* = 39) investigated employees health related to the distribution of working time schedules, that is, how shifts and working hours were distributed among existing staff. Shift work can refer to both fixed night shifts and rotating shifts. Shifts can rotate regularly or irregularly, and their duration may vary. For many healthcare organisations that must be staffed around the clock, the need for evening and night work is unavoidable. Still, the distribution of these shifts and working hours among available staff is a changeable factor at an organisational level.

Schedules that largely included night shifts and shift work, especially over several years, were found to be an organisational-level risk factor. An association was found between night work and cerebrovascular disease and stroke (38), sleep disturbances and severe fatigue (39), exhaustion (40), heart disease (41), diabetes (42), sick leave (43) and work-related accidents (44). Working several night shifts in a row increased the risk of exhaustion (45), sick leave (46, 47), and premature birth (48). Working night shifts for more than six years increased the risk of dementia (49, 50), and multiple night shifts over more than five years increased the risk of telomere shortening, which, in turn, increases the risk of breast cancer (51). However, the findings were not completely unanimous, and some studies found no association between night work and health (52, 53) or sick leave (44), or only for specific subgroups (54–56). Evening shifts were also found to be a risk factor and were associated with diabetes (42), long-term sick leave (46), and the incidence of accidents during these shifts (57, 58). Some studies found that both evening and night shifts increase the risk of both mental illness (50, 59) and mortality (60), while other studies conclude that the increased risk of mental illness was greater for people who work night shifts than evening shifts (61, 62). Shift and night work was also associated with sleep problems and insufficient recovery (63).

The distribution of working time schedules over the day and week was also found to be a risk factor. Long shifts of more than eight hours and long weeks of more than 40 h increased the risk of sick leave (46, 47, 64), work-related accidents (65), and work-related injuries (66). A schedule with fewer but longer shifts on weekends (12-h shifts instead of 8-h shifts) did not affect job satisfaction among registered nurses, but the effect of this schedule on health depended on the nurses’ general health and family situation (67). The number of 24-h on-call shifts was positively associated with burnout among surgeons (68).

Another identified risk factor was quick returns (i.e., a short duration between shifts), which was associated with perceived stress (69, 70), sleep disturbances and severe fatigue (39, 139), exhaustion (45), heart disease (41), cerebrovascular disease and stroke (38), sick leave (46, 64, 70), premature birth (48), and work-related accidents (43, 44, 58, 65).

The importance of these risk factors was also seen in studies that examined the impact of reducing shift work, quick returns, and working hours. Night workers’ symptoms of mental illness improved when they stopped working night shifts (71), and their sleep disturbances and severe fatigue decreased when they reduced the number of quick returns, discontinued night work, or reduced the number of night shifts (39). A reduction in the number of quick returns also reduced the risk of work-related injuries among registered nurses (72). When working hours were reduced from eight to six hours (with the same salary), assistant nurses and registered nurses felt that they had more energy, both on the job and outside of work (72, 137).

A health-promoting factor was identified in the ability to influence the schedules, working hours, and holidays, which was perceived as a reward (73) and increased job satisfaction (74) among registered nurses. Another study found that participation in the planning of working hours resulted in increased control, but not increased well-being, compared to traditional planning among healthcare workers (138).

#### 3.4.2 Operations design and work methods

The second largest category (*n* = 28) comprises studies focusing on how the organisation and its work are designed and what working methods prevail and are rewarded within the organisation. This includes different ways in which tasks and responsibilities have been distributed and how the organisation has chosen to structure the work, measure quality, provide feedback, offer rewards, and manage goals. Although all studies in this category address the design of operations and working methods, this is a broad field, and the identified articles mostly examine different aspects, in different contexts, for different groups, making it difficult to draw overall conclusions.

Some studies identified risk and health-promoting factors in the social aspect of how work was designed in terms of collaborations and hierarchies. Associations were identified between working alone and increased perceived anxiety (75) and musculoskeletal disorders and pain (76), but also with increased job satisfaction (77). Non-hierarchical collaboration was associated with motivation among primary care staff (78), and collaboration within the organisation and with policymakers and support (administratively and organisationally) increased well-being among healthcare managers (79). Working in self-organising teams (80) and having the ability to self-manage (81) were positively associated with job satisfaction.

Other studies identified both risk and health-promoting factors in how the tasks were designed and distributed. Physicians who were required to perform illegitimate tasks had increased presenteeism (82). The risk of exhaustion increased for physicians within primary care when they were forced to take over tasks from specialist care providers and when documentation and administration increased and became more complex (83). For general practitioners in Norway, the number of consultations per day had no association with stress, but the number of consultations containing elements of conflict did (84). Among registered nurses, the manner in which responsibility for patients was distributed was both positively and negatively associated with various aspects of stress (85). Among healthcare workers in Denmark, job satisfaction increased when clinical tasks were delegated from the physician to other health professions (86). The dissatisfaction of general practitioners with their work situation decreased when the time per patient consultation increased from less than 10 min to more than 20 min (77). When the work of midwives was organised to ensure that patients could have one midwife throughout their pregnancy, this resulted in lower rates of burnout (87) and increased job satisfaction, as the midwives felt important and appreciated (88). The more primary care units relied on a lean-based working method, the lower the levels of fatigue among staff, who also reported a greater sense of well-being (89). Operational designs that resulted in short-term planning and uncertainty about the future and finances were a risk factor for poor health among healthcare managers (79). Work that involves standing still was not associated with pain among healthcare workers (90).

Risk and health-promoting factors were also identified in the way organisations set up and managed their goals and how they chose to measure quality and performance. Clear goals and systematic quality work were associated with increased motivation among primary care staff (78), while the use of inadequate quality measures was associated with reduced job satisfaction among registered nurses (91). A focus on cost-effectiveness within an organisation sparks frustration among home care staff, who feel that they are not able to work as effectively as they would like (92). Accreditation of the enterprise was negatively associated with physicians’ job satisfaction if it was perceived as a means of control, but increased job satisfaction if it was considered to improve quality (93). Healthcare workers who were exposed to demands connected to financial constraints, administration, and productivity by their senior management had increased sickness absence compared to other healthcare workers (94). When registered nurses were rewarded based on performance goals, it sometimes resulted in increased stress (73). Registered nurses reported feeling more motivated and that collaboration improved when tasks were visually presented and could be discussed and ticked off after completion by using activity boards (95).

Finally, this category also included studies of risk and health-promoting factors linked to whether senior management had the necessary conditions to acknowledge and understand the needs of their employees. The perception that the management of a healthcare organisation focuses and acts based on the needs and desires of employees is positively linked to job satisfaction and engagement among registered nurses (96). Registered nurses reported that being seen and receiving recognition and feedback from senior management increased motivation (73, 74). A senior management team that supported patient safety and inter-unit teamwork was associated with lower levels of burnout among registered nurses (97). Registered nurses and midwives perceived that managers promoted health when they had the opportunity to take a hands-on approach, whereas a lack of instructions and procedures was perceived as a risk factor (98). Registered nurses experienced greater job satisfaction when their line manager had a moderate number of employees, thus enabling them to take on a more active leadership role (99). In another study, the number of subordinate registered nurses had no correlation with the neck and back pain of unit managers (100).

#### 3.4.3 Ergonomic conditions

In this category, 18 studies had investigated the results of actions at the organisational level to eliminate ergonomic risk factors and optimise ergonomic conditions, including electronic information and communication systems (e.g., electronic medical record systems or registers), with the purpose of simplify or facilitating work.

Risk and health-promoting factors were seen in measures that reduced strain in individual work tasks at the organisational level and measures that provided and enabled the use of various aids to reduce the workload and the risk of injury. When working in clients’ private homes, difficulty in using adequate aids or equipment poses a risk of injury (63). The inability to use assistive devices when moving patients from one place to another was associated with ill-health among healthcare workers (63, 101, 102). Access to adapted assistive devices in the form of prism glasses reduced the risk of neck pain and injury among dental professionals by limiting neck strain (103). The design of medical equipment did not affect pain among dialysis registered nurses (104), but a closer analysis identified risks associated with repetitive tasks and the design of the workplace and various tools (76).

Other risk and health-promoting factors were found in the physical work environment. A work environment that was perceived as pleasant and allowed for social interaction increased job satisfaction among healthcare workers in long-term dementia care homes (105). Access to daylight was perceived to be important for well-being and working ability (106). The inability to see the outside world (e.g., through a window) over the course of an entire shift, as well as the long-term use of surgical equipment that requires darkness, contributed to stress and exhaustion (106). Acceptable indoor air quality was identified as an important factor for decreasing hoarseness among healthcare workers (107, 108). However, the use of blue lights in healthcare facilities had no effect on either the mood or stress levels of healthcare workers compared to normal lighting (109).

Lastly, IT systems were perceived as a health-promoting factor, if they reduced documentation requirements, improved access to information, and gave staff a sense of security (110, 111). However, they were identified as a risk factor if their use was found to be an obstacle to the ability of staff to do their work or were fraught with technical problems (112–114). The perceived stress caused by electronic tools was reduced for registered nurses if they were perceived to be user-friendly (115). This was also the case for healthcare managers, if they had access to sufficient IT support (111). Daily use of multiple IT systems was associated with a higher level of stress compared to using only one system (115, 116). Physicians who already experienced time constraints reported more IT-related stress, and physicians in primary care experienced more stress related to IT than physicians in hospitals (113).

#### 3.4.4 Terms of employment and personnel policies

This category includes 13 somewhat diverse studies, which in various ways examined the conditions under which their organisation employs staff and how they take care of and support these staff.

Risk and health-promoting factors were identified in job security (73, 92), salary, and other monetary rewards (73, 79, 117), which were associated with higher levels of engagement and job satisfaction. Short-term work contracts, combined with shift work with variable shift lengths, irregular rest periods, and weekend shifts, increase the risk of sick leave (118). At the same time, a study found that temporarily employed registered nurses rated their health as better than permanent registered nurses (119). In the period preceding downsizing, staff absenteeism due to illness decreased, mainly among employees with temporary contracts (120). Requirements for employees to switch units against their will negatively affected job satisfaction (74).

Risk and health-promoting factors were also identified in the organisation’s systematic work to create conditions to increase the individual’s capacity to manage their own work and development. Two studies examine access to supervision among psychotherapists with different results: group clinical supervision was associated with lower stress (121), but no association could be confirmed between participation in clinical supervision and burnout (122). Another study investigated whether the opportunity to attend courses during working hours can be a health-promoting factor but found no correlation with job satisfaction (123). Physical exercise at work reduced pain and pain sensitivity more than exercise at home (124). Registered nurses reported that inadequate adaptation of work for pregnant workers and those with health problems was a reason for sickness absence (125).

#### 3.4.5 The organisation’s ethical environment

The category of studies that addressed the ethical environment of organisations includes relatively few studies (*n* = 10). However, they are generally homogeneous and examine the ability of employees to perform their work in accordance with their own fundamental values of what constitutes good care, as well as the values of their profession. The ethical environment also encompasses the extent to which the organisation encourages ethical discussions in the workplace and ensures that employees are supported in ethical issues and dilemmas.

Risk and health-promoting factors were identified in the ability of employees to act in accordance with their values and receive support in dealing with ethical issues, as this was seen as important for job satisfaction and engagement. When an organisation shares the values of its staff and ensures that there are resources and conditions for employees to be able to deal with ethical issues and act in accordance with their values, it is a health-promoting factor (126). Motivation and engagement increase when employees feel that they have adequate time (95) and sufficient staff (92, 98). Furthermore, when the number of employees in a unit increases or functions are outsourced, the risk of long-term sick leave decreases (127). Conversely, healthcare workers in home care services who are forced to “count the minutes” feel frustrated and unable to work as effectively (92), and inadequate staffing poses a health risk that entails extra stress, pressure, and responsibility (63).

For managers, the inability to implement decisions that were made higher up in the organisation or the obligation to implement decisions with which they personally disagree constitute a risk factor for future illness (128). Another risk factor for reduced job satisfaction arises when an individual’s values conflict with the values of the organisation. This demonstrates the importance of management understanding the ethical challenges related to the profession (91). Conversely, a health-promoting factor for job satisfaction was identified in the organisation’s encouragement of ethical discussions and support in grappling with ethical issues (129).

### 3.5 Overall themes in the categories

Although there was a wide variety within and between the categories in terms of the risk and health-promoting factors on which the studies focused, there were also similarities. Within each category, the studies demonstrated that what the organisation does to control and manage the work to meet its goals has an impact on employee health. This applies, for example, to how the organisation allocates working hours and staffs its operations, manages its objectives, and provides aids and support. In addition, there were studies in all categories that pointed to the importance of the organisation’s culture and values and what it communicates to its employees, including through priorities that affect both the work environment and the opportunities for employees to work effectively.

## 4 Discussion

This review presents knowledge from recent Nordic research on organisational risk and health-promoting factors in the healthcare sector. It takes a relatively new approach to risk and health-promoting factors, insofar as it focuses on the organisational level, that is, the structure, choice of principles, and values of the organisations. When work-related illness and well-being are discussed, workplace and individual factors are usually the dominant theme (18, 130, 131).

### 4.1 Organisational-level risk and health-promoting factors within healthcare

Our findings indicate that organisational-level risk and health-promoting factors can be divided into five categories: distribution of working time schedules; design of operations and working methods; ergonomic conditions; terms of employment and personnel policy; and the organisation’s ethical environment. In terms of their effect on the health of healthcare workers, these categories are well known. It is common knowledge that the scheduling of shifts (duration, number, and frequency, as well as time for recovery between shifts) can be both a risk factor for physical and mental illness, for example, exhaustion and cancer (22, 23, 132–134), and a health-promoting factor that increases job satisfaction (133). Furthermore, Aust et al. (16) recently showed the importance of job and task modification, flexible work and scheduling, and changes in the physical work environment in improving healthcare workers mental health and wellbeing. The HR practices of organisations have been associated with nurse absenteeism (135), and ethical value conflicts in healthcare have been associated with the health of nurses (2, 136). However, what this systematic review adds is a deeper understanding of how these risk and health-promoting factors can be created at the organisational level – and hence a better understanding of how to address risks at their roots and create a hotbed for a healthy and attractive work environment through the active engagement of the top management. It also identifies similarities between categories, highlighting the importance of including multiple perspectives within the employer’s occupational safety and health management, since both the actions that employers take to fulfil the healthcare organisation’s goals and the ability of employees to provide good quality care were found to be important for the health and wellbeing of healthcare employees. Consequently, employee health and wellbeing can and should be managed at the organisational level, not only at the workplace or individual level by first-line managers, according to the principles of the hierarchy of controls for occupational safety and health (30, 31) and recommendations from peers (16, 18).

This review also shows that there is a lack of knowledge on measures to promote well-being and health compared to knowledge of measures to counteract illness and disease. This is highlighted by the fact that most of the studies investigated risk factors rather than health-promoting factors. The definition of health-promoting factors differed substantially between the included studies, and in some studies, measures that sought to address risk factors rather than add something positive were also defined as health-promoting factors (e.g., reducing quick returns or alleviating strain/workload through well-adapted aids). Such factors may reduce the risk of illness but likely do not promote health or well-being, since they simply mean that a risk factor has been addressed. The utilisation of health-promoting factors within the occupational health and safety management of organisations may be particularly important if the healthcare sector (19) is to remain attractive to professions that are both demanding for the individual and essential for a well-functioning society.

### 4.2 Strengths and limitations

Some limitations need to be considered when interpreting the results of this review. The current study is limited to healthcare professions in the Nordic, countries and the findings cannot automatically be generalised to other professions or countries. Given the complex characteristics of healthcare organisations (20), future research would benefit from performing similar reviews for other contexts, rather than broadening the search criteria to include other professions and countries. Future research could also build on these findings to enable systematic reviews that include meta-analyses for individual categories of organisational-level risk-and health-promoting factors. The present review only considered studies published on or before 3 January 2023. We did not update the search further because we did not want to include studies that reflected the extraordinary circumstances following the COVID-19 pandemic, which currently influences research in this area. As a result, many studies published from 2021 an onwards were excluded during the study selection of this review.

A very broad approach was used to provide an overview of Nordic research on the work environment and health of healthcare professionals, with a focus on identifying organisational-level risk and health-promoting factors. This resulted in a wide range of study types across different healthcare professionals and settings, targeting a variety of outcomes. Although this heterogenicity did not allow for firm conclusions, it showed the large variety of studies within the area. Dividing these studies in five categories of organisational-level risk and health-promoting factors provided a better understanding of areas of interest when investigating potential risk factors within an organisation. However, due to this heterogenicity, it was not possible to perform a meta-analysis of the findings within each category.

It should also be pointed out that a large proportion of the included studies used a longitudinal cohort design. This means that they followed participants over time and investigated how factors related to health, illness, and the organisation of work changed in relation to each other. This is useful when pinpointing the risk and health-promoting factors in the work environment. A relatively large number of qualitative studies were identified. This helps to provide a deeper understanding of employees’ perceptions of risk and health-promoting factors. Two out of five studies included more than one occupational profession. This increases the generalisability of the results but at the expense of being able to comment on individual occupational groups. Many of the studies that reported results for individual occupational groups focused on registered nurses.

### 4.3 Implications for practice

This review offers valuable information on how leaders within healthcare organisations can promote employee wellbeing through strategies that target the way work is organised, designed, and managed. Our results call for action on the strategic level within the occupational health and safety management in healthcare organisations, since it is at the organisational level that opportunities arise to not only manage but eliminate risks in the work environment, and it is also here that there is an opportunity to promote health in the workplace (16, 18).

In addition, our analysis of overall themes can also give an indication of which perspectives on the organisation of work in health care are important for both employer and employees, regardless of which specific areas are considered. To ensure a sustainable, safe, and healthy working life, the effect of actions that employers take to meet the health care organisation’s goals and provide employees with the ability to provide high-quality care must be given equal priority. Management must ensure that its staffing, distribution of working time schedules, and choice of working methods are adequate to meet society’s needs for healthcare and that their operations are designed in a way that ensures the organisation can fulfil its mission. Yet it is equally important that management prevents employees from being exposed to the risk of illness and provides them with the opportunity to conduct their work in accordance with their fundamental values regarding what constitutes good care. The organisation must ensure that working methods, aids, and the physical work environment enable employees to perform their work in a manner that is satisfactory to both patients and employees. The terms of employment and work must be adapted to the organisation’s need for flexibility and simultaneously provide sufficient security to meet the employees’ needs for security and rewards. The organisation’s ethical environment must consider not only care priorities, but also how these affect employees.

## 5 Conclusion

Overall, the main contribution of this review is threefold. Firstly, our results indicate that organisational-level risk and health-promoting factors can be found within an organisations’ work schedule distribution, operations design and work methods, ergonomic conditions, terms of employment and personnel policies, and within the organisation’s ethical environment. Secondly, by addressing organisational-level factors within healthcare organisations, risks in the work environment may be eliminated rather than simply managed at the workplace level. Thus, our findings emphasise that the underlying causes of adverse working conditions within the healthcare sector must be identified and managed at the organisational and structural level. Lastly, two themes emerged across the categories, providing further insight into implications for practice. The first theme concerns how risk and health-promoting factors are present in the actions that are taken to fulfil the organisation’s goals, and the second theme concerns how these factors affect the ability of employees to perform their jobs at a level of quality that they consider reasonable. Thus, a successful approach to occupational health and safety management needs to consider both aspects when taking measures to improve working conditions, as well as the organisation of work within the healthcare sector. Integrating organisational-level factors in the occupational health and safety management could potentially result in a retention of skilled professionals within healthcare organisations’ both in the Nordic countries and globally.

## References

[1] Jun J Ojemeni MM Kalamani R Tong J Crecelius ML. Relationship between nurse burnout, patient and organizational outcomes: systematic review. Int J Nurs Stud. (2021) 119:103933. doi: 10.1016/j.ijnurstu.2021.103933, PMID: 33901940

[2] Larsman P Pousette A Skyvell Nilsson M Gadolin C Törner M. Ethical value conflicts in healthcare and their effects on nurses’ health, turnover intent, team effectiveness, and patient safety: a longitudinal questionnaire study. Scand J Work Environ Health. (2024) 50:113–21. doi: 10.5271/sjweh.4138, PMID: 38232184 PMC10928545

[3] Salyers MP Bonfils KA Luther L Firmin RL White DA Adams EL . The relationship between professional burnout and quality and safety in healthcare: a Meta-analysis. J Gen Intern Med. (2017) 32:475–82. doi: 10.1007/s11606-016-3886-9, PMID: 27785668 PMC5377877

[4] Wallace JE. Burnout, coping and suicidal ideation: an application and extension of the job demand-control-support model. J Work Behav Health. (2017) 32:99–118. doi: 10.1080/15555240.2017.1329628

[5] Wei H Sewell KA Woody G Rose MA. The state of the science of nurse work environments in the United States: a systematic review. Int J Nurs Sci. (2018) 5:287–300. doi: 10.1016/j.ijnss.2018.04.010, PMID: 31406839 PMC6626229

[6] West CP Dyrbye LN Shanafelt TD. Physician burnout: contributors, consequences and solutions. J Intern Med. (2018) 283:516–29. doi: 10.1111/joim.12752, PMID: 29505159

[7] EU-OHSA. (2023). Psychosocial risk management in the health and social sector. Discussion paper. Available online at: https://osha.europa.eu/en/publications/psychosocial-risks-health-and-social-care-sector_risk_management_social_care_en_.pdf (Accessed September 6, 2024)

[8] Eurofund. Working conditions and workers' health. Luxembourg: Eurofund (2019).

[9] Johnson J Hall LH Berzins K Baker J Melling K Thompson C. Mental healthcare staff well-being and burnout: a narrative review of trends, causes, implications, and recommendations for future interventions. Int J Ment Health Nurs. (2018) 27:20–32. doi: 10.1111/inm.12416, PMID: 29243348

[10] Rodrigues H Cobucci R Oliveira A Cabral JV Medeiros L Gurgel K . Burnout syndrome among medical residents: a systematic review and meta-analysis. PloS One. (2018) 13:e0206840. doi: 10.1371/journal.pone.0206840, PMID: 30418984 PMC6231624

[11] Pekkarinen L Elovainio M Sinervo T Heponiemi T Aalto AM Noro A . Job demands and musculoskeletal symptoms among female geriatric nurses: the moderating role of psychosocial resources. J Occup Health Psychol. (2013) 18:211–9. doi: 10.1037/a0031801, PMID: 23458058

[12] Adriaenssens J De Gucht V Maes S. Determinants and prevalence of burnout in emergency nurses: a systematic review of 25 years of research. Int J Nurs Stud. (2015) 52:649–61. doi: 10.1016/j.ijnurstu.2014.11.004, PMID: 25468279

[13] Harvey SB Epstein RM Glozier N Petrie K Strudwick J Gayed A . Mental illness and suicide among physicians. Lancet. (2021) 398:920–30. doi: 10.1016/S0140-6736(21)01596-8, PMID: 34481571 PMC9618683

[14] Bakker AB Demerouti E. Job demands-resources theory: taking stock and looking forward. J Occup Health Psychol. (2017) 22:273–85. doi: 10.1037/ocp0000056, PMID: 27732008

[15] WHO. (2024) Health workforce. Avalible at: https://www.who.int/health-topics/health-workforce#tab=tab_1 (Accessed September 6, 2024)

[16] Aust B Leduc C Cresswell-Smith J O'Brien C Rugulies R Leduc M . The effects of different types of organisational workplace mental health interventions on mental health and wellbeing in healthcare workers: a systematic review. Int Arch Occup Environ Health. (2024) 97:485–522. doi: 10.1007/s00420-024-02065-z, PMID: 38695906 PMC11130054

[17] LaMontagne AD Shann C Martin A. Developing an integrated approach to workplace mental health: a hypothetical conversation with a small business owner. Ann Work Exposures Health. (2018) 62:S93–s100. doi: 10.1093/annweh/wxy039, PMID: 30212883

[18] Rugulies R Aust B Greiner B Arensman E Kawakami N LaMontagne A . Work-related causes of mental health conditions and interventions for their improvement in workplaces. Lancet. (2023) 402:1368–81. doi: 10.1016/S0140-6736(23)00869-3, PMID: 37838442

[19] Teoh K Dhensa-Kahlon R Christensen M Frost F Hatton E Nielsen K. Organizational wellbeing interventions: Case studies from the NHS. London: University of London (2023).

[20] Giusino D De Angelis M Mazzetti G Faiulo IR Innstrand ST Christensen M . Mentally healthy healthcare: main findings and lessons learned from a needs assessment exercise at multiple workplace levels In: Bowers CA Beidel DC Marks MR Horan K Cannon-Bowers J, editors. Mental health and wellness in healthcare workers: Identifying risks, prevention, and treatment IGI global. Hershey, PA: Information Resources Management Association (2022). 143–71.

[21] von Thiele SU Nielsen K Edwards K Hasson H Ipsen C Savage C . How to design, implement and evaluate organizational interventions for maximum impact: the Sigtuna principles. Eur J Work Organ Psychol. (2021) 30:415–27. doi: 10.1080/1359432X.2020.1803960, PMID: 34518756 PMC8432268

[22] Alabi RO Hietanen P Elmusrati M Youssef O Almangush A Mäkitie AA. Mitigating burnout in an oncological unit: a scoping review. Front Public Health. (2021) 9:9. doi: 10.3389/fpubh.2021.677915, PMID: 34660505 PMC8517258

[23] Dall'Ora C Ball J Reinius M Griffiths P. Burnout in nursing: a theoretical review. Hum Resour. Health. (2020) 18:469. doi: 10.1186/s12960-020-00469-9, PMID: 32503559 PMC7273381

[24] McCormack HM Mac Intyre TE O'Shea D Herring MP Campbell MJ. The prevalence and cause (s) of burnout among applied psychologists: a systematic review. Front Psychol. (2018) 9:1897. doi: 10.3389/fpsyg.2018.01897, PMID: 30386275 PMC6198075

[25] O'Connor K Muller Neff D Pitman S. Burnout in mental health professionals: a systematic review and meta-analysis of prevalence and determinants. Eur Psychiatry. (2018) 53:74–99. doi: 10.1016/j.eurpsy.2018.06.003, PMID: 29957371

[26] Jacquier-Bret J Gorce P. Prevalence of body area work-related musculoskeletal disorders among healthcare professionals: a systematic review. Int J Environ Res Public Health. (2023) 20:841. doi: 10.3390/ijerph20010841, PMID: 36613163 PMC9819551

[27] Lietz J Kozak A Nienhaus A. Prevalence and occupational risk factors of musculoskeletal diseases and pain among dental professionals in Western countries: a systematic literature review and meta-analysis. PloS One. (2018) 13:e0208628. doi: 10.1371/journal.pone.0208628, PMID: 30562387 PMC6298693

[28] Le Floch B Bastiaens H Le Reste JY Lingner H Hoffman RD Czachowski S . Which positive factors determine the GP satisfaction in clinical practice? A systematic literature review. BMC Fam Pract. (2016) 17:133. doi: 10.1186/s12875-016-0524-x, PMID: 27619913 PMC5020554

[29] Zangaro GA Soeken KL. A meta-analysis of studies of nurses' job satisfaction. Res Nurs Health. (2007) 30:445–58. doi: 10.1002/nur.20202, PMID: 17654483

[30] Ajslev JZN Møller JL Andersen MF Pirzadeh P Lingard H. The hierarchy of controls as an approach to visualize the impact of occupational safety and health coordination. Int J Environ Res Public Health. (2022) 19:2731. doi: 10.3390/ijerph19052731, PMID: 35270423 PMC8910555

[31] Montano D Hoven H Siegrist J. Effects of organisational-level interventions at work on employees' health: a systematic review. BMC Public Health. (2014) 14:135. doi: 10.1186/1471-2458-14-135, PMID: 24507447 PMC3929163

[32] Laugesen K Ludvigsson JF Schmidt M Gissler M Valdimarsdottir UA Lunde A . Nordic health registry-based research: a review of health care systems and key registries. Clin Epidemiol. (2021) 13:533–54. doi: 10.2147/CLEP.S314959, PMID: 34321928 PMC8302231

[33] Grant MJ Booth A. A typology of reviews: an analysis of 14 review types and associated methodologies. Health Inf Libr J. (2009) 26:91–108. doi: 10.1111/j.1471-1842.2009.00848.x, PMID: 19490148

[34] Page MJ McKenzie JE Bossuyt PM Boutron I Hoffmann TC Mulrow CD . The PRISMA 2020 statement: an updated guideline for reporting systematic reviews. BMJ. (2021) 372:n71. doi: 10.1136/bmj.n71, PMID: 33782057 PMC8005924

[35] Cooke A Smith D Booth A. Beyond PICO: the SPIDER tool for qualitative evidence synthesis. Qual Health Res. (2012) 22:1435–43. doi: 10.1177/1049732312452938, PMID: 22829486

[36] Hong QN Gonzalez-Reyes A Pluye P. Improving the usefulness of a tool for appraising the quality of qualitative, quantitative and mixed methods studies, the mixed methods appraisal tool (MMAT). J Eval Clin Pract. (2018) 24:459–67. doi: 10.1111/jep.12884, PMID: 29464873

[37] Braun V Clarke V. Using thematic analysis in psychology. Qual Res Psychol. (2006) 3:77–101. doi: 10.1191/1478088706qp063oa

[38] Bigert C Kader M Andersson T Selander J Bodin T Gustavsson P . Night and shift work and incidence of cerebrovascular disease – a prospective cohort study of healthcare employees in Stockholm. Scand J Work Environ Health. (2022) 48:31–40. doi: 10.5271/sjweh.3986, PMID: 34557927 PMC8729165

[39] Waage S Pallesen S Moen BE Vedaa Ø Thun E Vikanes Buchvold H . Changes in work schedule affect the prevalence of shift work disorder among Norwegian nurses—a two year follow-up study. Chronobiol Int. (2021) 38:924–32. doi: 10.1080/07420528.2021.1896535, PMID: 33736559

[40] Härmä M Karhula K Puttonen S Ropponen A Koskinen A Ojajärvi A . Shift work with and without night work as a risk factor for fatigue and changes in sleep length: a cohort study with linkage to records on daily working hours. J Sleep Res. (2019) 28:e12658. doi: 10.1111/jsr.12658, PMID: 29383788

[41] Kader M Selander J Andersson T Albin M Bodin T Härmä M . Night and shift work characteristics and incident ischemic heart disease and atrial fibrillation among healthcare employees – a prospective cohort study. Scand J Work Environ Health. (2022) 48:520–9. doi: 10.5271/sjweh.4045, PMID: 35723926 PMC10539110

[42] Hansen AB Stayner L Hansen J Andersen ZJ. Night shift work and incidence of diabetes in the Danish nurse cohort. Occup Environ Med. (2016) 73:262–8. doi: 10.1136/oemed-2015-103342, PMID: 26889020

[43] Bernstrøm VH Houkes I. Shift work and sickness absence at a Norwegian hospital: a longitudinal multilevel study. Occup Environ Med. (2020) 77:555–63. doi: 10.1136/oemed-2019-106240, PMID: 32327467

[44] Vedaa Ø Harris A Erevik EK Waage S Bjorvatn B Sivertsen B . Short rest between shifts (quick returns) and night work is associated with work-related accidents. Int Arch Occup Environ Health. (2019) 92:829–35. doi: 10.1007/s00420-019-01421-8, PMID: 30879132

[45] Härmä M Karhula K Ropponen A Puttonen S Koskinen A Ojajärvi A . Association of changes in work shifts and shift intensity with change in fatigue and disturbed sleep: a within-subject study. Scand J Work Environ Health. (2018) 44:394–402. doi: 10.5271/sjweh.3730, PMID: 29641837

[46] Larsen AD Ropponen A Hansen J Hansen ÅM Kolstad HA Koskinen A . Working time characteristics and long-term sickness absence among Danish and Finnish nurses: a register-based study. Int J Nurs Stud. (2020) 112:103639. doi: 10.1016/j.ijnurstu.2020.103639, PMID: 32505388

[47] Ropponen A Koskinen A Puttonen S Härmä M. Exposure to working-hour characteristics and short sickness absence in hospital workers: a case-crossover study using objective data. Int J Nurs Stud. (2019) 91:14–21. doi: 10.1016/j.ijnurstu.2018.11.002, PMID: 30665013

[48] Kader M Bigert C Andersson T Selander J Bodin T Skröder H . Shift and night work during pregnancy and preterm birth – a cohort study of Swedish health care employees. Int J Epidemiol. (2021) 50:1864–74. doi: 10.1093/ije/dyab135, PMID: 34999871 PMC8743126

[49] Jørgensen JT Hansen J Westendorp RGJ Nabe-Nielsen K Stayner LT Simonsen MK . Shift work and incidence of dementia: a Danish nurse cohort study. Alzheimers Dement. (2020) 16:1268–79. doi: 10.1002/alz.12126, PMID: 32652788

[50] Jørgensen JT Schernhammer E Papantoniou K Hansen J Westendorp RGJ Stayner L . Night work and incidence of Parkinson's disease in the Danish nurse cohort. Occup Environ Med. (2021) 78:419–25. doi: 10.1136/oemed-2020-107067, PMID: 33323454

[51] Erdem JS Notø HØ Skare Ø Lie JAS Petersen-øverleir M Reszka E . Mechanisms of breast cancer risk in shift workers: association of telomere shortening with the duration and intensity of night work. Cancer Med. (2017) 6:1988–97. doi: 10.1002/cam4.1135, PMID: 28707432 PMC5548875

[52] Hammer P Hageman I Garde A Begtrup L Flachs E Hansen J . Night work and postpartum depression: a national register-based cohort study. Scand J Work Environ Health. (2019) 45:577–87. doi: 10.5271/sjweh.3831, PMID: 31125110

[53] Persson SS Lindström PN Pettersson P Andersson I. Workplace relationships impact self-rated health: a survey of Swedish municipal health care employees. Work J Prevent Assess Rehabil. (2018) 60:85–94. doi: 10.3233/WOR-182721, PMID: 29843296

[54] Cheng W-J Puttonen S Vanttola P Koskinen A Kivimäki M Härmä M. Association of shift work with mood disorders and sleep problems according to chronotype: a 17-year cohort study. Chronobiol Int. (2021) 38:518–25. doi: 10.1080/07420528.2021.1885431, PMID: 33588657

[55] Henriksen L Lukasse M. Burnout among Norwegian midwives and the contribution of personal and work-related factors: a cross-sectional study. Sex Reprod Healthc. (2016) 9:42–7. doi: 10.1016/j.srhc.2016.08.001, PMID: 27634664

[56] Ropponen A Koskinen A Puttonen S Härmä M. A case-crossover study of age group differences in objective working-hour characteristics and short sickness absence. J Nurs Manag. (2020) 28:787–96. doi: 10.1111/jonm.12992, PMID: 32145050

[57] Nielsen HB Dyreborg J Hansen ÅM Hansen J Kolstad HA Larsen AD . Shift work and risk of occupational, transport and leisure-time injury. A register-based case-crossover study of Danish hospital workers. Saf Sci. (2019) 120:728–34. doi: 10.1016/j.ssci.2019.07.006

[58] Nielsen HB Hansen ÅM Conway SH Dyreborg J Hansen J Kolstad HA . Short time between shifts and risk of injury among danish hospital workers: a register-based cohort study. Scand J Work Environ Health. (2019) 45:166–73. doi: 10.5271/sjweh.3770, PMID: 30264848

[59] Jørgensen JT Rozing MP Westendorp RGJ Hansen J Stayner LT Simonsen MK . Shift work and incidence of psychiatric disorders: the Danish nurse cohort study. J Psychiatr Res. (2021) 139:132–8. doi: 10.1016/j.jpsychires.2021.05.045, PMID: 34058652

[60] Jørgensen JT Karlsen S Stayner L Hansen J Andersen ZJ. Shift work and overall and cause-specific mortality in the Danish nurse cohort. Scand J Work Environ Health. (2017) 43:117–26. doi: 10.5271/sjweh.3612, PMID: 28245504

[61] Jensen HI Larsen JW Thomsen TD. The impact of shift work on intensive care nurses’ lives outside work: a cross-sectional study. J Clin Nurs. (2018) 27:e703–9. doi: 10.1111/jocn.14197, PMID: 29193498

[62] Karhula K Hakola T Koskinen A Ojajärvi A Kivimäki M Härmä M. Permanent night workers´ sleep and psychosocial factors in hospital work. A comparison to day and shift work. Chronobiol Int. (2018) 35:785–94. doi: 10.1080/07420528.2018.1466792, PMID: 29764221

[63] Grasmo SG Liaset IF Redzovic SE. Home care workers’ experiences of work conditions related to their occupational health: a qualitative study. BMC Health Serv Res. (2021) 21:962. doi: 10.1186/s12913-021-06941-z, PMID: 34521407 PMC8438557

[64] Ropponen A Koskinen A Puttonen S Ervasti J Kivimäki M Oksanen T . Association of working hour characteristics and on-call work with risk of short sickness absence among hospital physicians: a longitudinal cohort study. Chronobiol Int. (2022) 39:233–40. doi: 10.1080/07420528.2021.1993238, PMID: 34724854

[65] Ropponen A Gluschkoff K Ervasti J Kivimäki M Koskinen A Krutova O . Working hour patterns and risk of occupational accidents. An optimal matching analysis in a hospital employee cohort. Saf Sci. (2023) 159:106004. doi: 10.1016/j.ssci.2022.106004, PMID: 39699678

[66] Härmä M Koskinen A Sallinen M Kubo T Ropponen A Lombardi DA. Characteristics of working hours and the risk of occupational injuries among hospital employees: a case-crossover study. Scand J Work Environ Health. (2020) 46:570–8. doi: 10.5271/sjweh.3905, PMID: 32515483 PMC7737806

[67] Ose SO Tjønnås MS Kaspersen SL Færevik H. One-year trial of 12-hour shifts in a non-intensive care unit and an intensive care unit in a public hospital: a qualitative study of 24 nurses' experiences. BMJ Open. (2019) 9:e024292. doi: 10.1136/bmjopen-2018-024292, PMID: 31289050 PMC6629459

[68] Møller CM Clausen T Aust B Eiberg JP. A cross-sectional national study of burnout and psychosocial work environment in vascular surgery in Denmark. J Vasc Surg. (2022) 75:1750–9.e3. doi: 10.1016/j.jvs.2021.11.042, PMID: 34788647

[69] Dahlgren A Tucker P Bujacz A Frögéli E Rudman A Gustavsson P. Intensive longitudinal study of newly graduated nurses’ quick returns and self-rated stress. Scand J Work Environ Health. (2021) 47:404–7. doi: 10.5271/sjweh.3962, PMID: 33929547 PMC8259702

[70] Vedaa Ø Pallesen S Waage S Bjorvatn B Sivertsen B Erevik E . Short rest between shift intervals increases the risk of sick leave: a prospective registry study. Occup Environ Med. (2017) 74:496–501. doi: 10.1136/oemed-2016-103920, PMID: 27827302

[71] Beltagy MS Pentti J Vahtera J Kivimäki M. Night work and risk of common mental disorders: analyzing observational data as a non-randomized pseudo trial. Scand J Work Environ Health. (2018) 44:512–20. doi: 10.5271/sjweh.3733, PMID: 29870046

[72] Vedaa Ø Harris A Waage S Bjorvatn B Thun E Buchvold HV . A longitudinal study on the association between quick returns and occupational accidents. Scand J Work Environ Health. (2020) 46:645–9. doi: 10.5271/sjweh.3906, PMID: 32632456 PMC7737807

[73] Seitovirta J Vehviläinen-Julkunen K Mitronen L De Gieter S Kvist T. Attention to nurses’ rewarding—an interview study of registered nurses working in primary and private healthcare in Finland. J Clin Nurs. (2017) 26:1042–52. doi: 10.1111/jocn.13459, PMID: 27346394

[74] Loft MI Jensen CS. What makes experienced nurses stay in their position? A qualitative interview study. J Nurs Manag. (2020) 28:1305–16. doi: 10.1111/jonm.13082, PMID: 32589776

[75] Ruotsalainen S Jantunen S Sinervo T. Which factors are related to Finnish home care workers' job satisfaction, stress, psychological distress and perceived quality of care?- a mixed method study. BMC Health Serv Res. (2020) 20:896. doi: 10.1186/s12913-020-05733-1, PMID: 32988396 PMC7520953

[76] Westergren E Lindberg M. Work-related musculoskeletal complaints among haemodialysis nurses: an exploratory study of the work situation from an ergonomic perspective. Work. (2022) 72:875–84. doi: 10.3233/WOR-205241, PMID: 35634816 PMC9398067

[77] Cohidon C Wild P Senn N. Practice organization characteristics related to job satisfaction among general practitioners in 11 countries. Ann Fam Med. (2019) 17:510–7. doi: 10.1370/afm.2449, PMID: 31712289 PMC6846274

[78] Kjellström S Avby G Areskoug-Josefsson K Andersson Gäre B Andersson BM. Work motivation among healthcare professionals: a study of well-functioning primary healthcare centers in Sweden. J Health Organ Manage. (2017) 31:487–502. doi: 10.1108/JHOM-04-2017-0074, PMID: 28877624

[79] Herttuala N Kokkinen L Konu A. Social-and healthcare managers' work wellbeing—literature review and key informant interviews. Int J Workplace Health Manag. (2020) 13:633–48. doi: 10.1108/IJWHM-05-2019-0077

[80] Ruotsalainen S Elovainio M Jantunen S Sinervo T. The mediating effect of psychosocial factors in the relationship between self-organizing teams and employee wellbeing: a cross-sectional observational study. Int J Nurs Stud. (2023) 138:104415. doi: 10.1016/j.ijnurstu.2022.104415, PMID: 36527858

[81] Gamskjaer T Werlauff U Handberg C. Investigating job satisfaction in palliative rehabilitation: reflections and perspectives of health professionals working with amyotrophic lateral sclerosis. J Eval Clin Pract. (2022) 28:108–19. doi: 10.1111/jep.13599, PMID: 34269500

[82] Thun S Halsteinli V Løvseth L. A study of unreasonable illegitimate tasks, administrative tasks, and sickness presenteeism amongst Norwegian physicians: an everyday struggle? BMC Health Serv Res. (2018) 18:407. doi: 10.1186/s12913-018-3229-0, PMID: 29871623 PMC5989409

[83] Svedahl ER Pape K Toch-Marquardt M Skarshaug LJ Kaspersen S-L Bjørngaard JH . Increasing workload in Norwegian general practice – a qualitative study. BMC Fam Pract. (2019) 20:1–10. doi: 10.1186/s12875-019-0952-5, PMID: 31113368 PMC6530128

[84] Johnsen TM Norberg BL Krogh FH Vonen HD Getz O Austad B. The impact of clinical experience on working tasks and job-related stress: a survey among 1032 Norwegian GPs. BMC Primary Care. (2022) 23:1–10. doi: 10.1186/s12875-022-01810-y, PMID: 36030207 PMC9419378

[85] Rantanen A Pitkänen A Paimensalo-Karell I Elovainio M Aalto P. Two models of nursing practice: a comparative study of motivational characteristics, work satisfaction and stress. J Nurs Manag. (2016) 24:261–70. doi: 10.1111/jonm.12313, PMID: 26014618

[86] Riisgaard H Søndergaard J Munch M Le JV Ledderer L Pedersen LB . Associations between degrees of task delegation and job satisfaction of general practitioners and their staff: a cross-sectional study. BMC Health Serv Res. (2017) 17:44. doi: 10.1186/s12913-017-1984-y, PMID: 28095846 PMC5240386

[87] Jepsen I Juul S Foureur M Sørensen EE Nøhr EA. Is caseload midwifery a healthy work-form? – a survey of burnout among midwives in Denmark. Sex Reprod Healthc. (2017) 11:102–6. doi: 10.1016/j.srhc.2016.12.001, PMID: 28159119

[88] Jepsen I Mark E Aagaard Nøhr E Foureur M Elgaard SE. A qualitative study of how caseload midwifery is constituted and experienced by Danish midwives. Midwifery. (2016) 36:61–9. doi: 10.1016/j.midw.2016.03.002, PMID: 27106945

[89] Kaltenbrunner M Bengtsson L Mathiassen SE Högberg H Engström M. Staff perception of lean, care-giving, thriving and exhaustion: a longitudinal study in primary care. BMC Health Serv Res. (2019) 19:652. doi: 10.1186/s12913-019-4502-6, PMID: 31500624 PMC6734292

[90] Lunde L-K Merkus S Koch M Knardahl S Wærsted M Veiersted KB. Associations of objectively measured total duration and maximum bout length of standing at work with lower-extremity pain intensity: a 2-year follow-up of construction and healthcare workers. BMC Musculoskelet Disord. (2021) 22:1–11. doi: 10.1186/s12891-020-03868-0 33413254 PMC7791765

[91] Olsen E Bjaalid G Mikkelsen A. Work climate and the mediating role of workplace bullying related to job performance, job satisfaction, and work ability: a study among hospital nurses. J Adv Nurs. (2017) 73:2709–19. doi: 10.1111/jan.13337, PMID: 28512986

[92] Nielsen MS Jørgensen F. Meaning creation and employee engagement in home health caregivers. Scand J Caring Sci. (2016) 30:57–64. doi: 10.1111/scs.12221, PMID: 25982838

[93] Pedersen LB Allen T Waldorff FB Andersen MKK. Does accreditation affect the job satisfaction of general practitioners? A combined panel data survey and cluster randomised field experiment. Health Policy. (2020) 124:849–55. doi: 10.1016/j.healthpol.2020.04.002, PMID: 32540210

[94] Fallman SL Dellve L Kullén EA. Managerial approaches for maintaining low levels of sick leave: a qualitative study. J Nurs Manage. (2022) 30:3546–52. doi: 10.1111/jonm.13678, PMID: 35560674 PMC10084398

[95] Ahlstedt C Eriksson Lindvall C Holmström IK Muntlin AÅ. What makes registered nurses remain in work? An ethnographic study. Int J Nurs Stud. (2019) 89:32–8. doi: 10.1016/j.ijnurstu.2018.09.008, PMID: 30339953

[96] Slåtten T Lien G Mutonyi BR. Precursors and outcomes of work engagement among nursing professionals—a cross-sectional study. BMC Health Serv Res. (2022) 22:21. doi: 10.1186/s12913-021-07405-0, PMID: 34983510 PMC8725263

[97] Vifladt A Simonsen BO Lydersen S Farup PG. The association between patient safety culture and burnout and sense of coherence: a cross-sectional study in restructured and not restructured intensive care units. Intensive Crit Care Nurs. (2016) 36:26–34. doi: 10.1016/j.iccn.2016.03.004, PMID: 27212614

[98] Thapa DR Ekström-Bergström A Krettek A Areskoug-Josefsson K. Support and resources to promote and sustain health among nurses and midwives in the workplace: a qualitative study. Nordic J Nurs Res. (2021) 41:166–74. doi: 10.1177/2057158520988452

[99] Jacobsen CB Hansen AKL Pedersen LD. Not too narrow, not too broad: linking span of control, leadership behavior, and employee job satisfaction in public organizations. Public Adm Rev. (2022) 83:775–92. doi: 10.1111/puar.13566, PMID: 39696797

[100] Sigursteinsdóttir H Skúladóttir H Agnarsdóttir T Halldórsdóttir S. Stressful factors in the working environment, lack of adequate sleep, and musculoskeletal pain among nursing unit managers. Int J Environ Res Public Health. (2020) 17:673. doi: 10.3390/ijerph17020673, PMID: 31968675 PMC7014039

[101] Andersen LL Vinstrup J Villadsen E Jay K Jakobsen MD. Physical and psychosocial work environmental risk factors for back injury among healthcare workers: prospective cohort study. Int J Environ Res Public Health. (2019) 16:4528. doi: 10.3390/ijerph16224528, PMID: 31731806 PMC6887976

[102] Vinstrup J Jakobsen MD Madeleine P Andersen LL. Physical exposure during patient transfer and risk of back injury & low-back pain: prospective cohort study. BMC Musculoskelet Disord. (2020) 21:715. doi: 10.1186/s12891-020-03731-2, PMID: 33129282 PMC7603727

[103] Lindegård A Nordander C Jacobsson H Arvidsson I. Opting to wear prismatic spectacles was associated with reduced neck pain in dental personnel: a longitudinal cohort study. BMC Musculoskelet Disord. (2016) 17:347. doi: 10.1186/s12891-016-1145-1, PMID: 27535742 PMC4989289

[104] Westergren E Ludvigsen MS Lindberg M. Associations between materials used and work-related musculoskeletal hand complaints among haemodialysis nurses. J Ren Care. (2020) 46:185–92. doi: 10.1111/jorc.12317, PMID: 32030898

[105] Lee SY Hung L Chaudhury H Morelli A. Staff perspectives on the role of physical environment in long-term care facilities on dementia care in Canada and Sweden. Dementia. (2021) 20:2558–72. doi: 10.1177/14713012211003994, PMID: 33780287

[106] Golvani J Roos L Henricson M. Operating room nurses’ experiences of limited access to daylight in the workplace – a qualitative interview study. BMC Nurs. (2021) 20:227. doi: 10.1186/s12912-021-00751-8, PMID: 34753467 PMC8579627

[107] Vilén L Atosuo J Putus T. Prevalence of hoarseness in primary health care and hospitals—associations with different work tasks and environmental factors among nurses. J Voice. (2022) 38:1253.e29–1253.e34. doi: 10.1016/j.jvoice.2022.02.024 35365386

[108] Vilén L Putus T. Hoarseness among nurses. J Voice. (2021) 37:798.e15–798.e18. doi: 10.1016/j.jvoice.2021.03.030 34016510

[109] Kjørstad K Faaland PM Sivertsen B Kallestad H Langsrud K Vethe D . Sleep and work functioning in nurses undertaking inpatient shifts in a blue-depleted light environment. BMC Nurs. (2022) 21:1–10. doi: 10.1186/s12912-022-00973-4, PMID: 35850690 PMC9290304

[110] Golay D Salminen Karlsson M Cajander Å. Effortlessness and security: Nurses' positive experiences with work-related information technology use. CIN Comput Informatics Nurs. (2022) 40:589–97. doi: 10.1097/CIN.0000000000000917, PMID: 35475766 PMC9470047

[111] Stadin M Nordin M Fransson EI Broström A. Healthcare managers' experiences of technostress and the actions they take to handle it – a critical incident analysis. BMC Med Informatics Decis Mak. (2020) 20:244. doi: 10.1186/s12911-020-01261-4, PMID: 32977817 PMC7517792

[112] Golay D Salminen Karlsson M Cajander Å. Negative emotions induced by work-related information technology use in hospital nursing. CIN Comput Informatics Nurs. (2022) 40:113–20. doi: 10.1097/CIN.0000000000000800, PMID: 34347645 PMC8820768

[113] Heponiemi T Hyppönen H Vehko T Kujala S Aalto A-M Vänskä J . Finnish physicians' stress related to information systems keeps increasing: a longitudinal three-wave survey study. BMC Med Informatics Decision Making. (2017) 17:1–8. doi: 10.1186/s12911-017-0545-y, PMID: 29041971 PMC5646125

[114] Vainiomäki S Heponiemi T Vänskä J Hyppönen H. Tailoring ehrs for specific working environments improves work well-being of physicians. Int J Environ Res Public Health. (2020) 17:1–10. doi: 10.3390/ijerph17134715, PMID: 32630043 PMC7369852

[115] Heponiemi T Kaihlanen AM Gluschkoff K Saranto K Nissinen S Laukka E . The association between using a mobile version of an electronic health record and the well-being of nurses: cross-sectional survey study. JMIR Med Inform. (2021) 9:e28729. doi: 10.2196/28729, PMID: 34255704 PMC8292939

[116] Heponiemi T Kujala S Vainiomäki S Vehko T Lääveri T Vänskä J . Usability factors associated with physicians' distress and information system-related stress: cross-sectional survey. JMIR Med Inform. (2019) 7:e13466. doi: 10.2196/13466, PMID: 31687938 PMC6913751

[117] Holmberg C Sobis I Carlström E. Job satisfaction among Swedish mental health nursing staff: a cross-sectional survey. Int J Public Adm. (2016) 39:429–36. doi: 10.1080/01900692.2015.1018432

[118] Rosenström T Härmä M Kivimäki M Ervasti J Virtanen M Hakola T . Patterns of working hour characteristics and risk of sickness absence among shift-working hospital employees: a data-mining cohort study. Scand J Work Environ Health. (2021) 47:395–403. doi: 10.5271/sjweh.3957, PMID: 33971014 PMC8259704

[119] Hult M Halminen O Mattila-Holappa P Kangasniemi M. Health and work well-being associated with employment precariousness among permanent and temporary nurses: a cross-sectional survey. Nordic J Nurs Res. (2022) 42:140–6. doi: 10.1177/20571585211070376

[120] Grønstad A Kjekshus LE Tjerbo T Bernstrøm VH. Work-related moderators of the relationship between organizational change and sickness absence: a longitudinal multilevel study. BMC Public Health. (2020) 20:1218. doi: 10.1186/s12889-020-09325-w, PMID: 32770987 PMC7414577

[121] Blomberg K Isaksson AK Allvin R Bisholt B Ewertsson M Kullén Engström A . Work stress among newly graduated nurses in relation to workplace and clinical group supervision. J Nurs Manage. (2016) 24:80–7. doi: 10.1111/jonm.12274 25421164

[122] Spännargård Å Fagernäs S Alfonsson S. Self-perceived clinical competence, gender and workplace setting predict burnout among psychotherapists. Couns Psychother Res. (2022) 23:469–77. doi: 10.1002/capr.12532, PMID: 39699678

[123] Liss A Alian AY Wennström JL Abrahamsson KH. Professional competencies and work-related support in relation to periodontal therapy and work satisfaction: a questionnaire study among Swedish dental hygienists. Int J Dent Hyg. (2018) 16:349–56. doi: 10.1111/idh.12324, PMID: 29143453

[124] Jakobsen MD Sundstrup E Brandt M Andersen LL. Effect of physical exercise on musculoskeletal pain in multiple body regions among healthcare workers: secondary analysis of a cluster randomized controlled trial. Musculoskelet Sci Pract. (2018) 34:89–96. doi: 10.1016/j.msksp.2018.01.006, PMID: 29414757

[125] Ose SO Færevik H Håpnes T Øyum L. Perceived causes of work-related sick leave among hospital nurses in Norway: a Prepandemic study. Saf Health Work. (2022) 13:350–6. doi: 10.1016/j.shaw.2022.04.002, PMID: 36156869 PMC9482014

[126] Mauno S Ruokolainen M Kinnunen U De Bloom J. Emotional labour and work engagement among nurses: examining perceived compassion, leadership and work ethic as stress buffers. J Adv Nurs. (2016) 72:1169–81. doi: 10.1111/jan.12906, PMID: 26841277

[127] Grønstad A Kjekshus LE Tjerbo T Bernstrøm VH. Organizational change and the risk of sickness absence: a longitudinal multilevel analysis of organizational unit-level change in hospitals. BMC Health Serv Res. (2019) 19:895. doi: 10.1186/s12913-019-4745-2, PMID: 31771576 PMC6880570

[128] Fallman SL Jutengren G Dellve L. The impact of restricted decision-making autonomy on health care managers’ health and work performance. J Nurs Manag. (2019) 27:706–14. doi: 10.1111/jonm.12741, PMID: 30565780

[129] Poikkeus T Suhonen R Katajisto J Leino-Kilpi H. Relationships between organizational and individual support, nurses' ethical competence, ethical safety, and work satisfaction. Health Care Manag Rev. (2020) 45:83–93. doi: 10.1097/HMR.0000000000000195, PMID: 29533273

[130] Burgess MG Brough P Biggs A Hawkes AJ. Why interventions fail: a systematic review of occupational health psychology interventions. Int J Stress Manag. (2020) 27:195–207. doi: 10.1037/str0000144

[131] Karanika-Murray M Biron C Saksvik P. Organizational health interventions: advances in evaluation methodology. Stress Health. (2016) 32:255–7. doi: 10.1002/smi.2708, PMID: 27681037

[132] Kecklund G Axelsson J. Health consequences of shift work and insufficient sleep. BMJ. (2016) 355:i 5210. doi: 10.1136/bmj.i5210, PMID: 27803010

[133] Rosa D Terzoni S Dellafiore F Destrebecq A. Systematic review of shift work and nurses' health. Occup Med. (2019) 69:237–43. doi: 10.1093/occmed/kqz063, PMID: 31132107

[134] Schneider A Weigl M. Associations between psychosocial work factors and provider mental well-being in emergency departments: a systematic review. PloS One. (2018) 13:e0197375. doi: 10.1371/journal.pone.0197375, PMID: 29864128 PMC5986127

[135] Daouk-Öyry L Anouze AL Otaki F Dumit NY Osman I. The JOINT model of nurse absenteeism and turnover: a systematic review. Int J Nurs Stud. (2014) 51:93–110. doi: 10.1016/j.ijnurstu.2013.06.018, PMID: 23928322

[136] Oh Y Gastmans C. Moral distress experienced by nurses: a quantitative literature review. Nurs Ethics. (2015) 22:15–31. doi: 10.1177/0969733013502803, PMID: 24091351

[137] Gyllensten K Andersson G Muller H. Experiences of reduced work hours for nurses and assistant nurses at a surgical department: A qualitaive study. BMC Nurs. (2017) 16. doi: 10.1186/s12912-017-0210-x PMC5379756 28396616

[138] Karhula K Turunen J Hakola T Ojajärvi A Puttonen S Ropponen A . The effects of using participatory working time scheduling software on working hour characteristics and wellbeing: A quasi-experimental study of irregular shift work. Int J Nurs Stud. (2020) 112:9. doi: 10.1016/j.ijnurstu.2020.103696 32800568

[139] Vedaa Ø Mørland E Larsen M Harris A Erevik E Sivertsen B . Sleep detriments associated with quick returns in rotating shift work: A diary study. J Occup Environ Med. (2017) 59:522–7. doi: 10.1097/JOM.0000000000001006 28437294

