# Supplementary material for: Organisational-level risk and health-promoting factors within the healthcare sector—a systematic search and review
Source: Front Med (Lausanne). 2025 Jan 17;11:1509023. doi: 10.3389/fmed.2024.1509023 (PMC11783186; doi:10.3389/fmed.2024.1509023)
Supplement: Supplementary file 1 [file Table_1.docx]

| Reference, year | Design | Professional groups | Country | Study aim | Category^a^ | Risk or health-promoting factor^b^ | Outcome |
| --- | --- | --- | --- | --- | --- | --- | --- |
| Ahlstedt et al. 2019 (95) | Qualitative | Registered nurses | Sweden | To explore registered nurses’ workday events in relation to inner work life theory to better understand what influences registered nurses to remain in work. | 2, 4 | H | Motivation |
| Andersen et al. 2019 (101) | Cohort | Healthcare workers | Denmark | To investigate physical and psychosocial work environmental risk factors for back injury during patient transfer among healthcare workers in hospitals. | 3 | R | Back injury |
| Beltagy et al. 2018 (71) | Cohort | Healthcare workers | Finland | To examine the status of night work as a risk factor for common mental disorders | 1 | R | Common mental disorders |
| Bernstrøm and Houkes 2020 (43) | Cohort | Healthcare workers | Norway | (1) To describe what shift-work arrangements exist at a large Norwegian hospital, (2) to investigate how these shift schedules relate to employees’ sickness absence, and (3) to investigate how individual differences in age, gender, and parental status moderate the relationship between shift work and sickness absence. | 1 | R | Sickness absence |
| Bigert et al. 2022 (38) | Cohort | Healthcare workers | Sweden | To evaluate the effects of various aspects of night and shift work, regarding incident stroke and other CeVD, by using detailed and registry-based exposure data. | 1 | R | CVD and stroke |
| Blomberg et al. 2016 (121) | Cross-sectional | Registered nurses | Sweden | To investigate occupational stress among newly graduated nurses in relation to their workplace and clinical group supervision. | 4 | H | Stress |
| Cheng et al. 2021 (54) | Cohort | Healthcare workers | Finland | To examine associations between shift work and mood disorders as well as the sleep problems of workers with differing chronotypes. | 1 | R | Mood disorders, sleep problems |
| Cohidon et al. 2019 (77) | Cross-sectional | Physicians | Norway, Sweden | To use international comparisons to explore the structural and organisational factors associated with GPs’ dissatisfaction at work. | 2 | R | Dissatisfaction at work |
| Dahlgren et al. 2021(69) | Cohort | Registered nurses | Sweden | To use an intensive longitudinal design to determine whether variation in QR, both within and between individuals, was associated with self-rated stress in newly graduated nurses. | 1 | R | Stress |
| Erdem et al. 2017 (51) | Case control | Registered nurses | Norway | To investigate telomere length (TL) variation as a potential mechanism of the association between long duration of night shift with several consecutive nights and the increased risk of breast cancer. | 1 | R | Telomer shortening |
| Fallman et al. 2019 (128) | Cohort | Healthcare managers | Sweden | To investigate how restricted decision‐making autonomy and conflicting demands impact operational managers’ work performance and health. | 5 | R | Self-rated health |
| Fallman et al. 2022 (94) | Qualitative | Healthcare managers | Sweden | To identify first-line managers’ approaches for maintaining low levels of sick leave among their health care employees. | 2 | H | Sickness absence |
| Gamskjaer et al. 2022 (81) | Qualitative | Healthcare workers | Denmark | To investigate reflections and perspectives from health professionals working within palliative rehabilitation for elements of importance in relation to job satisfaction. | 2 | H | Job satisfaction |
| Golay et al. 2022 (112) | Qualitative | Registered nurses | Sweden | To provide a view of the concrete ways in which work-related IT use can compromise hospital nurses' well-being at work. | 3 | R | Anxiety, frustration |
| Golay et al. 2022 (110) | Qualitative | Registered nurses | Sweden | To understand the appraisals and emotions at the core of nurses' positive experiences with information technology use at work. | 3 | H | Happiness, trust, recovery |
| Golvani et al. 2021(106) | Qualitative | Registered nurses | Sweden | To describe operating room nurses’ experiences of limited access to daylight in the workplace. | 3 | R | Stress, exhaustion, headache |
| Grasmo et al. 2021 (63) | Qualitative | Healthcare workers | Norway | To explore the views of home care workers on how working conditions affect their safety, health, and wellbeing. | 1, 5 | R | Fatigue, accidents, pain |
| Grønstad et al. 2020 (120) | Cohort | Healthcare workers | Norway | To examine if and how the relationship between unit-level downsizing and sickness absence is moderated by three salient work factors: temporary contracts at the individual-level, and control and organisational commitment at the work-unit level. | 4 | R | Sickness absence |
| Grønstad et al. 2019 (127) | Cohort | Healthcare workers | Norway | To examine the different relationships between six unit-level changes (upsizing, downsizing, merger, spin-off, outsourcing, and insourcing) and sickness absence among hospital employees. | 5 | H | Sickness absence |
| Gyllensten et al. 2017 (138) | Qualitative | Healthcare workers | Sweden | To investigate the experiences of reduced work hours and no lunch breaks among a group of nurses and assistant nurses, with a particular focus on recovery and psychosocial working environment. | 1 | H | Energy |
| Hammer et al. 2019 (52) | Cohort | Healthcare workers | Denmark | To investigate the association of different dimensions of night work, expressed by frequency and duration of night shifts throughout pregnancy, with the risk of severe PPD. | 1 | R | Post-partum depression |
| Hansen et al. 2016 (42) | Cohort | Registered nurses | Denmark | To investigate the association between shift work and incidence of diabetes over 13 years among Danish female nurses who were members of the Danish Nurse Cohort. | 1 | R | Diabetes |
| Härmä et al. 2019 (40) | Cohort | Healthcare workers | Finland | To assess whether continuous exposure to shift work would be associated with the risk for increased fatigue and changes in sleep length over 24 h. | 1 | R | Fatigue, sleep problems |
| Härmä et al. 2018 (45) | Cohort | Healthcare workers | Finland | To examine whether changes in work shifts and shift intensity are related to changes in difficulties to fall asleep, fatigue, and sleep length. | 1 | R | Fatigue |
| Härmä et al. 2020 (66) | Cohort | Healthcare workers | Finland | To investigate the association of working hours with occupational injuries in hospital shift work. | 1 | R | Occupational injuries |
| Henriksen et al. 2016 (55) | Cross-sectional | Midwives | Norway | To assess burnout levels among Norwegian midwives and identify personal and work-related factors associated with burnout. | 1 | R | Burnout |
| Heponiemi et al. 2017 (113) | Cohort | Physicians | Finland | To examine the 9-year longitudinal development of SRIS (stress related to information systems) levels among Finnish physicians. | 3 | R | Stress |
| Heponiemi et al. 2021 (115) | Cross-sectional | Registered nurses | Finland | To examine the association between using a mobile version of electronic health records (EHR) and perceived time pressure, stress related to information systems, and self-rated stress. | 3 | R | Stress |
| Heponiemi et al. 2019 (116) | Cross-sectional | Physicians | Finland | To examine the association of usability variables (perceived benefits, technical problems, support for feedback, and user-friendliness), 2) the number of systems in daily use, (3) experience of using information systems, and (4) participation in information systems development work with physicians' distress and levels of stress related to information systems (SRIS) levels. | 3 | R | Mental ill-health, anxiety |
| Herttuala et al. 2020 (79) | Qualitative | Healthcare managers | Finland | To clarify factors that support and prevent managers’ work wellbeing by reviewing international research literature and interviewing Finnish social and healthcare managers. | 2, 4 | R | Wellbeing |
| Holmberg et al. 2016 (117) | Cross-sectional | Registered nurses | Sweden | To identify factors having positive impact on job satisfaction among Swedish psychiatric nursing staff in an inpatient psychiatric clinic. | 4 | R | Job satisfaction |
| Hult et al. 2022 (119) | Cross-sectional | Registered nurses | Finland | To explore employment precariousness, health, and work well-being among permanent and temporary nurses. | 4 | R | Health, Wellbeing |
| Jacobsen et al. 2022 (99) | Cross-sectional | Registered nurses | Denmark | To investigate whether employees in units with medium-sized spans of control observe more leadership and have higher job satisfaction, and whether this span of control can affect leadership behaviours differently. | 2 | H | Job satisfaction |
| Jakobsen et al. 2018 (124) | RCT | Healthcare workers | Denmark | To evaluate the effect of workplace versus home-based physical exercise on pressure pain threshold (PPT) and musculoskeletal pain intensity in multiple body regions. | 4 | R | Physical exercise |
| Jensen et al. 2018 (61) | Cross-sectional | Registered nurses | Denmark | To examine how intensive care nurses experience the effects of shift work on life outside work. | 1 | R | Mental ill-health |
| Jepsen et al. 2017 (87) | Cross-sectional | Midwives | Denmark | To investigate burnout among midwives – including a comparison of the level of burnout in caseloading midwives and midwives working in other models of care who do not provide continuity of care. | 2 | H | Burnout |
| Jepsen et al. 2016 (88) | Qualitative | Midwives | Denmark | To advance knowledge about the working and living conditions of midwives in caseload midwifery and how this model of care is embedded in a standard maternity unit. | 2 | H | Job satisfaction |
| Johnsen et al 2022 (84) | Cross-sectional | Physicians | Norway | To compare the workload and range of tasks between inexperienced and experienced GPs. Additionally, to addresses the extent to which clinical experience affects the way GPs perceive their daily work, including perceived levels of unhealthy stress. | 2 | R | Self-rated health |
| Jørgensen et al. 2020 (49) | Cohort | Registered nurses | Denmark | To examine the association between the type of shiftwork schedule and duration, and the incidence of dementia in the Danish nursing cohort, using detailed exposure information assessed at three different time points. | 1 | R | Dementia |
| Jørgensen et al. 2021 (50) | Cohort | Registered nurses | Denmark | To examine in detail associations between different shift work schedules (day, evening, night, and rotating) and incidence of major psychiatric disorders, including mood disorders, neurotic disorders, and substance use. | 1 | R | Mood disorders |
| Jørgensen et al. 2017 (60) | Cohort | Registered nurses | Denmark | To examine the association between shift work and all-cause mortality and mortality due to CVD, cancer, diabetes, neurodegenerative and psychiatric diseases in the Danish nurse cohort (DNC). | 1 | R | Mortality |
| Jørgensen et al. 2021 (59) | Cohort | Registered nurses | Denmark | To examine whether shift work is associated with incidence of PD, by examining the effect of different shift work schedules (day, evening, night, rotating) and whether there is a dose–response relationship between duration (cumulative years) of different shift work schedules and incidence of PD. | 1 | R | Parkinson’s disease |
| Kader et al. 2021 (48) | Cohort | Healthcare workers | Sweden | To investigate the risk of PTB (pre-term birth) in relation to detailed, registry-based data on working hours. | 1 | R | Pre-term birth |
| Kader et al. 2022 (41) | Cohort | Healthcare workers | Sweden | To examine the effects of various aspects of night and shift work on the risk of incident ischemic heart disease (IHD) and atrial fibrillation (AF) using detailed and registry-based exposure data. | 1 | R | Heart diseases |
| Kaltenbrunner et al. 2019 (89) | Cohort | Healthcare workers | Sweden | To examine the extent to which changes over time in Lean maturity are associated with changes over time in care-giving, thriving and exhaustion, as perceived by staff, with a particular emphasis on the extent to which job demands and job resources, as perceived by staff, have a moderated mediation effect. | 2 | H | Exhaustion, wellbeing |
| Karhula et al. 2018 (62) | Cross-sectional | Healthcare workers | Finland | To study sleep and psychosocial factors at work among permanent night workers by comparing them to day workers and three shift workers. | 1 | R | Sleep problems, exhaustion |
| Karhula et al. 2020 (139) | Quasi-experimental intervention | Healthcare workers | Finland | To investigate the effects of the implementation of software for participatory working time scheduling on realized working hour characteristics and changes in several wellbeing outcomes. | 1 | H | Health |
| Kjellström et al. 2017 (78) | Qualitative | Healthcare workers | Sweden | To carry out a deductive analysis of factors that influence professional work motivation on individual, organisational, and cultural level at well-functioning primary healthcare units. | 2 | H | Motivation |
| Kjørstad et al. 2022 (109) | Cross-sectional | Registered nurses | Norway | To use both work and sleep diaries and actigraphy recordings to investigate nurses’ sleep patterns, work functioning, levels of stress, and mood state over a 2-week period during which they undertook shifts in either a BDLE (blue-depleted light environment) or a STLE (standard hospital light environment). The secondary aim was to explore the nurses’ self-reported medical and mental health when working in each light environment. | 3 | R | Physical and mental ill-health |
| Larsen et al. 2020 (46) | Cross-sectional | Registered nurses | Denmark Finland | To investigate the association between timing and length of work shifts, short time between shifts (quick returns), number of consecutive nightshifts, and weekly working hours and the risk of long-term sickness absence (≥30 consecutive days) among female nursing personnel in the public healthcare sector in Denmark and Finland. | 1 | R | Sickness absence |
| Lee et al. 2021 (105) | Qualitative | Healthcare workers | Sweden | To explore staff perspectives of the physical environment in supporting their care practices for residents living with dementia in Canadian and Swedish long-term care facilities. | 3 | H | Job satisfaction |
| Lindegård et al. 2016 (103) | Cohort | Dental healthcare workers | Sweden | To investigate the effects on self-reported neck pain, clinically diagnosed conditions in the neck, perceived exertion, and self-reported work ability among dental personal opting to use prismatic glasses during clinical dental work | 3 | H | Neck pain |
| Liss et al. 2018 (123) | Cross-sectional | Dental hygienists | Sweden | To explore and analyse DHs’ self-reported views on: 1. professional competencies and behavioural interventions in periodontal therapy, 2. work-related support in the treatment of periodontitis patients and daily practice, and overall work satisfaction. | 4 | H | Job satisfaction |
| Loft and Jensen 2020 (74) | Qualitative | Registered nurses | Denmark | To explore which factors are important in terms of experienced nurses’ intention to stay in the clinical setting and to learn which factors affect their job satisfaction. | 1, 4 | H | Job satisfaction |
| Lunde et al. 2021 (90) | Cohort | Healthcare workers | Norway | To determine the association between objectively measured standing at work and lower-extremity pain intensity (LEPi) in construction and healthcare workers over a 2- year period. | 2 | R | Pain |
| Mauno et al. 2016 (126) | Cross-sectional | Registered nurses | Finland | To examine whether three resources, that is, compassion, transformational leadership, and work ethic feasibility, buffer against the negative effects of emotional labour on work engagement. | 5 | H | Engagement |
| Møller et al. 2022 (68) | Cross-sectional | Physicians | Denmark | To identify the prevalence of burnout among VSs (vascular surgeons) and VSTs in Denmark and identify potential burnout risk factors, including psychosocial working conditions. | 1 | R | Burnout |
| Nielsen et al. 2019 (58) | Cohort | Healthcare workers | Denmark | To assess how duration of time between shifts – and, specifically, quick returns – affects risk of injury. Additionally, we evaluated the association between injury and days since a quick return as well as the number of quick returns in the past week. | 1 | R | Injury |
| Nielsen et al. 2019 (57) | Case control | Healthcare workers | Denmark | To assess how shift work characteristics affect the risk of occupational, transport, and leisure-time injuries. | 1 | R | Injury |
| Nielsen et al. 2016 (92) | Qualitative | Healthcare workers | Denmark | To extend the current knowledge of employee engagement by emphasising how caregivers experience meaning in their work. | 2, 4 | R | Engagement |
| Olsen et al. 2017 (91) | Cross-sectional | Registered nurses | Norway | To explore the influence of job resources and job demands on bullying and three self-reported nurse outcomes. The selected outcome variables were job performance, job satisfaction, and work ability. | 2 | R | Jo satisfaction work ability |
| Ose et al. 2022 (125) | Cross-sectional | Registered nurses | Norway | To identify the causes of work-related sick leave among Norwegian hospital nurses. | 4 | R | Sickness absence |
| Ose et al. 2019 (67) | Qualitative | Registered nurses | Norway | To perform a thorough qualitative study, to understand nurses' experiences and perceptions of working 12-hour shifts compared with the usual 8-hour shifts. | 1 | R | Health |
| Pedersen et al. 2020 (93) | Randomized field experiment | Physicians | Denmark | To investigate whether accreditation has a negative effect on GP (general practitioner) job satisfaction. | 2 | R | Job satisfaction |
| Persson et al. 2018 (53) | Cross-sectional | Healthcare workers | Sweden | To examine the association between workplace relationships, with a focus on colleague belongingness, and self-rated health among employees in a Swedish municipal elderly health care organisation. | 1 | R | Health |
| Poikkeus et al. 2020 (129) | Cross-sectional | Registered nurses | Finland | To examine relationships between nurses’ perceived organisational and individual support, ethical competence, ethical safety, and work satisfaction. | 5 | H | Job satisfaction |
| Rantanen et al. 2016 (85) | Cross-sectional | Registered nurses | Finland | To test the differences between the primary nursing model and the individual patient allocation model in: (1) work-related motivational characteristics; (2) work-related stress factors; and (3) job satisfaction, as reported by nurses working at one university hospital. | 2 | R | Stress |
| Riisgaard et al. 2017 (86) | Cross-sectional | Healthcare workers | Denmark | To investigate associations between degrees of task delegation and job satisfaction of GPs and their staff in Danish general practice using the management of patients with chronic obstructive pulmonary disease (COPD) as our case. | 2 | H | Job satisfaction |
| Ropponen et al. 2023 (65) | Cohort | Healthcare workers | Finland | To explore and identify working hour patterns among hospital employees working irregular working hours and to investigate the associations between the identified patterns and the risk of occupational accidents | 1 | R | Occupational accidents |
| Ropponen et al. 2022 (64) | Cohort | Healthcare workers | Finland | To investigate the association of the characteristics of working hours with the risk of short (1–3 days) sickness absence among hospital physicians. | 1 | R | Sickness absence |
| Ropponen et al. 2020 (56) | Cohort | Healthcare workers | Finland | To investigate age group differences in objective working-hour characteristics among women in hospital work and, second, the associations of working-hour characteristics with short (1–3 days) sickness absence in different age groups. | 1 | R | Sickness absence |
| Ropponen et al. 2019 (47) | Case control | Healthcare workers | Finland | To investigate the association between working-hour characteristics in shiftwork and the incidence of short (1–3 days) sickness absence among hospital employees. | 1 | R | Sickness absence |
| Rosenström et al. 2021 (118) | Cohort | Healthcare workers | Finland | To: (i) characterize working hour patterns in shift work by means of permutation distribution clustering as a data-mining tool, and (ii) study associations between these shift work patterns and sickness absence. | 4 | R | Sickness absence |
| Routsalainen et al. 2023 (80) | Cross-sectional | Healthcare workers | Finland | To examine the association between self-organizing teamwork practices and job satisfaction and turnover intentions and to examine whether psychosocial factors acted as potential mediators. | 2 | H | Job satisfaction |
| Routsalainen et al. 2020 (75) | Mixed method | Healthcare workers | Finland | To explore the challenges, stressors, teamwork and management factors that are associated with home care staff members’ well-being, job satisfaction, and experienced care quality, and further, how staff members experience their work. | 2 | R | Mental ill-health, anxiety |
| Seitovirta et al. 2017 (73) | Qualitative | Registered nurses | Finland | To identify the meaningful types of rewards and the possible consequences of rewards as expressed by RNs (registered nurses). | 1, 4 | H | Job satisfaction |
| Sigursteinsdóttir et al. 2020 (100) | Cross-sectional | Healthcare managers | Iceland | To analyse the correlation between musculoskeletal pain/discomfort in the neck and neck area, the shoulder and shoulder area, and the lower back; stressful factors in the work environment, and adequate sleep among Icelandic nursing unit managers. | 2 | R | Sleep problems |
| Slåtten et al. 2022 (96) | Cross-sectional | Registered nurses | Norway | To examine whether work engagement (WE) is a significant predictor of the achievement of certain preferred organisational goals: lowering nursing professionals’ turnover intentions and increasing job satisfaction; and increasing the quality of care provided to patients. | 2 | H | Job satisfaction, engagement |
| Spännargård et al. 2022 (122) | Cross-sectional | Psychotherapists | Sweden | To map the level of work-related burnout and fatigue among psychotherapists working in clinical settings and to investigate the relation between burnout and (a) person-related factors such as age, training, level of education, years in profession, and perceived competence; and (b) work-related factors such as type of clinical setting, satisfaction with the work situation, and access to clinical supervision | 4 | H | Burnout |
| Stadin et al. 2020 (111) | Qualitative | Healthcare managers | Sweden | To describe healthcare managers’ experience of technostress and their actions for handling it. | 3 | H | Stress |
| Svedahl et al. 2019 (83) | Qualitative | Physicians | Norway | To explore how GPs and their co-workers in Norway perceive and tackle their workload, and their experiences and reflections regarding explanations for and consequences of increased workload in general practice. | 2 | R | Health, motivation |
| Thapa et al. 2021 (98) | Qualitative | Registered nurses Midwives | Sweden | To explore and gain a deeper understanding of how nurses and midwives experience their everyday work, with a view toward promoting and sustaining their work-related health. | 2, 5 | H | Job satisfaction |
| Thun et al. 2018 (82) | Cross-sectional | Physicians | Norway | To describe the relationship between unreasonable illegitimate tasks and sickness presenteeism in physicians after controlling for variance in age, gender, role conflict, control over work pace, exhaustion and administrative tasks. | 2 | R | Sickness absence |
| Vainomäki et al. 2020 (114) | Cross-sectional | Physicians | Finland | To examine the associations of HER (electronic health record)-related variables with time pressure and stress and how these associations differed according to working environment. | 3 | R | Stress |
| Vedaa et al. 2019 (44) | Cross-sectional | Registered nurses | Norway | To examine the association between quick returns (<11h) and night shifts, and self-reported work-related accidents, near accidents, or dozing off at work. | 1 | R | Occupational accidents |
| Vedaa et al. 2017 (140) | Cohort | Registered nurses | Norway | To further examine the specific sleep-related consequences associated with QRs (quick returns), as compared with other common shift transitions. | 1 | R | Sleep problems, stress |
| Vedaa et al. 2017 (70) | Cohort | Registered nurses | Norway | To investigate whether exposure to quick returns and night shifts can predict later sick leave, and to what extent personality traits associated with shift work tolerance predict sick leave and/or moderate any such prediction by shift schedule characteristics. | 1 | R | Sickness absence |
| Vedaa et al. 2020 (72) | Cohort | Registered nurses | Norway | To investigate how a reduction or an increase in the number of QR over time are associated with the risk of nurses reporting occupational accidents. | 1 | R | Occupational accidents |
| Vifladt et al. 2016 (97) | Cross-sectional | Registered nurses | Norway | To investigate associations between the RNs’ (registered nurses) perception of the safety culture in ICUs, and burnout and sense of coherence. The secondary objective was to compare the scores for burnout and sense of coherence among the RNs in restructured and not restructured ICUs. | 2 | R | Burnout |
| Vilén et al. 2022 (107) | Cross-sectional | Healthcare workers | Finland | To determine the current prevalence of hoarseness among six subgroups of nurses (registered nurses, primary care nurses, paediatric nurses, laboratory nurses, dental nurses, and midwifes) and also to identify potential environmental risk factors in their working environment. | 3 | R | Hoarseness |
| Vilén et al. 2021 (108) | Cross-sectional | Healthcare workers | Finland | To determine the current prevalence of hoarseness among the nurses in six different occupational subgroups (registered nurses, primary care nurses, paediatric nurses, laboratory nurses, dental nurses, and mid-wives) in order to determine whether different occupational subgroups have different environmental risk factors for hoarseness. | 3 | R | Hoarseness |
| Vinstrup et al. 2020 (102) | Cohort | Healthcare workers | Denmark | To create an exposure-matrix to identify associations between biomechanical load during patient transfer and the odds of back injury and LBP among healthcare workers. | 3 | H | Back injury and pain |
| Waage et al. 2021 (39) | Cohort | Registered nurses | Norway | To explore how changes in the work schedule would affect the prevalence of SWD (shift work disease) over time. | 1 | H | Shift work disease |
| Westergren et al. 2022 (76) | Qualitative | Registered nurses | Sweden | To carry out an exploratory analysis of the work situation of haemodialysis nurses from an ergonomic perspective. | 3 | R | Musculoskeletal symptoms |
| Westergren et al. 2020 (104) | Cross-sectional | Registered nurses | Sweden | (1) To examine the association between the type of dialysis machine and disposables used with the occurrence of hand complaints among haemodialysis nurses and (2) to compare occupational risks [revised strain index (RSI)] of developing work‐related MSDs of the distal upper extremities based on the materials used for haemodialysis. | 3 | R | Musculoskeletal symptoms |
| ^a^1= work schedule distribution, 2=operations design and work methods, 3=ergonomic conditions, 4= terms of employment and personnel policies, and 5=the organisation’s ethical environment, ^b^R=risk factor, H=health-promoting factor | | | | | | | |
