# Supplementary material for: Organisational-level risk and health-promoting factors within the healthcare sector—a systematic search and review
Source: Front Med (Lausanne). 2025 Jan 17;11:1509023. doi: 10.3389/fmed.2024.1509023 (PMC11783186; doi:10.3389/fmed.2024.1509023)
Supplement: Supplementary file 3 [file Data_Sheet_2.PDF]

S2 Search terms

If all or a substantive part of the search strategies below are reused in another publication, please cite this report!

Cinahl med fulltext via EBSCO 2023-01-03

Title: Kunskapssammanställning om arbetsmiljörisker och friskfaktorer bland hälso- och sjukvårdspersonal_version1

	Search terms	Items found
**Population: Health personnel**		
1.	(MH "Health Occupations+") OR (MH "Health Personnel+")	1 275 025
2.	TI ("health personnel" OR "hospital personnel" OR "health occupation*" OR "nursing staff*" OR physician* OR doctor* OR nurse* OR "medical staff*" OR "health profession*" OR "health care provider*" OR "healthcare provider*" OR "health care worker*" OR "healthcare worker*" OR "health worker*" OR "health workforce*" OR "health care professional*" OR "healthcare professional*" OR "healthcare staff*" OR "health care staff*" OR "healthcare employee*" OR "health care employee*" OR "hospital worker*" OR "hospital staff*" OR "hospital employee*" OR "dental auxiliar*" OR "dental assistant*" OR "dental hygienist*" OR "dental technician*" OR denturist* OR "emergency medical technician*" OR "home health aide*" OR "medical record administrator*" OR "medical secretar*" OR "medical receptionist*" OR "nursing assistant*" OR "psychiatric aide*" OR "operating room technician*" OR "pharmacy technician*" OR "physical therapist assistant*" OR "physician assistant*" OR "ophthalmic assistant*" OR "pediatric assistant*" OR anatomist* OR anesthetist* OR anesthesiologist* OR audiologist* OR caregiver* OR "case manager*" OR "coroners and medical examiner*" OR "dental staff*" OR dentist* OR endodontist* OR "oral and maxillofacial surgeon*" OR orthodontist* OR doula* OR "emergency medical dispatcher*" OR epidemiologist* OR "health educator*" OR "health facility administrator*" OR "hospital administrator*" OR "infection control practitioner*" OR "medical chaperone*" OR "medical laboratory personnel" OR nutritionist* OR "occupational therapist*" OR optometrist* OR "hospital administrator*" OR pharmacist* OR "physical therapist*" OR "physician executive*" OR allergist* OR anesthesiologist* OR cardiologist* OR dermatologist* OR endocrinologist* OR "foreign medical graduate*" OR gastroenterologist* OR "general practitioner*" OR geriatrician* OR hospitalist* OR nephrologist* OR neurologist* OR oncologist* OR ophthalmologist* OR otolaryngologist* OR pathologist* OR pediatrician* OR neonatologist* OR physiatrist* OR pulmonologist* OR radiologist* OR rheumatologist* OR surgeon* OR neurosurgeon* OR urologist* OR psychotherapist* OR midwife* OR midwives)	398 708
3.	AB ("health personnel" OR "hospital personnel" OR "health occupation*" OR "nursing staff*" OR physician* OR doctor* OR nurse* OR "medical staff*" OR "health profession*" OR "health care provider*" OR "healthcare provider*" OR "health care worker*" OR "healthcare worker*" OR "health worker*" OR "health workforce*" OR "health care professional*" OR "healthcare professional*" OR "healthcare staff*" OR "health care staff*" OR "healthcare employee*" OR "health care employee*" OR "hospital worker*" OR "hospital staff*" OR "hospital employee*" OR "dental auxiliar*" OR "dental assistant*" OR "dental hygienist*" OR "dental technician*" OR denturist* OR "emergency medical technician*" OR "home health aide*" OR "medical record administrator*" OR "medical secretar*" OR "medical receptionist*" OR "nursing assistant*" OR "psychiatric aide*" OR "operating room technician*" OR "pharmacy technician*" OR "physical therapist assistant*" OR "physician assistant*" OR "ophthalmic assistant*" OR "pediatric assistant*" OR anatomist* OR anesthetist* OR anesthesiologist* OR audiologist* OR caregiver* OR "case manager*" OR "coroners and medical examiner*" OR "dental staff*" OR dentist* OR endodontist* OR "oral and maxillofacial surgeon*" OR orthodontist* OR doula* OR "emergency medical dispatcher*" OR epidemiologist* OR "health educator*" OR "health facility administrator*" OR "hospital administrator*" OR "infection control practitioner*" OR "medical chaperone*" OR "medical laboratory personnel" OR nutritionist* OR "occupational therapist*" OR optometrist* OR "hospital administrator*" OR	776 133

	pharmacist* OR "physical therapist*" OR "physician executive*" OR allergist* OR anesthesiologist* OR cardiologist* OR dermatologist* OR endocrinologist* OR "foreign medical graduate*" OR gastroenterologist* OR "general practitioner*" OR geriatrician* OR hospitalist* OR nephrologist* OR neurologist* OR oncologist* OR ophthalmologist* OR otolaryngologist* OR pathologist* OR pediatrician* OR neonatologist* OR physiatrist* OR pulmonologist* OR radiologist* OR rheumatologist* OR surgeon* OR neurosurgeon* OR urologist* OR psychotherapist* OR midwife* OR midwives)	
4.	1 OR 2 OR 3	1 848 937
**Exposure: Occupational health**		
5.	(MH "Occupational Health") OR (MH "Work Environment") OR (MH "Quality of Working Life") OR (MH "Job Satisfaction")	84 028
6.	TI ("occupational health*" OR "occupational safet*" OR "personnel* health*" OR "employee* health*" OR "worker* health*" OR "workplace health*" OR "worksite health*" OR "staff health*" OR "work* environment*" OR "occupational environment*" OR "work* culture*" OR "work* relat*" OR “job related” OR "work* condition*" OR "work* climat*" OR "organizational climate*" OR "organisational climate*" OR "work atmosphere*" OR "job satisfaction*" OR "work* satisfaction*" OR "employee* satisfaction*" OR "quality of work* life" OR "occupational exposure*" OR "work* motivation*" OR "job motivation*" OR "employee* motivation*")	18 039
7.	AB ("occupational health*" OR "occupational safet*" OR "personnel* health*" OR "employee* health*" OR "worker* health*" OR "workplace health*" OR "worksite health*" OR "staff health*" OR "work* environment*" OR "occupational environment*" OR "work* culture*" OR "work* relat*" OR “job related” OR "work* condition*" OR "work* climat*" OR "organizational climate*" OR "organisational climate*" OR "work atmosphere*" OR "job satisfaction*" OR "work* satisfaction*" OR "employee* satisfaction*" OR "quality of work* life" OR "occupational exposure*" OR "work* motivation*" OR "job motivation*" OR "employee* motivation*")	48 590
8.	5 OR 6 OR 7	117 265
**Context: Geographic**		
9.	(MH "Scandinavia") OR (MH "Denmark") OR (MH "Finland") OR (MH "Norway") OR (MH "Sweden") OR (MH "Greenland") OR (MH "Iceland")	81 697
10.	TI (denmark OR danish OR “faroe island*” OR finland OR finnish OR finns OR greenland* OR iceland* OR norway OR norwegian* OR swed* OR åland* OR nordic* OR scandinavia*)	32 289
11.	AB (denmark OR danish OR “faroe island*” OR finland OR finnish OR finns OR greenland* OR iceland* OR norway OR norwegian* OR swed* OR åland* OR nordic* OR scandinavia*)	65 348
12.	9 OR 10 OR 11	107645
**Combined sets**		
13.	4 AND 8 AND 12	2045
**Limits**		
14.	Publication year: 2016/01/01 -	843
15.	Language: Danish, English, Norwegian, Swedish	827
**Final result**		
**16.**	13 AND 14 AND 15	**827**

[MH] = Exact Subject heading; **[MH+] =** Exact Subject heading Explode (The headings are exploded to retrieve all references indexed to that term as well as all references indexed to any narrower subject terms.)

[AB] = Term from abstract; **[TI]** = Term from title; **[" "]** = Citation Marks; searches for an exact phrase; **[*]** = Truncation

PsycInfo via Proquest 2023-01-03

Title: Kunskapssammanställning om arbetsmiljörisker och friskfaktorer bland hälso- och sjukvårdspersonal_version1

	Search terms	Items found
**Population: Health Personnel**		
•	MAINSUBJECT.EXACT.EXPLODE("Health Personnel") OR MAINSUBJECT.EXACT("Clinicians")	196 139
•	noft("health personnel" OR "hospital personnel" OR "health occupation*" OR "nursing staff*" OR physician* OR doctor* OR nurse* OR midwife* OR midwives OR "medical staff*" OR "health profession*" OR "health care provider*" OR "healthcare provider*" OR "health care worker*" OR "healthcare worker*" OR "health worker*" OR “health workforce*” OR "health care professional*" OR "healthcare professional*" OR "healthcare staff*" OR "health care staff*" OR “healthcare employee*” OR “health care employee*” OR "hospital worker*" OR "hospital staff*" OR "hospital employee*")	380 497
•	1 OR 2	453 354
**Exposure: Occupational Health**		
•	MAINSUBJECT.EXACT.EXPLODE("Occupational Health") OR MAINSUBJECT.EXACT.EXPLODE("Working Conditions") OR MAINSUBJECT.EXACT("Organizational Climate") OR MAINSUBJECT.EXACT("Occupational Exposure") OR MAINSUBJECT.EXACT("Job Satisfaction") OR MAINSUBJECT.EXACT("Employee Motivation") OR MAINSUBJECT.EXACT("Quality of Work Life")	74 075
•	noft("occupational health*" OR "occupational safet*" OR "personnel* health*" OR "employee* health*" OR "worker* health*" OR "workplace health*" OR "worksite health*" OR "staff health*" OR "work* environment*" OR "occupational environment*" OR "work* culture*" OR "work* relat*" OR “job related” OR "work* condition*" OR "work* climat*" OR "organi?ational climate*" OR "work atmosphere*" OR "job satisfaction*" OR "work* satisfaction*" OR "employee* satisfaction*" OR "quality of work* life" OR "occupational exposure*" OR "work* motivation*" OR "job motivation*" OR "employee* motivation*")	127 575
•	4 OR 5	129 834
**Context: Geographic**		
•	noft(denmark OR danish OR “faroe island*” OR finland OR finnish OR finns OR greenland* OR iceland* OR norway OR norwegian* OR swed* OR åland* OR nordic* OR Scandinavia*)	144 562
**Combined sets**		
•	3 AND 6 AND 7	1 615
**Limits**		
•	Publikation year: 2016/01/01 -	530
•	Language: Danish, English, Norwegian, Swedish	529
**Final result**		
•	**8 AND 9 AND 10**	529

[MAINSUBJECT.EXACT] =Term from the PsycInfo thesaurus; **[MAINSUBJECT.EXACT.EXPLODE] =** Includes terms found below this term in the PsycInfo thesaurus; **[noft]** = Anywhere except full text; **[" "]** = Citation Marks; searches for an exact phrase; **[*]** = Truncation

Pubmed via NLM 4 2023-01-03

Title: Kunskapssammanställning om arbetsmiljörisker och friskfaktorer bland hälso- och sjukvårdspersonal_version1

	Search terms	Items found
**Population: Health Personnel**		
•	"Health Personnel"[Mesh] OR "Health Occupations"[Mesh]	2272423
•	"health personnel"[Title/Abstract] OR "hospital personnel"[Title/Abstract] OR "health occupation*"[Title/Abstract] OR "nursing staff*"[Title/Abstract] OR physician*[Title/Abstract] OR doctor*[Title/Abstract] OR nurse*[Title/Abstract] OR "medical staff*"[Title/Abstract] OR "health profession*"[Title/Abstract] OR "health care provider*"[Title/Abstract] OR "healthcare provider*"[Title/Abstract] OR "health care worker*"[Title/Abstract] OR "healthcare worker*"[Title/Abstract] OR "health care professional*"[Title/Abstract] OR "healthcare professional*"[Title/Abstract] OR "healthcare staff*"[Title/Abstract] OR "health care staff*"[Title/Abstract] OR "healthcare employee*"[Title/Abstract] OR "health care employee*"[Title/Abstract] OR "hospital worker*"[Title/Abstract] OR "hospital staff*"[Title/Abstract] OR "hospital employee*"[Title/Abstract] OR "community health worker*"[Title/Abstract] OR "dental auxiliar*"[Title/Abstract] OR "dental assistant*"[Title/Abstract] OR "dental hygienist*"[Title/Abstract] OR "dental technician*"[Title/Abstract] OR denturist*[Title/Abstract] OR "emergency medical technician*"[Title/Abstract] OR "home health aide*"[Title/Abstract] OR "medical record administrator*"[Title/Abstract] OR "medical secretar*"[Title/Abstract] OR "medical receptionist*"[Title/Abstract] OR "nursing assistant*"[Title/Abstract] OR "psychiatric aide*"[Title/Abstract] OR "operating room technician*"[Title/Abstract] OR "pharmacy technician*"[Title/Abstract] OR "physical therapist assistant*"[Title/Abstract] OR "physician assistant*"[Title/Abstract] OR "ophthalmic assistant*"[Title/Abstract] OR "pediatric assistant*"[Title/Abstract] OR anatomist*[Title/Abstract] OR anesthetist*[Title/Abstract] OR anesthesiologist*[Title/Abstract] OR audiologist*[Title/Abstract] OR caregiver*[Title/Abstract] OR "case manager*"[Title/Abstract] OR "coroners and medical examiner*"[Title/Abstract] OR "dental staff*"[Title/Abstract] OR dentist*[Title/Abstract] OR endodontist*[Title/Abstract] OR "oral and maxillofacial surgeon*"[Title/Abstract] OR orthodontist*[Title/Abstract] OR doula*[Title/Abstract] OR "emergency medical dispatcher*"[Title/Abstract] OR epidemiologist*[Title/Abstract] OR "health educator*"[Title/Abstract] OR "health facility administrator*"[Title/Abstract] OR "hospital administrator*"[Title/Abstract] OR "infection control practitioner*"[Title/Abstract] OR "medical chaperone*"[Title/Abstract] OR "medical laboratory personnel"[Title/Abstract] OR nutritionist*[Title/Abstract] OR "occupational therapist*"[Title/Abstract] OR optometrist*[Title/Abstract] OR pharmacist*[Title/Abstract] OR "physical therapist*"[Title/Abstract] OR "physician executive*"[Title/Abstract] OR allergist*[Title/Abstract] OR cardiologist*[Title/Abstract] OR dermatologist*[Title/Abstract] OR endocrinologist*[Title/Abstract] OR "foreign medical graduate*"[Title/Abstract] OR gastroenterologist*[Title/Abstract] OR "general practitioner*"[Title/Abstract] OR geriatrician*[Title/Abstract] OR hospitalist*[Title/Abstract] OR nephrologist*[Title/Abstract] OR neurologist*[Title/Abstract] OR oncologist*[Title/Abstract] OR ophthalmologist*[Title/Abstract] OR otolaryngologist*[Title/Abstract] OR pathologist*[Title/Abstract] OR pediatrician*[Title/Abstract] OR neonatologist*[Title/Abstract] OR physiatrist*[Title/Abstract] OR pulmonologist*[Title/Abstract] OR radiologist*[Title/Abstract] OR rheumatologist*[Title/Abstract] OR surgeon*[Title/Abstract] OR neurosurgeon*[Title/Abstract] OR urologist*[Title/Abstract] OR psychotherapist*[Title/Abstract] OR "health worker*"[Title/Abstract] OR "health workforce*"[Title/Abstract] OR midwife*[Title/Abstract] OR midwives[Title/Abstract]	1764884
•	1 OR 2	3402383
**Exposure: Occupational Health**		
•	"Occupational Health"[Mesh] OR "Job Satisfaction"[Mesh] OR "Occupational Exposure"[Mesh]	127 175
•	"occupational health*"[Title/Abstract] OR "occupational safet*"[Title/Abstract] OR "personnel health*"[Title/Abstract] OR "personnels health*"[Title/Abstract] OR "employee health*"[Title/Abstract] OR "employees health*"[Title/Abstract] OR "worker health*"OR "workers health*"[Title/Abstract] OR "workplace health*"[Title/Abstract] OR "worksite health*"[Title/Abstract] OR "staff health*"[Title/Abstract] OR "work environment*"[Title/Abstract] OR "working environment*"[Title/Abstract] OR "occupational environment*"[Title/Abstract] OR	112 090

	"work culture*"[Title/Abstract] OR "working culture*"[Title/Abstract] OR "work relat*"[Title/Abstract] OR "workplace relat*"[Title/Abstract] OR "job relat*"[Title/Abstract] OR "work condition*"[Title/Abstract] OR "working condition*"[Title/Abstract] OR "work climat*"[Title/Abstract] OR "workers climat*"[Title/Abstract] OR "working climat*"[Title/Abstract] OR "organizational climate*"[Title/Abstract] OR "organisational climate*"[Title/Abstract] OR "work atmosphere*"[Title/Abstract] OR "job satisfaction*"[Title/Abstract] OR "work satisfaction*"[Title/Abstract] OR "workers satisfaction*"[Title/Abstract] OR "working satisfaction*"[Title/Abstract] OR "employee satisfaction*"[Title/Abstract] OR "employees satisfaction*"[Title/Abstract] OR "quality of work life"[Title/Abstract] OR "quality of working life"[Title/Abstract] OR "occupational exposure*"[Title/Abstract] OR "work motivation*"[Title/Abstract] OR "workers motivation*"[Title/Abstract] OR "working motivation*"[Title/Abstract] OR "job motivation*"[Title/Abstract] OR "employee motivation*"[Title/Abstract] OR "employees motivation*"[Title/Abstract]	
•	4 OR 5	198 953
**Context: Geographic**		
•	"Scandinavian and Nordic Countries"[Mesh]	218793
•	denmark[Text Word] OR danish[Text Word] OR "faroe island*"[Text Word] OR finland[Text Word] OR finnish[Text Word] OR finns[Text Word] OR greenland*[Text Word] OR iceland*[Text Word] OR norway[Text Word] OR norwegian*[Text Word] OR swed*[Text Word] OR åland*[Text Word] OR nordic*[Text Word] OR scandinavia*[Text Word]	323810
•	7 OR 8	324166
**Combined sets**		
•	3 AND 6 AND 9	3356
**Limits**		
•	Publikation year: 2016/01/01 -	1019
•	Language: Danish, English, Norwegian, Swedish	**1009**
**Final result**		
•	**10 AND 11 AND 12**	1009

[MeSH] = Term from the Medline controlled vocabulary, including terms found below this term in the MeSH hierarchy; **[MeSH:NoExp]** = Does not include terms found below this term in the MeSH hierarchy; **[TIAB]** = Title or abstract; **[" "]** = Citation Marks; searches for an exact phrase; **[*]** = Truncation **[Text Word]** = Includes all words and numbers in the title, abstract, other abstract, MeSH terms, MeSH Subheadings, Publication Types, Substance Names, Personal Name as Subject, Corporate Author, Secondary Source, Comment/Correction Notes, and Other Terms

Scopus 2023-01-03

Title: Kunskapssammanställning om arbetsmiljörisker och friskfaktorer bland hälso- och sjukvårdspersonal_version1

	Search terms	Items found
**Population: Health personnel**		
•	TITLE-ABS-KEY ("health personnel" OR “hospital personnel” OR "health occupation*" OR "nursing staff*" OR physician* OR doctor* OR nurse* OR "medical staff*" OR "health profession*" OR "health care provider*" OR "healthcare provider*" OR "health care worker*" OR "healthcare worker*" OR "health worker*" OR “health workforce*” OR "health care professional*" OR "healthcare professional*" OR "healthcare staff*" OR "health care staff*" OR “healthcare employee*” OR “health care employee*” OR "hospital worker*" OR "hospital staff*" OR "hospital employee*" OR "dental auxiliar*" OR "dental assistant*" OR "dental hygienist*" OR "dental technician*" OR denturist* OR "emergency medical technician*" OR "home health aide*" OR "medical record administrator*" OR "medical secretar*" OR "medical receptionist*" OR "nursing assistant*" OR "psychiatric aide*" OR "operating room technician*" OR "pharmacy technician*" OR "physical therapist assistant*" OR "physician assistant*" OR "ophthalmic assistant*" OR "pediatric assistant*" OR anatomist* OR anesthetist* OR anesthesiologist* OR audiologist* OR caregiver* OR "case manager*" OR "coroners and medical examiner*" OR "dental staff*" OR dentist* OR endodontist* OR "oral and maxillofacial surgeon*" OR orthodontist* OR doula* OR "emergency medical dispatcher*" OR epidemiologist* OR "health educator*" OR "health facility administrator*" OR "hospital administrator*" OR "infection control practitioner*" OR "medical chaperone*" OR "medical laboratory personnel" OR nutritionist* OR "occupational therapist*" OR optometrist* OR "hospital administrator*" OR pharmacist* OR "physical therapist*" OR "physician executive*" OR allergist* OR anesthesiologist* OR cardiologist* OR dermatologist* OR endocrinologist* OR "foreign medical graduate*" OR gastroenterologist* OR "general practitioner*" OR geriatrician* OR hospitalist* OR nephrologist* OR neurologist* OR oncologist* OR ophthalmologist* OR otolaryngologist* OR pathologist* OR pediatrician* OR neonatologist* OR physiatrist* OR pulmonologist* OR radiologist* OR rheumatologist* OR surgeon* OR neurosurgeon* OR urologist* OR psychotherapist* OR midwife* OR midwives)	3 064888
**Exposure: Occupational health**		
•	TITLE-ABS-KEY ("occupational health*" OR "occupational safet*" OR "personnel* health*" OR "employee* health*" OR "worker* health*" OR "workplace health*" OR "worksite health*" OR "staff health*" OR "work* environment*" OR "occupational environment*" OR "work* culture*" OR "work* relat*" OR “job relat*” OR "work* condition*" OR "work* climat*" OR "organi?ational climate*" OR "work atmosphere*" OR "job satisfaction*" OR "work* satisfaction*" OR "employee* satisfaction*" OR "quality of work* life" OR "occupational exposure*" OR "work* motivation*" OR "job motivation*" OR "employee* motivation*")	456 859
**Context: Countries**		
•	TITLE-ABS-KEY(denmark OR danish OR "faroe island*" OR finland OR finnish OR finns OR greenland* OR iceland* OR norway OR norwegian* OR swed* OR åland* OR nordic* OR scandinavia*)	720 897
4.	AFFILCOUNTRY(denmark OR danish OR "faroe island*" OR finland OR finnish OR finns OR greenland* OR iceland* OR norway OR norwegian* OR swed* OR åland* OR nordic* OR scandinavia*)	2 674 773
5.	3 OR 4	2 941 071
**Combined sets**		
6.	1 AND 2 AND 5	5693
**Limits**		
7.	Publication year: 2016/01/01 -	2110
8.	Language: Danish, English, Norwegian, Swedish	2096
**Final result**		
**9.**	**6 AND 7 AND 8**	2096

[TITLE-ABS-KEY] = Includes terms from the title, abstract and keywords
[AFFILCOUNTRY] = Affiliation Country, from the author affiliation fields
[" "] = Citation Marks; searches for an exact phrase; **[*]** = Truncation
