# Supplementary material for: Organisational-level risk and health-promoting factors within the healthcare sector—a systematic search and review
Source: Front Med (Lausanne). 2025 Jan 17;11:1509023. doi: 10.3389/fmed.2024.1509023 (PMC11783186; doi:10.3389/fmed.2024.1509023)
Supplement: Supplementary file 4 [file Data_Sheet_3.PDF]

Covidence #	Study ID	Title	Reference	Country in which the study was conducted
2683	Rantanen 2016	Two models of nursing practice: A comparative study of motivational characteristics, work satisfactio	Rantanen 2016	Finland
2658	Vifladt 2016	The association between patient safety culture and burnout and sense of coherence: A cross-sectiona	Vifladt et al 2016	Norway
2624	Seitovirta 2017	Attention to nurses' rewarding—An interview study of registered nurses working in primary and priva	Seitovirta et al 2017	Finland
2593	Olsen 2017	Work climate and the mediating role of workplace bullying related to job performance, job satisfactio	Olsen et al 2017	Norway
2574	Persson 2018	Workplace relationships impact self-rated health: A survey of Swedish municipal health care employe	Persson et al 2018	Sweden
2569	Härmä 2018	Association of changes in work shifts and shift intensity with change in fatigue and disturbed sleep: A	Härmä, 2018	Finland
2566	Beltagy 2018	Night work and risk of common mental disorders: Analyzing observational data as a non-randomized	Beltagy MS et al 2018	Finland
2556	Jensen 2018	The impact of shift work on intensive care nurses' lives outside work: A cross-sectional study	Jensen 2018	Denmark
2526	Karhula 2018	Permanent night workers´ sleep and psychosocial factors in hospital work. A comparison to day and s	Karhula, 2018	Finland
2492	Ahlstedt 2019	What makes registered nurses remain in work? An ethnographic study	Ahlstedt C et al 2019	Sweden
2473	Ropponen 2019	Exposure to working-hour characteristics and short sickness absence in hospital workers: A case-cross	Ropponen et al 2019	Finland
2465	Fallman 2019	The impact of restricted decision-making autonomy on health care managers' health and work perfor	Fallman SL et al 2018	Sweden
2463	Härmä 2019	Shift work with and without night work as a risk factor for fatigue and changes in sleep length: A coho	Härmä, 2019	Finland
2427	Hammer 2019	Night work and postpartum depression: A national register-based cohort study	Hammer et al 2019	Denmark
2421	Nielsen 2019	Shift work and risk of occupational, transport and leisure-time injury. A register-based case-crossover	Nielsen et al 2019	Denmark
2404	Herttuala 2020	Social- and healthcare managers' work wellbeing—Literature review and key informant interviews	Herttuala, Kokkinen & Konu 2020	Finland
2382	Ropponen 2020	A case-crossover study of age group differences in objective working-hour characteristics and short si	Ropponen, 2019	Finland
2365	Jørgensen 2020	Shift work and incidence of dementia: A Danish nurse cohort study	Jørgensen et al 2020	Denmark
2361	Loft 2020	What makes experienced nurses stay in their position? A qualitative interview study	Loft, 2020	Denmark
2353	Vedaa 2020	A longitudinal study on the association between quick returns and occupational accidents	Vedaa et al 2020	Norway
2352	Härmä 2020	Characteristics of working hours and the risk of occupational injuries among hospital employees: A ca	Härmä, 2020	Finland
2345	Larsen 2020	Working time characteristics and long-term sickness absence among Danish and Finnish nurses: A reg	Larsen et al 2020	Other:
2344	Karhula 2020	The effects of using participatory working time scheduling software on working hour characteristics a	Karhula, 2020	Finland
2309	Cheng 2021	Association of shift work with mood disorders and sleep problems according to chronotype: A 17-yea	Cheng W et al 2021	Finland
2297	Waage 2021	Changes in work schedule affect the prevalence of shift work disorder among Norwegian nurses—A tv	Waage et al 2021	Norway
2294	Jørgensen 2021	Shift work and incidence of psychiatric disorders: The Danish Nurse Cohort study	Jörgensen, 2021	Denmark
2282	Dahlgren 2021	Intensive longitudinal study of newly graduated nurses' quick returns and self-rated stress	Dahlgren A, Tucker P, Bujacz A, Frögeli E, Rudman A, Gustavsson P, 2021	Sweden
2281	Rosenström 2021	Patterns of working hour characteristics and risk of sickness absence among shift-working hospital en	Rosenström et al 2021	Finland
2263	Bujacz 2021	Psychosocial working conditions of shiftworking nurses: A long-term latent transition analysis	Bujacz A et al 2021	Sweden
2227	Ropponen 2022	Association of working hour characteristics and on-call work with risk of short sickness absence amon	Ropponen et al 2022	Finland
2165	Johnsen 2022	The impact of clinical experience on working tasks and job-related stress: a survey among 1032 Norw	Johnson et al 2022	Norway
2136	Kjørstad 2022	Sleep and work functioning in nurses undertaking inpatient shifts in a blue-depleted light environmer	Kjørstad et al 2022	Norway
2034	Nielsen 2016	Meaning creation and employee engagement in home health caregivers	Nielsen et al 2016	Denmark
2011	Svedahl 2019	Increasing workload in Norwegian general practice – a qualitative study	Svedahl et al 2019	Norway
1986	Hult 2022	Health and work well-being associated with employment precariousness among permanent and temp	Hult, 202	Finland
1980	Heponiemi 2017	Finnish physicians' stress related to information systems keeps increasing: a longitudinal three-wave s	Heponiemi, 2017	Finland
1953	Mauno 2016	Emotional labour and work engagement among nurses: examining perceived compassion, leadership	Mauno et al 2016	Finland
1889	Lunde 2021	Associations of objectively measured total duration and maximum bout length of standing at work wi	Lunde et al 2021	Norway
1888	Westergren 2020	Associations between materials used and work-related musculoskeletal hand complaints among haen	Westergren et al 2022	Sweden
1873	Jepsen 2016	A qualitative study of how caseload midwifery is constituted and experienced by Danish midwives	Jepsen et al 2016	Denmark
1717	Blomberg 2016	Work stress among newly graduated nurses in relation to workplace and clinical group supervision	Blomberg K et al 2016	Sweden
1711	Hansen 2016	Night shift work and incidence of diabetes in the Danish Nurse Cohort	hansen et al 2016	Denmark
1673	Holmberg 2016	Job Satisfaction Among Swedish Mental Health Nursing Staff: A Cross-Sectional Survey	Holmberg, 2016	Sweden
1607	Lindegård 2016	Opting to wear prismatic spectacles was associated with reduced neck pain in dental personnel: A lon	Lindegård et al 2016	Sweden
1546	Henriksen 2016	Burnout among Norwegian midwives and the contribution of personal and work-related factors: A cro	Henriksen, 2016	Norway
1511	Lee 2021	Staff perspectives on the role of physical environment in long-term care facilities on dementia care in	Lee et al 2021	Sweden
1463	Ruotsalainen 2023	The mediating effect of psychosocial factors in the relationship between self-organizing teams and en	Ruotsalainen et al 2023	Finland
1450	Vedaa 2017	Short rest between shift intervals increases the risk of sick leave: A prospective registry study	Vedaa et al 2017	Norway
1418	Jacobsen 2022	Not too narrow, not too broad: Linking span of control, leadership behavior, and employee job satisfa	Jacobsen et al 2022	Denmark
1357	Pedersen 2020	Does accreditation affect the job satisfaction of general practitioners? A combined panel data survey	pedersen et al 2020	Denmark
1341	Kaltenbrunner 2019	Staff perception of Lean, care-giving, thriving and exhaustion: A longitudinal study in primary care	Kaltenbrunner et al 2019	Sweden
1300	Ose 2019	One-year trial of 12-hour shifts in a non-intensive care unit and an intensive care unit in a public hosp	Ose et al 2019	Norway
1292	Ropponen 2023	Working hour patterns and risk of occupational accidents. An optimal matching analysis in a hospital	Ropponen et al 2023	Finland
1288	Cohidon 2019	Practice organization characteristics related to job satisfaction among general practitioners in 11 cour	Christine Cohidon, Pascal Wild Nicolas Senn, 2019	Other: Different countries including Sweden and Norway
1277	Nurmeksela 2021	Relationships between nurse managers' work activities, nurses' job satisfaction, patient satisfaction, a	Nurmeksela et al 2021	
1223	Slåtten 2022	Precursors and outcomes of work engagement among nursing professionals—a cross-sectional study	Slåtten et al 2022	Norway
1202	Jørgensen 2021	Night work and incidence of Parkinson's disease in the Danish Nurse Cohort	Jörgensen, 2021	Denmark
1173	Bernstrøm 2020	Shift work and sickness absence at a Norwegian hospital: A longitudinal multilevel study	Bernstrøm VH et al 2020	Norway
1161	Stadin 2020	Healthcare managers' experiences of technostress and the actions they take to handle it - A critical in	Stadin et al 2020	Sweden
1133	Jakobsen 2018	Effect of physical exercise on musculoskeletal pain in multiple body regions among healthcare worker	Jakobsen et al 2018	Denmark
1052	Vedaa 2017	Sleep Detriments Associated With Quick Returns in Rotating Shift Work: A Diary Study	Vedaa et al 2017	Norway
1022	Jepsen 2017	Is caseload midwifery a healthy work-form? – A survey of burnout among midwives in Denmark	Jepsen et al 2017	Denmark
1001	Jørgensen 2017	Shift work and overall and cause-specific mortality in the Danish nurse cohort	Jörgensen, 2017	Denmark
978	Kader 2021	Shift and night work during pregnancy and preterm birth - a cohort study of Swedish health care emp	Kader, 2021	Sweden
962	Grønstad 2019	Organizational change and the risk of sickness absence: A longitudinal multilevel analysis of organizat	Grönstad, Kjekshus, Tjerbo & Bernström 2019	Norway
902	Nielsen 2019	Short time between shifts and risk of injury among danish hospital workers: A register-based cohort st	Nielsen et al 2019	Denmark
860	Heponiemi 2021	The association between using a mobile version of an electronic health record and the well-being of r	Heponiemi et al 2021	Finland
806	Jakobsen 2022	Factors associated with high physical exertion during healthcare work: Cross-sectional study among h	Jakobsen et al 2022	Denmark
741	Erdem 2017	Mechanisms of breast cancer risk in shift workers: Association of telomere shortening with the durati	Erdem JS et al 2017	Norway
724	Kader 2022	Night and shift work characteristics and incident ischemic heart disease and atrial fibrillation among h	Kader, 2022	Sweden
714	Heponiemi 2019	Usability factors associated with physicians' distress and information system-related stress: Cross-sec	Heponiemi, 2019	Finland
700	Bigert 2022	Night and shift work and incidence of cerebrovascular disease – a prospective cohort study of healthc	Bigert C et al 2022	Sweden
669	Golvani 2021	Operating room nurses' experiences of limited access to daylight in the workplace – a qualitative inte	Golvani, Roos & Henricson, 2021	Sweden
585	Vinstrup 2020	Physical exposure during patient transfer and risk of back injury & low-back pain: prospective cohort s	Vinstrup et al 2020	Denmark
560	Andersen 2019	Physical and psychosocial work environmental risk factors for back injury among healthcare workers:	Andersen LL et al 2019	Denmark
559	Riisgaard 2017	Associations between degrees of task delegation and job satisfaction of general practitioners and thei	Riisgaard et al 2017	Denmark
548	Vilén 2022	Prevalence of Hoarseness in Primary Health Care and Hospitals—Associations With Different Work Ta	Vilén et al 2022	Finland
503	Thun 2018	A study of unreasonable illegitimate tasks, administrative tasks, and sickness presenteeism amongst N	Thun et al 2018	Norway
490	Vilén 2021	Hoarseness among nurses	Vilén et al 2021	Finland
487	Ruotsalainen 2020	Which factors are related to Finnish home care workers' job satisfaction, stress, psychological distress	Ruotsalainen et al 2020	Finland
434	Vainiomäki 2020	Tailoring ehrs for specific working environments improves work well-being of physicians	Vainiomäki 2020	Finland
368	Gyllensten 2017	Experiences of reduced work hours for nurses and assistant nurses at a surgical department: A qualita	Gyllensten, Andersson & Muller 2017	Sweden
330	Poikkeus 2020	Relationships between organizational and individual support, nurses' ethical competence, ethical safe	poikkeus et al 2020	Finland
303	Kjellström 2017	Work motivation among healthcare professionals: A study of well-functioning primary healthcare cen	Kjellström et al 2017	Sweden
236	Gamskjaer 2022	Investigating job satisfaction in palliative rehabilitation: Reflections and perspectives of health profes	Gamskjaer et al 2022	Denmark
181	Thapa 2021	Support and resources to promote and sustain health among nurses and midwives in the workplace: /	Tharpa et al 2021	Sweden
178	Grønstad 2020	Work-related moderators of the relationship between organizational change and sickness absence: A	Grönstad, Kjekshus, Tjerbo & Bernström 2020	Norway
149	Golay 2022	Effortlessness and Security: Nurses' Positive Experiences With Work-Related Information Technology	Golay et al 2022	Sweden
132	Ose 2022	Perceived Causes of Work-Related Sick Leave Among Hospital Nurses in Norway: A Prepandemic Stud	Ose et al 2022	Norway
124	Sigursteinsdóttir 2020	Stressful factors in the working environment, lack of adequate sleep, and musculoskeletal pain among	Sigursteinsdóttir et al 2020	Island
110	Vedaa 2019	Short rest between shifts (quick returns) and night work is associated with work-related accidents	Vedaa et al 2019	Norway
65	Golay 2022	Negative Emotions Induced by Work-Related Information Technology Use in Hospital Nursing	Golay D et al 2022	Sweden
55	Fallman 2022	Managerial approaches for maintaining low levels of sick leave: A qualitative study	Fallman SL et al 2022	Sweden
34	Andersen 2021	Work-related violence and organizational commitment among health care workers: does supervisor's	Andersen LP et al 2021	Denmark
20	Westergren 2022	Work-related musculoskeletal complaints among haemodialysis nurses: An exploratory study of the w	Westergren and Lindberg 2022	Sweden
16	Spännargård 2022	Self-perceived clinical competence, gender and workplace setting predict burnout among psychother	Spännargård et al 2022	Sweden
15	Møller 2022	A cross-sectional national study of burnout and psychosocial work environment in vascular surgery in	Möller, 2021	Denmark
12	Grasmo 2021	Home care workers' experiences of work conditions related to their occupational health: a qualitative	Grasmo, Liaset & Redzovic, 2021	Norway
4	Liss 2018	Professional competencies and work-related support in relation to periodontal therapy and work sati	Liss et al 2018	Sweden

Aim of study	Study design	Start date (year)	End date (year)
To test the differences between the primary nursing model and the individual patient allocation model in: (1) work-related motivational characteristics; (2) work-related stress factors; and (3) job satisfaction, as reported by nurses working at one university hospital	Cross sectional study		
In this study, the primary objective was to investigate associations between the RNs perception of the safety cul-ture in ICUs, and burnout and sense of coherence. The secondary objective was to compare the scores for burnout and sense of coherence among the RNs in restructured and not restructured ICUs.	Cross sectional study	2012	2013
two aims in this study are to identify the meaningful types of rewards and the possible consequences of rewards as expressed by RNs	Qualitative research	2014	2014
to explore the influence of jobresources and job demands on bullying and three self-reportednurse outcomes. The selected outcome variables were job perfor-mance, job satisfaction, and work ability.	Cross sectional study	2014	2014
The aim of the present study was to examine the association between workplace relationships, witha focus on colleague belongingness, and self-rated health among employees in a Swedish municipal elderly health care organization.	Cross sectional study	2015	2015
The aim of this study was to examine whether changes in work shifts and shift intensity are related to changes in difficulties to fall asleep, fatigue, and sleep length.	Cohort study	2008	2015
The aim of this study was to examine the status of night work as a risk factor for common mental disorders (CMD)	Cohort study	1997	2012
to examine how intensive care nurses experience the effects of shift work on life outside work	Cross sectional study		
We aim to study sleep and psychosocial factors at work among permanent night workers by comparingthem to day workers and three shift workers.	Cross sectional study	2012	
The aim of the study was to explore registered nurses'workdayevents in relation to inner work life theory to better understand whatinfluences registered nurses to remain in work.	Qualitative research	2016	2016
The aim of this study was to investigate the association between working-hour characteristics in shiftwork and the incidence of short (1–3days) sickness absence among hospital employees	Case control study	2008	2015
The aim of the study was to investigate how restricted decision-making autonomy and conflicting demands impact operational managers' work performance and health	Cohort study	2012	2013
whether continuous exposure to shift work would be associatedwith the risk for increased fatigue and changes in sleep length over24 hr.	Cohort study	2008	2014
To investigate the association of different dimensions of night work, expressed by frequency and duration of night shifts throughout pregnancy, with the risk of severe PPD	Cohort study	2007	2015
we aim to assess how shift work characteristics affect the risk of occupational, transport and leisure-time injurie	Case control study	2008	2015
to clarify factors that support and prevent managers' work wellbeing by reviewing international research literature and interviewing Finnish social and healthcare managers	Qualitative research	2017	2018
we studied, first, age group differences in objective working-hour characteristics among women in hospital work and, second, the associations of working-hour characteristics with short (1–3days) sickness absence in different age groups	Cohort study	2008	2017
to examine the association between the type of shiftwork schedule and duration, and the incidence of dementia in the Danish nursing cohort, using detailed exposure information assessed at three different time points	Cohort study	1993	2009
to explore which factors are important in terms of experienced nurses' intention to stay in the clinical setting and to learn which factors affect their job satisfaction.	Qualitative research	2018	2019
the aim of the present study was to investigate how a reduction or an increase in the number of QR over time are associated with the risk of nurses reporting occupational accidents.	Cohort study	2016	2018
We investigated the association of working hours with occupational injuries in hospital shift work	Cohort study	2000	2015
to investigate the association between timing and length of work shifts, short time between shifts (quick returns), number of consecutive nightshifts, and weekly working hours and the risk of long-term sickness absence (≥30 consecutive days) among female nursing personnel in the public healthcare sector in Denmark anc	Cross sectional study	2007	2015
The aim of the study was to investigate the effects of the implementation of software for participatory working time scheduling on realized working hour characteristics and changes in several wellbeing out-comes. Theseoutcomesincludedself-reportedsleeplength,shift-specificinsomniaandexcessivesleepiness,work-lifeconflict,[illegible]perceivedhealth.	Other: Quasi-experimental study	2015	2017
This study aimed to examine associations between shift work and mood disorders as well as the sleep problems of workers with differing chronotypes	Cohort study	2000	2017
Our goal was to explore how changes in the work schedule would affect the prevalence of SWD over time.	Cohort study	2015	2017
In this study we examine in detail associations between different shift work schedules (day, evening, night, and rotating) and incidence of major psychiatric disorders, including mood disorders, neurotic disor-ders, and substance use.	Cohort study	1993	2018
The aim of the present study was to use an intensive longitudinal design to determine whether variation in QR, both within and between individuals, was associ-ated with self-rated stress in newly graduated nurses	Other: Longtudinal study	2015	2018
The main aims of the paper were to: (i) characterize working hour patterns in shift work by means of permutation distribution clustering as a data-mining tool, and (ii) study associations between these shift work patterns and sickness absence	Cohort study	2008	2019
This study aimed to identify profiles of working conditions to which nurses were exposed to over time and investigate how changes in working conditions relate to shiftworking and health	Cohort study	2002	2006
we aimed to investigate the association of the characteristics of working hours with the risk of short (1–3 days) sickness absence among hospital phy-sicians.	Cohort study	2005	2019
to compare the workload and range of tasks between inexperienced and experienced GPs. Additionally, the study addresses the extent to which clinical experience affects the way GPs perceive their daily work, including perceived levels of unhealthy stress	Cross sectional study	2018	2018
to use both work and sleep diaries and actigraphy recordings to investigate nurses' sleep patterns, work function-ing, levels of stress, and mood state over a 2-week period during which they undertook shifts in either a BDLE (blue-depleted light environment) or a STLE (standard hospital light environment).The secondary ai	Cross sectional study	not known	not known
To extend the current knowledge of employee engagement by emphasising how caregivers experience meaning in their work.	Qualitative research	unknown	unknown
to explore how GPs and their co-workers in Norway perceive and tackle their workload, and their experiences and reflections regarding explanations for and consequences of increased workload in general practice	Qualitative research	2017	2018
We aimed to explore employment precariousness, health and work well-being among permanent and temporary nurses.	Cross sectional study	2020	
The present study aimed to examine the 9-year longitu-dinal development of SRIS (stress related to information systems) levels among Finnish physicians.	Cohort study	2006	2015
to examine whether three resources, that is, compassion, transformational leadership and work ethic feasibility, buffer against the negative effects of emotional labour on work engagement	Cross sectional study	2014	2014
We aim to de-termine the association between objectively measuredstanding at work and lower-extremity pain intensity(LEPi) in construction- and healthcare workers over a 2-year period	Cohort study	2014	2017
The aims of this study were therefore (1) to examine the association between the type of dialysis machine and disposables usedwith the occurrence of hand complaints among haemodialysisnurses and (2) to compare occupational risks [revised strain index(RSI)] of developing work-related MSDs of the distal upperextremit	Cross sectional study	2017	2019
To advance knowledge about the working and living conditions of midwives in caseload midwifery and how this model of care is embedded in a standard maternity unit.	Qualitative research	oklart (men efter 2014 då etikansökan godkänts?)	
The aim of the study was to investigate occupational stress among newly graduated nurses in relation to their workplace and clinical group supervision.	Cross sectional study	2012	2012
In this prospective cohort study, we investigated the association between shift work and incidence of diabetes over13 years among Danish female nurses who were members of the Danish Nurse Cohort.	Cohort study	1993	2013
The objective of this study is to identify factors having positive impact on job satisfactionamong Swedish psychiatric nursing staff in an inpatient psychiatric clinic.	Cross sectional study	2012	
The main aim ofthis study was to investigate the effects on self-reportedneck pain, clinically diagnosed conditions in the neck,perceived exertion, and self-reported work ability amongdental personal opting to use prismatic glasses duringclinical dental work	Cohort study		
This study aimed to assess burnout levels among Norwegian midwives and identify personaland work-related factors associated with burnout	Cross sectional study	2014	
This study aimed to explore staff perspectives of the physical environment in supporting their care practices for residents living withdementia in Canadian and Swedish long-term care facilities	Qualitative research	2013	2018
To examine the association between self-organizing teamwork practicesand job satisfaction and turnover intentions. Furthermore, we examined whether psychosocial factors acted as potential mediators	Cross sectional study	2020	2020
We investigated whether exposure to quick returns and night shifts could predict later sick leave, and to what extent personality traits associated with shift work tolerance predicted sick leave and/or moderated any such prediction by shift schedule characteristics	Cohort study	2013	2014
argue that employees in units with medium-sized spans of control observe more leadership and have higher job satisfaction. Furthermore, that span of control can affect leadership behaviors differently.	Cross sectional study	2020	2020
hypothesise accreditation to have a negative effect on GP job satisfaction.	Other: randomized field experiment	2016	2018
to examine the extent to which changes over time in Lean maturity are associated with changes over time in care-giving, thriving and exhaustion, as perceived by staff, with a particular emphasis on the extent to which job demands and job resources, as perceived by staff, have a moderated mediation effect	Cohort study	2015	2016
In this study, we aimed to perform a thorough qualitative study, to understand nurses' experiences and perceptions of working 12-hour shifts compared with the usual 8-hour shifts.	Cohort study	2015	2017
In this study our aim was to explore and identify working hour patterns among hospital employees working irregular working hours and to investigate the associations between the identified patterns and the risk of occupational accidents	Cohort study	2008	2018
use internationell compaisons to explore the structural and organizational factors associated with GP:s dissatisfaction at work	Cross sectional study	2015	2015
To describe the relationships between nurse managers' work activities, nurses' job satisfaction, patient satisfaction, and medication errors at the hospital unit level.	Cross sectional study	2017	2017
Health services organizations must understand how best to lower nursing professionals' turnover intentions, and increase their job satisfaction and the quality of care provided to patients. This study aims to examine whether work engagement (WE) is a significant predictor of the achievement of these preferred organizatic	Cross sectional study	?	?
In this study we aim to examine whether shift work is associ-ated with incidence of PD, by examining the effect of different shift work schedules (day, evening, night, rotating) and whether there is a dose–response relationship between duration (cumu-lative years) of different shift work schedules and incidence of PD	Cohort study	1993	2018
The aim of the present paper is therefore (1) to describe what shift-work arrangements exist at a large Norwegian hospital, (2) to investigate how these shift schedules relate to employees' sick-ness absence and (3) to investigate how individual differences in age, gender and parental status moderate the relationship betwe	Cohort study	2012	2016
the aim of this study was to describe healthcare managers' experience of technostress and their actions for handling it.	Qualitative research		
To evaluate the effect of workplace versus home-based physical exercise on pressure pain threshold (PPT) and musculoskeletal pain intensity in multiple body regions.	Randomised controlled trial	2013	2013
The aim of the present study was therefore to further examinethe specific sleep-related consequences associated with QRs, ascompared with other common shift transitions.	Cohort study		
To investigate burnout among midwives – including a comparison of the level of burnout in caseloading midwives and midwives working in other models of care who do not provide continuity of care.	Cross sectional study		
In this study, we examine the association between shift work and all-cause mortality and mortality due to CVD, cancer, diabetes, neurodegenerative and psychiatric dis-eases in the Danish nurse cohort (DNC)	Cohort study	1993	2013
the study aimed to investigate whether shift work or night work and long working hours during the first (Weeks 1–12), second (Weeks 13–28) and third trimester (Weeks 29–42) of preg-nancy were associated with an increased risk of PTB	Cohort study	2008	2017
to examine the different relationships betweensix unit-level changes (upsizing, downsizing, merger, spin-off, outsourcing and insourcing) and sickness absence among hospital employees	Cohort study	2011	2016
we aimed to assess how duration of time between shifts – and, specifically, quick returns – affect risk of injury. Additionally, we evaluated the association between injury and days since a quick return as well as the number of quick returns in the past week.	Cohort study	2008	2015
This study aimed to examine the association between using a mobile version of electronic health records (EHR) and perceived time pressure, stress related to information systems, and self-rated stress.	Cross sectional study	2020	2020
to investigate which factors are associated with high physical exertion during healthcare work	Cross sectional study	2017	2017
In this study, we sought to investigate telomere lenght (TL) variation as a potential mechanism of the association between long duration of night shift with several consecutive nights and the increased risk of breast cancer.	Case control study	1990	2009
This study aimed to examine the effects of various aspects of night and shift work on the risk of incident ischemic heart disease (IHD) and atrial fibrillation (AF) using detailed and registry-based exposure data.	Cohort study	2008	2016
	Cross sectional study	2017	
The aim of this study was to evaluate the effects of various aspects of night and shift work, regarding incident stroke and other CeVD, by using detailed and registry-based exposure data.	Cohort study	2008	2017
To describe operating room nurses' experiences of limited access to daylight in the workplace.	Qualitative research	2020	2020
we sought to create an exposure-matrix to identify associations between biomechanical load during patient transfer and the odds of back injury and LBP among healthcare workers.	Cohort study	2017	2018
The aim of this study was therefore to investigate physical and psychosocial work environmentalrisk factors for back injury during patient transfer among healthcare workers in hospitals.	Cohort study	2017	2018
the objective ofthis study was to investigate associations between de-grees of task delegation and job satisfaction of GPs and their staff in Danish general practice using the management of patients with chronic obstructive pulmonarydisease (COPD) as our case.	Cross sectional study	2013	2014
The aim of this study was to determine the current preva-lence of hoarseness among the nurses in six different occu-pational subgroups (registered nurses, primary care nurses,pediatric nurses, laboratory nurses, dental nurses, and mid-wives) in order to determine whether different occupational subgroups have different	Cross sectional study		
To describe the relationship between unreasonable illegitimate tasks and sickness presenteeism in physicians after controlling for variance in age, gender, role conflict, control over work pace, exhaustion and administrative tasks.	Cross sectional study	2012	2012
The aim of this study was to determine the current prevalence of hoarseness among nurses and also to identify potential environmental risk factors in their working environment.	Cross sectional study	2016	2016
The aim of this article is to explore the challenges, stressors, team work and management factors that are associated with home care staff members'well-being, job satisfaction and experienced care quality, and further, how staff members experience their work.	Mixed-method study	2018	2018
we examined the associations of EHR-related variables with time pressure andstress and how these associations diered according to working environment	Cross sectional study	2017	2017
To investigate the experiences ofreduced work hours and no lunch breaks among a groupof nurses and assistant nurses, with a particular focus onrecovery and psychosocial working environment.	Qualitative research	2015	2015
To examine relationships between nurses perceived organizational and individual support, ethical competence, ethical safety, and work satisfaction.	Cross sectional study	2014	2014
to carry out a deductive analysis of factors that influence professional work motivation on individual, organizational, and cultural level at well-functioning primary healthcare units	Qualitative research	2015	2015
The aim of this study was to investigate reflections and perspectives from health professionals working within palliative rehabilitation for elements of importance in relation to job satisfaction.	Qualitative research	2019	2019
to explore and gain a deeper understanding of how nurses and midwives experience their everyday work, with a view toward promoting and sustaining their work-related health.	Qualitative research	2018	2018
to examine if and how the relationship between unit-level downsizing and sickness absence is moderated by three salient work factors: temporary contracts at the individual-level, and control and organizational commitment at the work-unit level.	Cohort study	2011	2016
To understand the appraisals and emotions at the core of nurses' positive experiences with information technology use at work.	Qualitative research	2017	2020
The aim of this study was to identifythe causes of work-related sick leave among Norwegian hospitalnurses	Cross sectional study	2015	2015
we analyzed musculoskeletal pain/discomfort in the neck and neck area, the shoulder and shoulder area, and the lower back, stressful factors in the work environment, and adequate sleep among Icelandic nursing unit managers, along with the correlation between these three factors.	Cross sectional study		
aim was to examine the association between quick returns (<11h) and night shifts, and self-reported work-related accidents, near accidents or dozing off at work.	Cross sectional study	2016	2016
This study aims to provide a view of the concrete ways in which work-related IT use can compromise hospital nurses' well-being at work	Qualitative research	2017	2020
The aim of this study was to identify first-line managers'approachesfor maintaining low levels of sick leave among their health careemployees	Qualitative research	2016	2016
To examine the effect of immediate supervisor's support on affective commitment within the first month, 3, 6 and 12 months after exposure to work-related violence or/and work-related threats	Cohort study	2012	2016
The aim of this study wasto carry out an exploratory analysis of the work sit-uation of haemodialysis nurses from an ergonomicperspective.	Qualitative research	2019	2019
The overall aim of this study was to map the level of work-related burnout and fatigue among psychotherapists working in clinical set-tings and to investigate the relation between burnout and (a) person-related factors such as age, training, level of education, years in profession and perceived competence; and (b) work-rel	Cross sectional study		
	Cross sectional study	2020	
to explore the views of home care workers on how working conditions affect their safety, health, and wellbeing.	Qualitative research	2020	2020
The specific aims of the current questionnaire study were to explore and analyse DHs' self-reported views on:1.professional competencies and behavioural interventions in periodontal therapy,2.work-related support in the treatment of periodontitis patients and daily practice, and overall work satisfaction	Cross sectional study	2012	2012

Population description	Total number of participants	Findings:	1 Risk eller friskfaktor
nurses in hospitals	317		risk
Registered nurses at six different hospitals	143		Friskfaktor
nurses	20		friskfaktor
nurses public hospitals	2946		risk
nursing assistants, nurses’ aides, registered nurses, physiotherapists and occupational therapists in sp	997		Riskfaktor
Hospital employeesPhysicians were excluded due to missing information on on-call work. The mean ag[illegible] the employees was 40.8 years, and 93% were women (table 1) with various job titles	7727		Risk
Finnish healthcare workers	46 010		Riskfaktor
intensive care nurses from two secondary general Danish intensive care units	114		risk
all hospital employees from the six hospital districts with current contract of employment and usingTi[illegible]® shift scheduling software was included.	9312		Risk
Registered nurses, decision unit/emergency department	10		Friskfaktor
Hospital employees	12156		Risk/Friskfaktor
Operational hospital managers	162		Riskfaktor
hospital workers with various job titles.The main occupational titles in 2008 were nurse (37%), depart	3679		Riskfaktor
workers in public hospitals nationwide (majority were nurses or physicians)	25009		risk
Helthcare workers	13337		Riskfaktor
social and healthcare managers	7		frisk
Finnish Public Sector study - hospital employees	12 761 (women)		
nurses	The association between shift work and incidence of dementia among three different subsets of the cohort: A) n=18892 with shiftwork information assessed at one point in time, B) n=6048 with repeated shift work assessments, C) n=8059 with information on duration of shiftwork throughout their entire career		risk
nurses from six hospitals	28		frisk
Nurses	1692		Riskfaktor
Hospital employeesPhysicians (N=331) were excluded due to missing information on on-call work. Of t[illegible]al sample, 91% (N=18 700) were women and 9% men,	18700		Risk
nursing personnel, in hospital and social health care	38699		risk
hospital employees from three hospital districts	677		Frisk
Sjukhusanställda	10637		Riskfaktor
Nurses	1076		Friskfaktor
We used the Danish Nurse Cohort, which consists of 28 731 Danish female nurses who were >44 year:	19 964		Risk
Nurses	350		Quick return riskfaktor
Hospital employees	6029		Riskfaktor
Swedish nurses	2936		Riskfaktor
Hospital-based healthcare workers	2845		Riskfaktor
general practitioners	1032		risk
nurses, hospital	25		risk
caregivers at home care organisations	16		frisk
general practitioners and their co-workers	33		risk
permanent and temporary practical nurses and registered nurses who are working in health and socia	7925 (svarsfrekvens 8%)		Risk
Physicians	1095 (som svarat på alla tre enkäter, 2006, 2010 och 2015)		Risk
nurses	3466		frisk
Healthcare workers in Oslo	62		Riskfaktor
haemodialysis nurses	282		Riskfaktor
midwives from two different hospitals.	interviews with 13; participant observations of 12 (others)		frisk
Nurses who had recently graduated from three Swedish universities	113		Friskfaktor
female nurses	19 873		risk
nursing staff (i.e., registered and assistant nurses) in the general psychiatric clinic	118 (av 130), 65% kvinnor		Risk
Dentists, dental hygenists and orthodontic assistants	The interven-tion group consisted of 371 individuals and the referencegroup comprised 193 individuals		Friskfaktor
midwives registered with either one of the two midwifery unions in Norway. The two unions together	598		Risk
Nurses and care aids in long-term care facilities on dementia care	24 varav 9 i Sverige		Friskfaktor
Licensed practical nurses (N = 377), registered nurses, therapists and managers (N = 183), andother er	591		Friskfaktor
Hospital-based nurses	1538		Riskfaktor
nurses in Danish hospitals	1699		frisk
general practitioners	846		risk
staff at 46 primary care units	260		frisk
Nurses at one gastrointestinal surgery ward (non-intensive care unit (ICU)) and a highly specialised ne	24		Riskfaktor/Friskfaktor
Hospital-based healthcare workers	4419		Riskfaktor
General practitioners, GP:s	12049		Både risk och friskfaktorer är studerade
nurses in acute care hospitals	306		frisk
nurses public hospitals	164		frisk
We used data on female participants of the Danish Nurse Cohort, >44 years of age who reported infor	Analys A = 20138Analys B = 7006Analys C = 8063		
Hospital employees	14132		Riskfaktor
Helth care managers in public hospitals	20		Riskfaktor
Eighteen departments from three hospitals in Copenhagen	176		frisk
Nurses, Telemark county, Norway	67		Riskfaktor
midwives working in a tertiary maternity unit	50		frisk
female members of the Danish Nurses Organiza-tion aged >44 years	18015		Risk
health care professionals employed by Region Stockholm,from selected professions often working			Risk
hospital employees	26 252		frisk/risk
Healthcare workers	69200		Riskfaktor
Nurses	3610		Riskfaktor
health care workers	2047		risk
Norwegian nurses	563 breast cancer patients and 619 controls		Riskfaktor
healthcare professionals, We restricted the study to employees in Region Stockholm who often work [illegible]hysicians were not included in the present analysis due to less detailed information on working hours and night work.			
physicians younger than 65 years	4018 (response rate 21,9%)		Risk
nurses and nursing assistants	30460		Riskfaktor
Operating room nurses, surgical ward at a county hospital	15		friskfaktor
Healthcare workers	1285		Friskfaktor
Sjukhusanställda (nurses, nurses aids, healthcare assistants,occupational therapists, physical therapist	2080		Riskfaktor
General practitioners and their staff in Danish general practice management patients with chronic ob	1580		Friskfaktor
Registered nurses, primary care nurses, pediatric nurses, laboratory nurses, dental nurses, and midwi	15553		Riskfaktor
Norwegian physicians	545		Riskfaktor
Nurses	13560		Riskfaktor
Employees in home care organisations	179		Riskfaktor
physicians in public sector health care centers or hospitals	4018		risk
assistant nurses and nurses at orthopaedic surgery department at a large hospital in Sweden.	11		frisk
nurses in specialized, primary or private health care	298		frisk
professionals working at Swedish primary care units	43		frisk
Nurses, Psychologists, Physicians, Occupational Therapists, Physiotherapists, and Socialworkers, work	12		Friskfaktor
nurses and midwifes in hospitals and community healthcare facilities	13		frisk
full-time employees large hospital	21085		risk/frisk
registered nurses surgery ward, pediatric oncology ward, neonatal ward at large hospital	15		friskfaktor
Hospital nurses	1297		Riskfaktor
Icelandic nursing unit managers	110		Riskfaktor
Nurses	1784		Riskfaktor
RNs from different surgery and children's hospital departments	15		Riskfaktor
First-line hospital managers	11		Friskfaktor
Employees at psychiatric wards and psychiatric outpatient wards	398		Friskfaktor
haemodialysis nurses	19		Riskfaktor
clinically active psychotherapist working in psychiatric care or other mental health settings in Sweder	327		Friskfaktor
All active VSs (attend-ings and junior attendings) and VSTs (chief residentsand residents) employ	104		Risk
home care workers from three home care service units in a medium-sized city in Norway.	8		risk
Dental hygienists’ in the county of Västra Götaland	302		Friskfaktor

1 Modifierbar organisatorisk struktur	1 Hälsoutfall
nursing allocation models (primary nurse model vs individual patient allocation model)	stress
Hospital level safety culture	Burnout
Financial compensation and benefits	job satisfaction and commitment
institutional stress	job satisfaction work ability
Nattarbete	Hälsa
Long spells of work-shifts	
Nattarbete (skift med regelbunden natt)	Common mental disorders (ICD-10 F00-F99)
shift work (night and evening shift), schedule	mental symptoms
Skiftarbete	Svårt somna
Registered nurses’colleagues are scheduled together and can help and support each other intheir dail	Motivation
working-hour characteristics in shiftwork	Short-term sickness absence
Restricted decision-making autonomy	Self-rated health
Skiftarbete utan nattskift	Fatigue during work
night work during pregnancy	severe post-partum depression (PPD)
Quick returns	Injury (occupational, transport and leisure)
collaboration within the organization and with political decisionmakers	wellbeing
extended (>40hr) weekly working hours	short sick-ness absenc
type of shift work schedule and duration/persistence of night work	dementia
to be acknowledged, respected and informed by all levels of managementCo-determination and flexibility in planning work schedules	job satisfaction
Quick returns (increased)	Occupational accidents
No of quick returns (<11 h btw shifts)	Occupational injuries (excluding violence from patients)
working hour characteristics	long-term sickness absence
participatory working time scheduling software	Perceived health
Fixed night work	mood disorders and sleep problems
Schemaförändring QR	Shift work disease
Skiftarbete	Mood disorders
Arbetstider, skiftgång	Stress
working hour characteristics	Sickness absence
Skiftarbete	Burnout, Sleep disturbance
Working hour characteristics	Short-term sickness absence
activities:1) number of consultations2) nr of medical issues during consultations3) disagreement or difficult discussions related to pre-scribing potentially addictive medications	Self-perceived health
Blue-depleted light environments (BDLE)	medical conditions, mental health conditions
sufficient time and staffing	engagement
Transfer of tasks from secondary to primary care, administrative task, changes in legislation, higher d	health and motivation
Rights	SHIS
Jour eller inte jour	SRIS
ethic feasibility (value congruence; resources enabling work according to ethical standards)	engagement
Standing at work	Lower-extremity pain intensity (LEPi)
Sjukvårdsmaterial och -utrustning	WORK-RELATEDMUSCULOSKELETAL HAND COMPLAINTS
caseload midwifery	job satsifaction
Clinical group supervision	Stress
night shift work, evening shift work	diabetes
Lön	Job-satisfatcito
Prismatic glasses	Neck pain
2-skift	Work burnout
Physical environment	Job Satisfaction
Self-organizing teams	Job satisfaction
Schemaläggning - quick returns	Sickness absence
managers' span of control (SOC)	job satisfaction
accreditation	job satisfaction
lean (maturity)	exhaustion
12h arbets shifts på helg istället för 8h	Hälsa
Working hour patterns	Occupational accidents
mer än 50 timmars veckoarbetstid, begränsade möjligheter att erbjuda tid samma dag, Tillgång tillElel	Job dissatisfaction
	job satisfaction
organizational culture	work engagement (WE), job satisfaction (JS)
Typ av skiftarbete	Sjukfrånvaro, kort (1-8 dagar) och lång (>9 dagar)
Poor user experience	Technostress
möjlighet till fysisk träning på arbetet	pressure pain threshold (PPT) and musculoskeletal pain intensity in multiple body regions
Schemaläggning, QR	Sömn, självrapporterad stress
caseload midwifery	burnout (personal-, work- and client-related)
Skifttyp	All-cause mortality
Skifttyp	Preterm birth
unit level upsizing (= the unit increases personnel)	short-term (<=8 days) and long-term (>= 9 days) sickness absence
Time between shifts, Quick returns	Occupational injury
Using a mobile version of electronic health records	Self-reported stress
use of specific assistive devices and distribution of difficult patients through planning	physical exertion: “How would you rate your physical exertion while working with the patients?”
Nattarbete	Telomere shortening
Number of systems in daily use	Psychological distress
Nattarbete	Cerebrovascular disease (CeVD) and stroke
access to daylight in the workplace	wellbeing, joy, energy, ability. stress.
Use of assistive devices	Low-back pain and backl injury
Tillgång till lyfthjälpmedel	Ryggskada
Task delegation	Job satisfaction
Indoor climate	Hoarsness
Task allocation	Sickness presenteeism
Indoor air quality	Hoarsness
Working alone	Psychological distress
use of EHR	stress
working hours	energy for work, energy for life outside work, "living life, not just surviving" (experience of living a full
organizational support for nurses' ethical competence	work satisfaction
Org strukturer och processer för ledarskap, kommunikation och stöd; Org system för information om	motivation
Self-management	Job satisfaction
present and responsive manager, updated in latest treatment methods	job satisfaction
unit level downsizing (i.e. reduction in employees > 20%).	short-term sickness absence (<= 8 days). Register data.
hospital-wide electronic health record	positive emotions: joy, relief, confidence and relaxation
Möjlighet till anpassade arbetsuppgifter	Sjukfrånvaro
Kontrollspann	Adequate Night’s Sleep
Quick returns	Occupational accidents
IT-system	Negative emotions (alienation, anxiety, frustration, moral distress, perplexity, psychological distress)
Ledarskap	Sjukfrånvaro
Stödåtgärder i samband med hot och våld-händelser	Engagemang
Sjukvårdsutrustning	Work-related musculoskeletal complaints
Deltagande i klinisk handledning	Utbrändhet
Weekly working hours	Work-related Burnout
shift work	poorly rested (tired)
Kompetensutveckling på arbetstid	work satisfaction

1 Resultat	1 Modifierbar faktor i arbetsmiljön (krav/resurs)
The nurses working according to PNM regarded their work as more stressful as a whole, but between the two groups there was no statistically significant difference (P=0.136). Nurses who exercised the primary care nursing model had more work-related stress, from pressure from work not carried out and from the quantitative inadequacy of the nursing staff, than nurses working in IPAM (P=0.002).	
The hospital level safety culture was associated with decreased levels of burnout (r=-0,477, p<0.001)	
monetary rewards, discounts, exercise facilities, etc: a reward that reinforced occupational satisfaction and encouraged respondents to perform their work correctly.	
institutional stress (b=-0.30, p<.001) reduce job satisfaction. institutional stress was not significantly related to workability.	reduces job satisfaction directly and mediated by bullying
Inget samband mellan att arbeta natt och hälsa kunde ses	
- Fatigue during work 1,10 (0,84-1,42)- Fatigue during free days 1,25 (0,98-1,64)- Svårt att somna 1,03 (0,78-1,38)- Short 24-h sleep 1,13 (0,90-1,45)- Long 24-h sleep 0,80 (0,63-1,03)	
changing to night work was not associated with the odds of acquiring CMD [odds ratio (OR) 1.03, 95% confi-dence interval (CI) 0.82–1.30]	
A significantly higher percentage of night-shift workers had symptoms when working shifts compared with evening-shift workers, with mood swings (p .03) and headaches (p. 001) being the most common	
The PNWs reported most often difficulties to fall asleep(15.7% vs. 13.6% in the SW and 11.5% in the DW, p< 0.015, adjusted p< 0.001)	
	Sufficient resources and the opportunity to work together on right tasks
Exposure to >2,and especially >4 consecutive night shifts associated with an increased likelihood of short sickness absence (OR1. 24,95% CI1.12–1.38 and OR1.54, 95%CI 1.10–2.15, respectively) among shiftworkers, whereas a high number (>25%) of evening shifts and having >2 consecutive evening shifts associated with lo	
Restricted decision-making autonomy was predictive of T2 SRH (β=−0.140, p=0.037)	
1,04 (0,93-1,17)	
no increased risk of PPD for any of the dimensions of night work. increased risk of PPD (OR 2.08, 95% CI 1.09–4.00) among women who stopped working night shifts after the first pregnancy trimester.	
In the adjusted analyses, the odds of an occupational injury were higher during evening compared with day shifts (OR 1.54, 95%CI 1.43–1.66) and quick returns compared with regular returns (OR 1.26, 95%CI1.10–1.44).No higher odds of a leisure-time injury or leisure time transport injury were observed after evening shift	
Working-hour characteristics associated with the length of working hours showed minor associations with short sick-ness absence across all age groups of the shift working women. Also, extended (>40hr) weekly working hours and the number of short (<11hr) quick returns were linked, although in small magnitude, with sh	
Nurses working nightshifts ≥6 years had higher dementia incidence (hazardratio: 2.43, 95% CI 1.39-4.23) than those working dayshifts ≥ 6 years. Nurses working nightshifts ≥ 6 years had higher dementia incidence than those working nightshifts <1 year (1.47, 1.06-2.06).	night work
found seven themes in relation to job satisfaction: being an experienced nurse, the importance of the speciality, management, professional challenges, good colleagues, balancing family and work–life and change in organisations.	
An increase from having no or a moderate number of QR (1–34 per year) from baseline to the two-year follow-up assessment was not associated with an increased risk of occupational accidents, compared to experiencing no change in the number of QR	
OR 1.40, 95% CI 0.68–2.89 for two quick returns during the preceding week, table 4)	
The Danish data showed that higher risk of longterm sickness absence was associated with having evening work (>50 shifts/person/year: IRR 1.18, 95% CI 1.06-1.34) or five or more consecutive nightshifts (more than 12 times/person/year: 1.88, 1.43-2.48). nightwork was associated to higher risk of sickness absence when excluding pregnant women.	
1,17 (0,15-9,32)	
fixed night work was associated with mood disorders among somewhat evening (adjusted odds ratio [OR] 1.91, 95% CI 1.09–3.34) and definite evening-type workers (adjusted OR 2.05, 95% CI 1.06–3.98) compared to day workers. Shift work with night shifts was associated with mood disorders among definite evening-type	
Reduction in numbers of QRs worked last year (>10 decrerase) was associated with an increased recovery from SWD (OR 1.82 (1.14 - 2.90)	
- Day = referens- Kvällsskift 1,51 (1,19-1,92)- Nattskift 1,69 (1,24-2,29)- Roterande skift 1,09 (0,89-1,34)	
Många QR, under en vecka jämfört med en genomsnittlig vecka ökade den upplevda stressnivån	Arbetstidsförläggning
The strongest risk of SA was associated with highly irregular working hours with night and weekend shifts and interrupted job contracts.	
he current analyses suggest that shiftworking nurses are particularly in need of measures that address poor work environments. Notonly do they experience more negative psychosocial work environments than their dayworking counterparts, but they do so while having to contend with demanding work schedules.	
After controlling for weekly working hours and the number of normal (≤12 h) shifts, a higher number of long (>12 h) shifts (ORs for ≥5 versus none: 2.54, 95% CI 1.68–3.84), very long (>24 h) shifts (ORs for ≥5 versus none: 2.62, 95%CI 1.61–4.27), and on-call shifts (OR for ≥5 versus none: 2.15, 95% CI 1.44–3.21) and a higher	
no direct association between the number of consultations performed and negative health impact. However, physicians who fully/partly agreed that their job affected their health negatively tended to han-dle a slightly higher number of medical issues and experienced more conflictual situations throughout the day compa	stress, demands
increase in sleepiness (by 17%) during evening shifts in the BDLE compared with the STLE (p=.034; Cohen’s d=0.49).There were no significant differences on any sleep measures (either based on sleep diary data or actigraphy record-ings) nor on self-reported levels of stress or mood across the two conditions.	
thematic analysis reveal three distinct patterns in how caregivers experience engagement: 1) caring for, nurturing the patients, 2) when the work fulfilled their notion of professionalism, 3) the security of the job, and working with people.	experience of meaning in work when spending more time with the patients, demonstrating empathy
impression that workload has been increasing. Explanations for higher workload:1) Transfer of tasks. Increasing transfer of medical tasks from secondary to primary care and increasing demands for administrative tasks and diagnostic investigations2) Increased work per patient. changes in legislation, higher demand for documentation	workload, need for communication/cooperation with other parts of healthcare system
p<0,001	
he effect of on-callburden did not vary across the different waves, but ithad a significant between-subjects effect (F = 4.86,p= 0.008), indicating that those who had an on-call bur-den in every wave had higher levels of SRIS in everywave as well (Fig. 2).	
Higher emotional labour related to lower engagement. Two interaction effects were found. Work ethic feasibility buffered against emotional labour (vilket gjorde work engagement möjligt trots krävande emotionellt arbete):Interaction term between Emotional Labour and work ethic feasibility: (beta=0.09, p < 0.001,ΔR2=0.0	emotional labour
Analyses on healthcare workers showed no associations between static and dynamic standing and average LEPi during follow-up	
There were no differences in the prevalence of hand complaints based on the type of haemodialysis machines,dialysers or tubing used. There were no differences found in physical exposure to the hands during priming, based onmachine type used	
The emerged essential statement was that caseload midwifery is a work form with an embedded and inevitable commitment and obligation that brings forward the midwife's desire to do her utmost and in return receive appreciation, social recognition and a meaningful job with great job satisfaction.	being able to use skills: ‘I feel that I am able to use all the skills that I havebeen trained with’(Maya, M
Ratings of stress were significantly lower among those who received clinical group supervision than among those who did not (p0.002). The nurses who received supervision reported moderate stress (md 6,q14, q37, range 2–9), while nurses without clinical group supervision reported higher than moderate stress (md 8,	
Compared with nurses who worked day shifts: increased risk of diabetes in nurses who worked night (1.58; 1.25 - 1.99) or evening shifts (1.29; 1.04 - 1.59) in the fully adjusted models	
p=0,019	
A significant decrease in neck pain (p= 0.047), clinical diagnoses in the neck (p= 0.025), and perceived exertion(p= 0.003) was observed at follow up for the intervention group compared to the reference group.	
2,1 (0,9-5,0)	
Thefindings demonstrate that well-designed environment qualities such as homelike ambience, an open layout, and stimulating courtyard positively stimulate the emotion of staff as well as residents, which also leads to build trust and relationship and to increase job satisfaction.	
Those employees who worked in a self-organizing team were more satisfied with their job and had lowerturnover intentions compared to those in a non-self-organizing team (mean [SD] 3.9 [1.0] vs. 3.7 [1.0], p = 0.006 and2.2 [1.2] vs. 2.5 [1.3], p = 0.006, respectively)	working in self-organizing teams may enhance employeewellbeing by lowering job demands and job s
Exposure to QR the previous month increased the risk for sick leave days (incidence rate ratio (IRR)=1.066, 95% CI 1.022 to 1.108, p<0.01) and sickleave spells (IRR=1.059, 95% CI 1.025 to 1.097,p<0.001) the following month,	
higher job satisfaction under medium SOC. SOC (reference group=medium) and job satisfaction, standardized path coefficients SEM: narrow SOC -.01 (p < .01); broad SOC -.09 (p < . 05)	active leadership behavior
not significant. No evidence of accreditation affecting GP job satisfaction. p < 0.05However, there are negative associations between job satisfaction and perceiving accreditation as a tool for external control: −0.39 (-0.69;-0.09) p < 0.05and positive associations between job satisfaction and agreeing that accreditation is a tool for quality improvement: 0.42(0.13;0.71)p < 0.05	
Increased Lean maturity was associated with decreased staff exhaustion, mediated by decreased job demands. The indirect effect was estimated to −0.07 (95% CI−0.12 - −0.03). The direct effect (controlling for the mediator) was non-significant	job demands: quantitative demands, Work pace, Emotional demands, Cognitive demands, Role conflicts
The experiences of working 12-hour shifts differed considerably between participants, especially those in the ICU. Their individual experiences differed in terms of health consequences, effects on their family, appreciation of extra weekends off, perceived effects on patients and perceived work task flexibility.	
Compared to the cluster with the fewest accidents, clusters with a higher proportion of accidents were characterized by late work shifts and a high proportion of quick returns (<11-hour shift interval,) and long work shifts (>12-hour shift), RR 1.31, 95 % CI 1.13–1.52 for the cluster with the most accidents.	
Högt missnöje med arbetet relaterat till lång veckoarbetstid, svårigheter att få in patienter på sjukhus, begränsade möjligheter att ge patienter tid samma dagLågt missnöje med arbetet kopplat till elektroniska journalsystem, tillgång till vårdsamordnare	arbetstidmöjlighet att ha elektronisk journaltillgång till resurs i form av vårdsamordnare eller dyl
IMOC (standardized coefficient 0.278) and CC had a positive effect on WE (p<0.05). WE had a positive effect (standardized coefficient 0.833, p<0.01) on JS. The model explains 32% of the variance in WE and 69% of the variance in JS.	collaboration climate (CC)
The results showed increased risk of short-term sickness absence for two-shift and three-shift rotations, as well as fixed night shifts compared with fixed-day shifts. We also found an increased number of absence periods for two-shift rotations without nights and three-shift rotations. results for long-term sickness absence v	
Time consuming ICT systems and malfunctioning/disturbing ICT systems were associated with technostress	
In some of the body regions, PPT and pain intensity improved more following physical exercise program carried out at work than at home. Between-group differences at follow-up (WORK vs. HOME) were 41 kPA [95% CI 13–70, effect size (ES): 0.22] for PPT in the lower back, and−0.7 [95% CI -1.0–0.3, ES: 0.26] and−0.6 points [95% CI -0.9–-0.2, ES: 0.23] for pain intensity in the lower back and feet, respectively.	
None of the other shift transitions studied encumbered as many detriments as QRs, which included short sleep duration (5.6 hours), slightly prolonged sleep onset latency, more abrupt ending of main sleep period, increased sleepiness, and higher level of perceived stress on the following shift	
In caseload midwifery the burnout scores in personal- (p 0.04), work- (p. 0.004) and client-related (<0.001) burnout were lower than ‘’other models of care”.	
- Dag = referens- Kväll 1,29 (1,11-1,49)- Natt 1,26 (1,05-1,51)- Roterande 1,00 (0,88-1,15)	
- Dag = Referens- dag/kväll 0,96 (0,68-1,36)- dag/kväll/natt 1,19 (0,76-1,85)- ständig natt 1,42 (0,76-2,63)	
reduced risk of both short- and long-term sickness absence in the change quarter (OR = 0.77; 95%CI = 0.73–0.80 and OR = 0.89; 95% CI = 0.83–0.95, respectively) and in the subsequent quarter (OR = 0.85;95% CI = 0.81–0.89 and OR = 0.88; 95% CI =0.82–0.94, respectively).	
Results showed the shorter the time between shifts, the higher the risk of injury. Thus, an elevated risk of injury was observed after quick returns compared with the standard 15–17 hours between shifts (IRR 1.39, 95% CI 1.23–1.58). Furthermore, when assessing the number of days since a quick return, the risk of injury wa	
The use of mobile version of EHR was not associated with self-rated stress in a multivariate logistic model with Age, gender, employment sector	
Factors associated with high physical exertion (OR and 95% CI) were high frequency of daily patient transfers1.35 (1.23 – 1.48), less self-reliant patients 0.74 (0.62 – 0.89), less frequent use of necessary assistive devices 1.82 (1.50 –2.21), as well as more frequent use of sliding pieces 1.23 (1.04 – 1.46), wheelchairs 1.23 (1.02	fysiska krav
TL is affected by intensive night work schedules, as work with six consecutive night for a period of more than 5years was associated with decreased telomere lengths (−3.18, 95% CI: −6.46 to −0.58, P=0.016)	
p=0.06 model Ap=0,46 model B	
observed an excess risk of CeVD (N=223) among employees who, during the preceding year, worked night shifts >30 times [hazard ratio (HR) 1.44, 95% confidence interval (CI) 1.04–1.99] or ≥3 consecutive night shifts >15 times (HR 1.69, 95% CI 1.18–2.42) compared to those who did not work nights. also observed an exces	
daylight was considered important for experiencing well-being, to a sensation of joy, increased awareness and energy which seemed to improve the ability to perform at work.	having daylight in the physical environment
Exposure profiles are provided for 9 groups of assistive devices, with ceiling lifts and intelligent beds elicitingthe lowest physical exposure. In the fully-adjusted model, we report differences in LBP intensity at follow-up betweenthe low and moderate exposure groups (p= 0.0085). No difference was found between the mode	
I 26,4% av de ryggskador som skedde i samband med patientförflyttning uppgavs det sakna nödvändiga lyfthjälpmedel	
The staff’s overall job satisfaction showed a significant association with “maximal degree” of task delegation compared to “medium degree” (OR = 1.88,p-value =0.048).	
There was a positive association between reporting environmental problems (draft, high, low or variable room temperature, dry air, stuffy indoor air etc) and the risk of hoarseness among all the participants when adjusted for age, gender, smoking, and occupational subgroups	
indicate that the unreasonable illegitimate tasks variable was a positive predictor of sickness presenteeism. The OR for unreasonable illegitimate tasks was 1.69 (p< 0.01)	
Our results suggest that the one-year period prevalence of hoarseness was 30% and all the environmental problems which we evaluated(draft, room temperature too high, variable room temperature, room temperature too low, dry air, stuffy indoorair, moist air/ high humidity, inadequate ventilation, smell of mold or cella	
There was an significant association between working alone and psychological distress, both in univariate and multivariate models corrected for age and sex	
Experiencing technical problems with EHR: more likely to experience stress (OR 1.29 p 0.002) compared to those experiencing low levels of technical problems. The interaction between working environment and technical problems was significant for self-rated stress (Wald’s=11.84,p =0.001). Technical problems were relate	technical problems with EHR
reducing working hours to to 6 hours/day with no reduction in salary and eliminating the lunch break was reported to result in more energy for work and energy for life outside work - for living life, not just surviving.	reduction in physical and cognitive demands, better social climate, more stable social climate (people
Organizational support (B= 0.449, CI [0.415, 7.803], p<.001) is a significant predictor of work satisfaction.	ethical competence for addressing ethical problems in the workplace
identifierar faktorer på individ-, organisation- och kulturell nivå. Organisatoriska faktorer: The centers’ positive management was due to a unique combination of factors, such as clear direction of goals, a culture of non-hierarchical collaboration, and systematic quality improvement work. The financial incentives need to be tr[illegible] to provide clear direction for the professionals. Social processes where professionals work together as cohesive groups, and provided space for quality improvement work, is	[illegible]
Self-management was one of the subthemes identified regarding the workers job satisfaction	
When nurses and midwives perceived their work environment to be supportive – that is, an environment in which opportunities are available to strengthen work qualifications, social and collegial teamwork are encouraged, and strategies for recovery are effectively developed and implemented – they viewed this en	competence opportunities,present and responsive manager,
decreased risk of short-term sickness absence in the quarter before (OR=0.68; CI 0.63-0.73) and an increased risk of short-term sickness absence in the quarter after (OR 1.08; CI 1.00-1.17) unit-level downsizing.	
Appraisals of easy goal accomplishment was connected to feelings of joy. Doing less of a particular task (e.g. documentation) was connected to feelings of relief. Knowing what the situation is and at has to be done, and mastering the system was connected to feelings of confidence. Reduced risk of mistakes and omissions, a[illegible]	[illegible], communicating via IT
I fritextsvar angav 18 respektive 14 av 206 fritextsvar att de uppskattade att deras arbetsrelaterade sjukfrånvaro orsakats av bristande anpassning på grund av graviditet och hälsoproblem	
he results revealed no statistical differences among an adequate night’s sleep and age, seniority in a management position, number of full-time positions in nursing, and workday length	
The number of QRs during a year was positively associated with seven of the eight items on work-related accidents, near-accidents and dozing off at work, and number of night shifts was positively associated with five items. Some of the key findings were that QRs were associated with nurses causing harm to themselves (i	
Our study shows that work-related IT use contributes to nurses' experience of several negative emotions at work and that these negative emotions emerge from nurses' perception of IT hindering them in their work	Mental effortBeing unable to carry out a taskDoing extra or unnecessary workFailing to complete a tas
Holistic approach to meet the needs of employees	Flexibility regarding employees’ working conditionsFlexibility regarding employees’ personal situations
Employees receiving very high levels of social support from supervisors immediately after being exposed to work-related violence or threats had a significantly higher level of organizational commitment across all four time points when compared to the group experiencing middle/low levels of support. Furthermore, at 1- ar	
The findings support the hypothesis that there might be an association betweenmaterials used and the development of work-related complaints involving the fingers, hands and wrists of this population.	
Inget samband kunde ses mellan att delta i klinisk handledning (vilket 74% av de svarande gjorde) och utbrändhet	
P=0.62	
disturbed sleep patterns and reduced sleep quality that shift work causes lead to feelings of unrefreshed and poorly rested	
Opportunity to attend courses within working hours to develop one’s competence were associated (boarderline) with work satisfaction (OR 1.8 (95% CI 0.96-3.43, p=0.067) when investigated in a multiple logistic regression model along with background- and workplace factors	

1 Kommentar	2 Risk eller friskfaktor	2 Modifierbar organisatorisk struktur	2 Hälsoutfall	2 Resultat
Primary Nursing Model: one nurse is responsible and accountable for the total care of a small group of patients from the time of admission to the time of discharge.	risk	primary nursing model	job satisfaction	In PNM wards, nurses were more satisfied with possibilities for personal growth and development offered by the work: but not significant difference (P=0.155).
Hospital level safety culture: Hospital management support for patient safety, teamwork across hospi	Riskfaktor	Restructured ICU unit	Burnout	Working in a restructured ICU unit was not associated with increased levels of burnout compared to
	friskfaktor	schedule, employment stability	job satisfaction and commitment	structure of work schedules, accommodation of desires for shifts, the ability to 'save' time in a 'worki
institutional stress related to the organization's policy, lack of power and influence, values that conflic				
SHIS measures twelveaspects of health, including: having energy, feelingcalm/relaxed, and feeling o				
Odds ratio (OR) refer to every 25% increase in the occurrence of the 3-month prevalence (%) of the c	Risk	>2 consecutive night shifts		- Fatigue during work 1,10 (1,05-1,19)- Fatigue during free days 1,10 (1,03-1,16)- Svårt att somna 1,13 (1,05-1,22)
	Friskfaktor	Nattarbete (skift med regelbunden natt)	Common mental disorders (ICD-10 F00-F99)	night workers with CMD had higher odds of recovery from CMD when changing to non-night work co
PNW = permanent night workersDW = day workSW = shift work	Risk	Skiftarbete	fatigue during free-time	fatigue during free-time (23.2% vs. 19.9% in the SW and 14.1% inthe DW,p< 0.001 adjustedp< 0.001)
	Friskfaktor	Registered nurses get the right support from other professions; right competence in the rightplace	Motivation	
Sample items are "Decisions taken further up in the or-ganisation are very difficult or impossible to in				
Relativa risker. Jag har valt resultat från den mest justerade modellen och 6-årsuppföljningenFinns även data som visar samband mellan förändringar i skiftarbete och utfallen (tabell 4) men jag tog inte med dom här			Fatigue during free days	1,27 (1,04-1,55)
dimensions of night work: number of night shifts, duration of night shifts, consecutive night shifts, quick returns.				
	risk	non-transparent policies in organization; Short-sighted planning; Economic fluctuation	hinders for wellbeing	
Lite knepigt då fokus för artikeln är att jämföra risker i olika åldrar - alla resultat presenteras i därför i		quick returns (< 11h)	short sick-ness absenc	
"management" var inte bara ledarskap på arbetsplatsnivå utan även organisatoriskt: The nurses reque	risk	unwilling change of unit	job satisfaction	occasional unwilling change of unit was stressful and had a negative impact on their job satisfaction
	Friskfaktor	Quick returns (decreased)	Occupational accidents	Those who had many QR (>34 per year) at baseline but experienced a reduction after two years had a
Högsta risken var för 2 returns, finns data för ytterligare 4 variabler men tveksamt om vi ska ta med d	Risk	Total weekly working hours	Occupational injuries (excluding violence from patients)	he length of the total weekly working hours of the preceding week was not associated with the risk fo
oddskvotFörutom detta finns oxå resultat för "svårigheter att somna i samband med ... ... och sen kommer olika arbetspass, dag, kväll, natt, lediga dagar.				
Chronotype was assessed using one item from the Diurnal Type Scale (Torsvall and Akerstedt 1980) in				
	Friskfaktor	Schemaförändring night shifts	Shift work disease	Terminating night work was the strongest predictor for recovering from SWD from baseline to follow-
Hazard ratiosAnger resultat från analyser som bygger på utfall baserat på sjukvårdskontakt (inte förskrivna läkemedel)				
Lite osäker på denna - här har man kopplat olika profiler/kluster till schema och hälsoutfall men inga				
	risk	cost-efficiency	engagement	potential risks to engagement: increasing time pressures; constraints, cost-savings create negative emotions, frustration.
also: GPs from two of the focus groups experienced their current workload as sustainable, despite in-				
Lite tveksam till hälsoutfallet här (kanske oxå "exponeringen"). Healthwas measured on a 12-item Salutogenic HealthIndicator Scale (SHIS) that assesses positive health and indi-vidual experiences of physical, mental and social well-being and resources rather than illness.29Dimensions were perceivedstress, energy, physical function, state of morale, psy-chosomatic function, expression of feelings, cognitive ability,social capacity and self-realization	Risk	[illegible]	SHIS	p<0,001
Jag tycker det är tveksamt om denna ska inkluderas. Är verkligen stress related to information system				
Work ethic feasibility = being able to work according to high ethical standards.Work ethic fea-sibility,				
caseload midwifery = a model of care focusing on continuity, ensuring that childbearing women recei				
Ska vi ha med lön som rotorsak till "hälsa"?				
ORsFinns oxå personal burnout och client burn-out som utfall	Risk	3-skift	Work burnout	1,5 (0,6-3,9)
Quick returns were defined aschangeovers between shifts in a rotating schedule that permitted<11 h	Riskfaktor	Schemaläggning night shifts	Sickness absense	Night shifts were not associated wit sickness absence the following month
accreditation: non-monetary incentive in the form of mandatory accreditation. The accreditation pro				
Lean maturity: according to some, Lean needs to be adopted systemwide, involve all Lean principles	frisk	lean (maturity)	thriving	Increased Lean maturity was associated with increased staff thriving, mediated by increased job resou
kan inte hitta separat presenterade resultat för de nordiska länderna men ändå känns detta relevant				
Internal market-oriented culture (IMOC) refers to nursing professionals' perception of managers' nor				
The results of this large cohort of female nurses aged >44 years showed no association between shift	Risk	Skiftarbete	Parkinsons	- Dag = referens- Kväll 0,86 (0,55-1,34)- Natt 1,26 (0,79-2,02)- Roterande 0,83 (0,56-1,21)
	Riskfaktor	Organisational resources	Technostress	Digital literacy, poor user influence and poor redistribution of work and ICT systems were associated
Based on 2 weeks sleep and work diaries				
Caseload midwifery is the only model focusing on continuity of care where an individual midwife (an				
Hazard ratio, justerade modellenFinns resultat för ytterligare 11 utfall (mortalitet i olika sjukdomar)				
Noterar resultat från första trimestern, finns även för andra och tredje		Finns ytterligare 12 variabler som beskriver olika typer av förhållanden - ska vi tabellera allt det?		
	frisk/risk	unit level outsourcing	short-term (<=8 days) and long-term (>= 9 days) sickness absence	the likelihood of both short- and long-term sickness absence dropped in the quarter prior to the chan
	Riskfaktor	Using a mobile version of electronic health records	IT-stress	Nurses who used the mobile version of their EHR had higher levels of stress related to information sy
telomere shortening is associated with increased breast cancer risk in workers with long periods of co				
The risk of IHD was increased among employees who the preceding year had permanent night shifts	Risk	Skiftarbete	hjärtsjukdom	- Dag = referens- Dag och kväll 1,11 (0,79-1,55)- Dag/kväll/natt 0,93 (0,58-1,49)- Ständig natt 1,61 (1,06-2,43)
	Riskfaktor	Quick returns	Cerebrovascular disease (CeVD) and stroke	We observed an excess risk of CeVD (N=223) among employees with >30 quick returns (<28 hours) fr
	friskfaktor	opportunities to look out through windows during a workday	wellbeing and comfort	Having opportunities to look out through windows under a workday reduced the feeling of being trap
	Riskfaktor	Antal patientförflyttningar per pass	Ryggskada	Sign samband mellan antalet patientförflyttningar och förekomst av ryggskada (p<0.001) med OR 3,5
Task delegation is defined as an intentional transfer of clinical tasks from the GP to another health				
är sicknes presenteism ett hälsoutfall?				
Ingår dessa i Vilén et al 2022?				
EHR: Electronic health records"Stress means a situation when a person feels tense, restless, nervous oranxious or is unable to sleep at night because his or her mind is troubled all the time. Do you feel thatkind of stress these days?				
Organizational support is measured by items: Encouragement of ethical activity, Provision of informat				
	risk	absent, distant managers; sufficient personnel; inadequate instructions; counterproductive routines	anxiety, dissatisfaction	when organizational changes were detrimental, workload was overwhelming, shiftwork was excessiv
	risk/frisk	temporary contracts	short-term sickness absence (<= 8 days). Register data.	Temporary contracts moderated the relationship between unit-level downsizing in the next quarter a
	Riskfaktor	Night shift	Occupational accidents	ight shifts were associated with nurses involuntarily dozing off at work (IRR=1.015; 95% CI=1.013–1.0
	Friskfaktor	Ledarskap	Sjukfrånvaro	Balancing high organisational demands through insubordination
regressionsanalyser. Ej justerade. Finns tabell med samband mellan krav och resurser och burn-out o	Risk	Number 24-hour on-call shifts permonth	Work-related Burnout	p=0.026
	risk	environmental hazards	injuries, accidents, musculoskeletal pain	Challenging outdoor conditions and weather=risk for falls etc.Unhygienic Environments inside= head

2 Modifierbar faktor i arbetsmiljön (krav/resurs)	2 Kommentar	3 Risk eller friskfaktor	3 Modifierbar organisatorisk struktur	3 Hälsoutfall
	Restructured ICUs = hospitals merged their general and medical ICUs			
work-life balance		friskfaktor	professional development	job satisfaction and commitment
		Risk	>4 consecutive night shifts	
		Risk	Skiftarbete	fatigue during work
Sufficient resources and the opportunity to work together on right tasks		Friskfaktor	Physicians and registered nurses share unit office, natural meeting place	Motivation
				Short 24-h sleep
		Risk	Långa arbetsskift (≥12 h)	Occupational injuries (excluding violence from patients)
	usingparticipatoryworkingtimeschedulingsoftwarehadlittleeffectonbothobjectivelymeasuredworking			
		Riskfaktor	Schemaförändring - night shift	Shift work disease
counting the minutes while visiting the patients, not being able to do more, do better.				
		Risk	Ständig natt	Work burnout
	Night shifts were defined as≥3 hours of work performed between 23.00 and 05.00 hours			
job resources: influence, Possibilities for development, Quality of leadership, Social support from coll	Thriving was assessed on the basis of participants' experience of their sense of learning and vitality			
	ratio, justerade modellen, Analys AFinns mer resultat ...			
		Friskfaktor	Organisational resources	Technostress
	unit level outsourcing: at least 90% of the employees in one unit stop working in the organization: e.g	frisk/risk	unit level downsizing	short-term sickness absence (<= 8 days).
		Riskfaktor	Number of IT-system used	Self-reported stress
	Hazard ratios, justeradeFinns ett antal ytterligare variabler och resultat för atrial fibrillation (AF)... ...			
	Svårt veta vad vi ska räkna som rotorsaker. Det finns oxå resutlat ; The experience of using EHRs, tech			
		Riskfaktor	Lång arbetstid	Cerebrovascular disease (CeVD) and stroke
having windows in the workplace		riskfaktor	surgical techniques/procedures where everything is seen on screens and requires darkness in the ope	stress, strain on eyesight, headaches, "going into hibernation", decreased ability to perform
	Lite osäker på om denna är en faktor som vi vill/bör ha med			
work overload				
Exceeding the financial constraintsIgnoring high demands for productivityNeglecting administrative demands				
	regressionsanalyser. Ej justerade			
physical workload, awkward postures. , Pressure to be effective and perform tasks quickly, Multitaski		risk	staffing problems	dizziness, nausea, blood pressure problems, seizures and palpitations, headaches, tension and strain i

3 Resultat	3 Modifierbar faktor i arbetsmiljön (krav/resurs)	3 Kommentar	4 Risk eller friskfaktor	4 Modifierbar organisatorisk struktur	4 Hälsoutfall
learning new things, having development discussions with a nurse manager, facilities and time to reac		inkluderar som org. faktor pga definitionen. Struktur för prof. develop.	friskfaktor	recognition from senior management	job satisfaction and commitment
- Fatigue during work 1,05 (0,95-1,13)- Fatigue during free days 1,00 (0,93-1,10)- Svårt att somna 0,95 (0,86-1,05)			Risk	Short shift intervals	
There were no significant differences between theworking time regimes in fatigue during work (mean					
	Sufficient resources and the opportunity to work together on right tasks		Friskfaktor	A permissive atmosphere and the opportunity to learn from failures and successes	Motivation
0,91 (0,79-1,04)					Long 24-h sleep
Working-hour characteristics associated with the length of working hours showed minor associations					
1,23 (1,06-1,42)			Risk	Mer än 5 morgonskift under föregående 7 dagar	Occupational injuries (excluding violence from patients)
changing the work schedule from day work to night work from baseline to follow-up was the stronges			Riskfaktor	Shemaförändring - QR	Shift work disease
1,7 (0,6-6,2)			Risk	Work in more than one area	Work burnout
Access to IT support and assistance were associated with increased technostress					
Experiencing unit-level downsizing was associated with lowered odds of short-term sickness absence					
Those who had 3 or more systems indaily use were 1.23 times more likely to have a high level ofstres			Riskfaktor	Ease of use and technical quality of the IT-system	Self-reported stress
We did not observe an increased risk of CeVD or stroke among employees who often (>10 times per y					
To work in darkness was perceived as stressful on the body and required mental preparedness before	dark workenvironment		riskfaktor	limited access to daylight	fatigue, stress
Staffing challenges creates more pressure and stress on the remaining workers who perform extra tas	time pressure and stress	staffing problems often a consequence of HCWs on sick leave.			

4 Resultat	4 Modifierbar faktor i arbetsmiljön (krav/resurs)	4 Kommentar	5 Risk eller friskfaktor	5 Modifierbar organisatorisk struktur	5 Hälsoutfall	5 Resultat	5 Modifierbar faktor i arbetsmiljön (krav/resurs)	5 Kommentar	6 Risk eller friskfaktor
attention, feedback and thanks concerning one's performance. from patients, team members, nurse			riskfaktor	performance targets related to rewards	stress	rewards also caused feelings of envy and stress			
- Fatigue during work 1,42 (1,19-1,72)- Fatigue during free days 1,25 (1,03-1,49)- Svårt att somna 1,38 (1,13-1,64)									
	Possibility to work independently		Friskfaktor	Possible and comfortable with proposing new own suggestions	Motivation		Possibility to work independently		Friskfaktor
5,87 (1,94-17,8)				Skiftarbete med nattskift	Fatigue during work	1,02 (0,93-1,12)			
1,12 (1,07-1,18)			Risk	Mer än 3 kvälls-skift under föregående 7 dagar	Occupational injuries (excluding violence from patients)	1,15 (1,07-1,23)			
Increase in numbers of QRs worked last year (>10 increase) was associated with an increased develop									
(0,7-1,9)		Exkludera denna?	Risk	Nyligen upplevt omorganisation	Work burnout	1,8 (1,2-2,9)			
Higher levels of ease of use and technical quality wereassociated with lower likelihood of stress.									
Limited access to daylight contributed to fatigue and led to an internal stress.	windowless work environment								

6 Modifierbar organisatorisk struktur	6 Hälsoutfall	6 Resultat	6 Modifierbar faktor i arbetsmiljön (krav/resurs)	6 Kommentar	7 Risk eller friskfaktor	7 Modifierbar organisatorisk struktur	7 Hälsoutfall	7 Resultat	7 Modifierbar faktor i arbetsmiljön (krav/resurs)	7 Kommentar	8 Risk eller friskfaktor
Physicians’clear medical goals for patients	Motivation		Being able to work toward goals; registered nurses’own goals and themedicals goals		Friskfaktor	The daily activity whiteboard shows the goals, what needs to be done each day	Motivation		Being able to work toward goals; registered nurses’own goals and themedicals goals		
	Fatigue during free days	1,38 (1,17-1,63)					Short 24-h sleep	0,92 (0,83-1,03)			

8 Modifierbar organisatorisk struktur	8 Hälsoutfall	8 Resultat	8 Modifierbar faktor i arbetsmiljön (krav/resurs)	8 Kommentar
	Long 24-h sleep	8,04 (2,88-22,5)		
