# Supplementary material for: Organisational-level risk and health-promoting factors within the healthcare sector—a systematic search and review
Source: Front Med (Lausanne). 2025 Jan 17;11:1509023. doi: 10.3389/fmed.2024.1509023 (PMC11783186; doi:10.3389/fmed.2024.1509023)
Supplement: Supplementary file 5 [file Data_Sheet_4.PDF]

Authors	Published Year	Title	Journal	Volume	Issue	Notes
Niinihuhta, M.; Terkamo-Moi	2022	A comprehensive evaluation of factors affecting nurse leaders' work-related well-being	Leadersh. in Health Serv.	35	3	Exclusion reason: Wrong comparator
Andersen, L. P.; Hogh, A.; Bi	2018	Work-related threats and violence in human service sectors: The importance of the psycho-social wo	Work	59	1	Exclusion reason: Wrong comparator
Lexén, A.; Kåhlin, I.; Erlands	2020	Occupational health among swedish occupational therapists: A cross-sectional study	Int. J. Environ. Res. Public Health	17	10	Exclusion reason: Wrong outcomes
Johannessen, D. A.; Nordfjær	2021	Work-related satisfaction among clinicians working at inpatient treatment facilities for substance use	Int. J. Environ. Res. Public Health	18	14	Exclusion reason: Wrong comparator
Mahmood, J. I.; Grotmol, K.	2019	Life satisfaction in Norwegian medical doctors: A 15-year longitudinal study of work-related predicto	BMC Health Serv. Res.	19	1	Exclusion reason: Wrong comparator
Ezzatvar, Y.; Calatayud, J.; Ar	2020	Professional experience, work setting, work posture and workload influence the risk for musculoskel	Int. Arch. Occup. Environ. Health	93	2	Exclusion reason: Wrong population
Bjorkman, A.; Engstrom, M.;	2017	Identified obstacles and prerequisites in telenurses' work environment - a modified Delphi study	BMC Health Serv. Res.	17	1	Exclusion reason: Wrong outcomes
Andersen, L. P.; Elklit, A.; Pil	2021	Work-related violence and organizational commitment among health care workers: does supervisor's	Int. Arch. Occup. Environ. Health	94	7	Exclusion reason: Wrong comparator
Bazazan, A.; Dianat, I.; Bahra	2019	Association of musculoskeletal disorders and workload with work schedule and job satisfaction amor	Int. Emerg. Nurs.	44		Exclusion reason: Wrong population
Hvidtfeldt, U. A.; Bjorner, J.	2017	Cohort Profile: The Well-being in HospitAL Employees (WHALE) study	Int. J. Epidemiol.	46	6	Exclusion reason: Wrong study design
Hadžibajramović, E.; Ahlborg	2019	Concurrent and lagged effects of psychosocial job stressors on symptoms of burnout	Int. Arch. Occup. Environ. Health	92	7	Exclusion reason: Wrong comparator
Marklund, S.; Huang, K.; Zoh	2021	Dentists working conditions–factors associated with perceived workload	Acta Odontol. Scand.	79	4	Exclusion reason: Wrong outcomes
Kirchhoff, J. W.; Marks, A.; H	2021	The impact of information and communication technology on doctors' and registered nurses' workin	J. Multidiscip.Healthc.	14		Exclusion reason: Wrong outcomes
Strandell, R.	2020	Care workers under pressure – A comparison of the work situation in Swedish home care 2005 and 2	Health Soc. Care Community	28	1	Exclusion reason: Wrong study design
Helgesson, M.; Marklund, S.;	2021	Favorable Working Conditions Related to Health Behavior Among Nurses and Care Assistants in Sw	Front. Public Health	9		Exclusion reason: Wrong outcomes
Lindberg, J.; Holmström, P.;	2020	A national perspective about the current work situation at modern radiotherapy departments	Clin. Transl. Radiat. Oncol.	24		Exclusion reason: Wrong outcomes
Lindmark, U.; Wagman, P.; W	2018	Workplace health in dental care – a salutogenic approach	Int. J. Dent. Hyg.	16	1	Exclusion reason: Wrong comparator
Ejlertsson, L.; Heijbel, B.; An	2021	Strengthened workplace relationships facilitate recovery at work – qualitative experiences of an interv	BMC Fam. Pract.	22	1	Exclusion reason: Wrong study design
Cabezas-García, H. R.; Torres	2018	Prevalence of work-related musculoskeletal disorders in professionals of the rehabilitation services an	Fisioterapia	40	3	Exclusion reason: Wrong population
Gustafsson, K.; Marklund, S.;	2020	Presenteeism, psychosocial working conditions and work ability among care workers—a cross-sectio	Int. J. Environ. Res. Public Health	17	7	Exclusion reason: Wrong outcomes
Potrebny, T.; Igland, J.; Espel	2022	Individual and organizational features of a favorable work environment in nursing homes: a cross-sec	BMC Health Serv. Res.	22	1	Exclusion reason: Wrong outcomes
Nilsen, P.; Fernemark, H.; Sei	2021	Working conditions in primary care: a qualitative interview study with physicians in Sweden informed	BMC Fam. Pract.	22	1	Exclusion reason: Wrong outcomes
Bachmann, L.; Michaelsen, R.	2019	Professional vulnerability in mental healthcare contexts: A focus group study of milieu-therapists' ex	Nurs. Open	6	3	Exclusion reason: Wrong outcomes
Vinstrup, J.; Jakobsen, M. D.;	2020	Perceived Stress and Low-Back Pain Among Healthcare Workers: A Multi-Center Prospective Coho	Front. Public Health	8		Exclusion reason: Wrong comparator
Williamsson, A.; Dellve, L.; K	2019	"Nurses' use of visual management in hospitals—A longitudinal, quantitative study on its implication	J. Adv. Nurs.	75	4	Exclusion reason: Wrong outcomes
Wallin, S.; Fjellman-Wiklund,	2022	Work motivation and occupational self-efficacy belief to continue working among ageing home care	BMC Nurs.	21	1	Exclusion reason: Wrong comparator
Leineweber, C.; Marklund, S.;	2020	Work environment risk factors for the duration of all cause and diagnose-specific sickness absence a	Occup. Environ. Med.	77	11	Exclusion reason: Wrong comparator
Van Den Berg, J.; Bäck, F.; H	2017	Transition to a New Neonatal Intensive Care Unit: Positive Effects on Staff Working Environment a	J. Perinat. Neonatal Nurs.	31	1	Exclusion reason: Wrong outcomes
Kaiser, S.; Patras, J.; Adolfsen	2020	Using the Job Demands–Resources Model to Evaluate Work-Related Outcomes Among Norwegian	SAGE Open	10	3	Exclusion reason: Wrong comparator
Marklund, S.; Gustafsson, K.;	2019	Working conditions and compensated sickness absence among nurses and care assistants in Sweden	BMJ Open	9	11	Exclusion reason: Wrong outcomes
da Silva, S. M.; Braga, N. T.; S	2020	Musculoskeletal disorders and actions to reduce the occurrence in nursing workers	Rev. Enferm.	28		Exclusion reason: Wrong population
Helgesson, M.; Marklund, S.;	2020	Interaction effects of physical and psychosocial working conditions on risk for sickness absence: A p	Int. J. Environ. Res. Public Health	17	20	Exclusion reason: Wrong outcomes
Figueiredo, L. C.; Gratão, A.	2021	Musculoskeletal symptoms in formal and informal caregivers of elderly people	Rev Bras Enferm	75	2	Exclusion reason: Wrong population
Anskär, E.; Falk, M.; Sverker,	2022	'But there are so many referrals which are totally … only generating work and irritation': a qualitative	Scand. J. Prim. Health Care			Exclusion reason: Wrong outcomes
Areskoug Josefsson, K.; Avby	2018	Workers' experiences of healthy work environment indicators at well-functioning primary care units i	Scand. J. Prim. Health Care	36	4	Exclusion reason: Wrong comparator
Hislop, J.; Hensman, C.; Isaks	2022	How Does Robot-Assisted Laparoscopic Surgery Impact Pain and Burnout Among Minimally Invasi	Lect. Notes Comput. Sci.	13320 LNCS		Exclusion reason: Wrong publication type
Golay, D.; Sving, C.; Cajande	2022	An Emotion-driven Approach to Hospital Physicians' Work-Related User Experience				Exclusion reason: Wrong publication type
Khamaj, A. M.; Ali, A. M.; Al	2022	Investigating factors affecting musculoskeletal disorders: Predictive models for identifying caregivers	Work	72	4	Exclusion reason: Wrong population
Cargnin, Z. A.; Schneider, D.	2019	Non-specific low back pain and its relation to the nursing work process	Rev. Lat. Am. Enferm.	27		Exclusion reason: Wrong population
Gustafsson, K.; Marklund, S.;	2019	Physical work environment factors affecting risk for disability pension due to mental or musculoskel	BMJ Open	9	10	Exclusion reason: Wrong comparator
Bjaalid, G.; Olsen, E.; Melber	2020	Institutional stress and job performance among hospital employees	Int. J. Organ. Anal.	28	2	Exclusion reason: Wrong outcomes
Lunde, L. K.; Koch, M.; Knar	2017	Associations of objectively measured sitting and standing with low-back pain intensity: A 6-month fo	Scand. J. Work Environ. Health	43	3	Exclusion reason: Wrong comparator
Andersen, L. L.; Villadsen, E.	2020	Influence of physical and psychosocial working conditions for the risk of disability pension among h	Scand. J. Public Health	48	4	Exclusion reason: Wrong comparator
Håkansson, C.; Lexén, A.	2021	The combination of psychosocial working conditions, occupational balance and sociodemographic c	BMC Health Serv. Res.	21	1	Exclusion reason: Wrong outcomes
Anskär, E.; Lindberg, M.; Fal	2019	Legitimacy of work tasks, psychosocial work environment, and time utilization among primary care s	Scand. J. Prim. Health Care	37	4	Exclusion reason: Wrong outcomes
Lundgren, D.; Ernsth Bravell,	2020	The Association Between Psychosocial Work Environment and Satisfaction With Old Age Care Am	J. Appl. Gerontol.	39	7	Exclusion reason: Wrong outcomes
Reijula, J.; Ruohomäki, V.	2018	Perception of hospital environment before and after relocation	Facilities	36	5-6	Exclusion reason: Wrong outcomes

Jeong, C. H.; Yazdanyar, N.	2020	Noise level measured in danish dental clinics				Exclusion reason: Wrong publication type
Khansa, I.; Khansa, L.; Westv	2018	Work-Related Musculoskeletal Injuries in Plastic Surgeons in the United States, Canada, and Norway	Plast. Reconstr. Surg.	141	1	Exclusion reason: Wrong population
Gadolin, C.; Skyvell Nilsson,	2021	Preconditions for nurses' perceived organizational support in healthcare: a qualitative explorative stuc	J. Health Organ. Manage.	35	9	Exclusion reason: Wrong outcomes
Andersen, L. H.; Christensen,	2022	Taking Prior Sick Leave Patterns Into Account When Estimating Health Consequences of Violence c	Work. Health Safety	70	11	Exclusion reason: Wrong population
Muthukrishnan, R.; Maqbool	2021	Ergonomic risk factors and risk exposure level of nursing tasks: association with work-related muscul	Europ. J. Physiother.	23	4	Exclusion reason: Wrong population
Januario, L. B.; Karstad, K.; R	2019	Association between psychosocial working conditions and perceived physical exertion among elderca	Int. J. Environ. Res. Public Health	16	19	Exclusion reason: Wrong outcomes
Gluschkoff, K.; Hakanen, J. J.	2022	The relative importance of work-related psychosocial factors in physician burnout	Occup. Med.	72	1	Exclusion reason: Wrong comparator
Backman, A.; Lindkvist, M.; L	2022	Longitudinal changes in nursing home leadership, direct care staff job strain and social support in Sw	Int. J. Older People Nurs.			Exclusion reason: Wrong outcomes
Stolt, M.; Miikkola, M.; Suhor	2018	Nurses' Perceptions of Their Foot Health: Implications for Occupational Health Care	Work. Health Safety	66	3	Exclusion reason: Wrong outcomes
Marklund, S.; Mienna, C. S.; V	2020	Work ability and productivity among dentists: associations with musculoskeletal pain, stress, and sleep	Int. Arch. Occup. Environ. Health	93	2	Exclusion reason: Wrong comparator
Milton, J.; Erichsen Andersso	2022	Healthcare professionals' perceptions of interprofessional teamwork in the emergency department: a	Scand. J. Trauma Resusc. Emerg. Med.	30	1	Exclusion reason: Wrong outcomes
Xu, H. G.; Johnston, A. N. B.	2019	Stressors and coping strategies of emergency department nurses and doctors: A cross-sectional study	Australas. Emerg. Care	22	3	Exclusion reason: Wrong population
Furunes, T.; Kaltveit, A.; Ake	2018	Health-promoting leadership: A qualitative study from experienced nurses' perspective	J. Clin. Nurs.	27	23-24	Exclusion reason: Wrong outcomes
Vainiomäki, S.; Aalto, A. M.;	2017	Better usability and technical stability could lead to better work-related well-being among physicians	Appl. Clin. Informatics	8	4	Exclusion reason: Wrong outcomes
Gustavsson, M. E.; Juth, N.;	2022	Dealing with difficult choices: a qualitative study of experiences and consequences of moral challenge	Confl. Health	16	1	Exclusion reason: Wrong outcomes
Torp, S.; Bergheim, L. T. J.	2022	Working environment, work engagement and mental health problems among occupational and physi	Scand. J. Occup. Ther.			Exclusion reason: Wrong comparator
Jutengren, G.; Jaldestad, E.; D	2020	The potential importance of social capital and job crafting for work engagement and job satisfaction	Int. J. Environ. Res. Public Health	17	12	Exclusion reason: Wrong outcomes
Burr, H.; Pohrt, A.; Rugulies,	2017	Does age modify the association between physical work demands and deterioration of self-rated gene	Scand. J. Work Environ. Health	43	3	Exclusion reason: Wrong population
Lukasse, M.; Henriksen, L.	2019	Norwegian midwives' perceptions of their practice environment: A mixed methods study	Nurs. Open	6	4	Exclusion reason: Wrong outcomes
Mathisen, J.; Nguyen, T. L.; Je	2022	Impact of hypothetical improvements in the psychosocial work environment on sickness absence rat	Eur J Public Health	32	5	Exclusion reason: Wrong outcomes
Wells, A. C.; Kjellman, M.; Ha	2019	Operating hurts: a study of EAES surgeons	Surg. Endosc.	33	3	Exclusion reason: Wrong population
Maneschiöld, P. O.; Lucaci-M	2021	Nursing assistant's perceptions of the good work environment in municipal elderly care in Sweden –	J. Health Organ. Manage.	35	9	Exclusion reason: Wrong outcomes
Chan, K. A. C.; Molina, J. A.;	2018	Assessment of postural analysis in a dialysis clinic	Adv. Intell. Sys. Comput.	590		Exclusion reason: Wrong publication type
Mauno, S.; Ruokolainen, M.;	2017	Does recovery buffer against emotional labor in terms of motivational outcomes at work? Analyzing	Appl. Nurs. Res.	36		Exclusion reason: Wrong outcomes
Ree, E.	2020	What is the role of transformational leadership, work environment and patient safety culture for pers	Nurs. Open	7	6	Exclusion reason: Wrong outcomes
Berthelsen, H.; Owen, M.; We	2021	Does workplace social capital predict care quality through job satisfaction and stress at the clinic? A p	BMC Public Health	21	1	Exclusion reason: Wrong outcomes
Bry, A.; Wigert, H.	2022	Organizational climate and interpersonal interactions among registered nurses in a neonatal intensive	J. Nurs. Manage.			Exclusion reason: Wrong outcomes
Roelen, C. A. M.; van Hoffen	2018	Psychosocial work environment and mental health-related long-term sickness absence among nurses	Int. Arch. Occup. Environ. Health	91	2	Exclusion reason: Wrong outcomes
Derks, M. T. H.; Mishra, A. K	2018	Understanding thermal comfort perception of nurses in a hospital ward work environment	Build. Environ.	140		Exclusion reason: Wrong population
Arakelian, E.; Rudolfsson, G.;	2019	I Stay—Swedish Specialist Nurses in the Perioperative Context and Their Reasons to Stay at Their W	J. Perianesth. Nurs.	34	3	Exclusion reason: Wrong outcomes
Hildingsson, I.; Karlström, A.	2020	A continuity of care project with two on-call schedules: Findings from a rural area in Sweden	Sex. Reprod. Healthc.	26		Exclusion reason: Wrong population
Jakobsen, M. D.; Sundstrup, I	2017	Psychosocial benefits of workplace physical exercise: Cluster randomized controlled trial	BMC Public Health	17	1	Exclusion reason: Wrong study design
Hølge-Hazelton, B.; Berthelse	2021	Why nurses stay in departments with low turnover: A constructivist approach	Nordic J. Nurs. Res.	41	3	Exclusion reason: Wrong outcomes
Hage, T. W.; Isaksson Rø, K.;	2021	Burnout among staff on specialized eating disorder units in Norway	J. Eating Disord.	9	1	Exclusion reason: Wrong outcomes
Pihl-Thingvad, J.; Brandt, L. I	2018	Consistent Use of Assistive Devices for Patient Transfer Is Associated With Less Patient-Initiated Vi	Work. Health Safety	66	9	Exclusion reason: Wrong outcomes
Thapa, D. R.; Stengård, J.; Ek	2022	Job demands, job resources, and health outcomes among nursing professionals in private and public	BMC Nurs.	21	1	Exclusion reason: Wrong outcomes
Van Diepen, C.; Fors, A.; Ekr	2022	Associations between person-centred care and job strain, stress of conscience, and intent to leave am	J. Clin. Nurs.	31	5-6	Exclusion reason: Wrong outcomes
Debesay, J.; Arora, S.; Fougne	2022	Organisational culture and ethnic diversity in nursing homes: a qualitative study of healthcare workers	BMC Health Serv. Res.	22	1	Exclusion reason: Wrong comparator
Krupic, F.; Sköldenberg, O.; S	2018	Nurses' Experience of Patient Care in Multibed Hospital Rooms: Results From In-Depth Interviews	J. Perianesth. Nurs.	33	1	Exclusion reason: Wrong outcomes
Pedersen, L. M.; Jakobsen, A.	2023	Positive association between social capital and the quality of health care service: A cross-sectional stu	Int. J. Nurs. Stud.	137		Exclusion reason: Wrong outcomes
Sjöberg, A.; Pettersson-Ström	2020	The burden of high workload on the health-related quality of life among home care workers in North	Int. Arch. Occup. Environ. Health	93	6	Exclusion reason: Wrong comparator
Serafin, L.; Bjerså, K.; Dobos	2019	Nurse job satisfaction at a surgical ward - a comparative study between Sweden and Poland	Med Pr	70	2	Exclusion reason: Wrong population
Hörberg, A.; Jirwe, M.; Kalén	2017	We need support! A Delphi study about desirable support during the first year in the emergency med	Scand. J. Trauma Resusc. Emerg. Med.	25	1	Exclusion reason: Wrong outcomes
Carstensen, K.; Jensen, E. K.;	2020	Implementation of integrated operating rooms: How much time is saved and how do medical staff ex	BMJ Open	10	7	Exclusion reason: Wrong outcomes
Eriksson, A.; Vulkan, P.; Dell	2022	A Case Study of Critical Reasons Behind Hospital Nurses Turnover Due to Challenges Across Syster	J. Multidiscip.Healthc.	15		Exclusion reason: Wrong outcomes
Mikkelsen, A.; Olsen, E.	2019	The influence of change-oriented leadership on work performance and job satisfaction in hospitals –	Leadersh. in Health Serv.	32	1	Exclusion reason: Wrong comparator
Aronsson, G.; Marklund, S.; I	2021	The changing nature of work – Job strain, job support and sickness absence among care workers and	SSM Popul. Health	15		Exclusion reason: Wrong outcomes
Hörberg, A.; Kalén, S.; Jirwe,	2018	Treat me nice! -a cross-sectional study examining support during the first year in the emergency medi	Scand. J. Trauma Resusc. Emerg. Med.	26	1	Exclusion reason: Wrong outcomes
Selberg, R.; Sandberg, M.; Mu	2022	Contradictions in Care: Ward Nurses' Experiences of Work and Management in the Swedish Public S	NORA Nordi. J. Fem. Gender Res.	30	2	Exclusion reason: Wrong outcomes
Le Floch, B.; Bastiaens, H.; Le	2019	Which positive factors give general practitioners job satisfaction and make general practice a rewardir	BMC Fam. Pract.	20	1	Exclusion reason: Wrong population
Wentz, K.; Gyllensten, K.; Slu	2020	Need for recovery in relation to effort from work and health in four occupations	Int. Arch. Occup. Environ. Health	93	2	Exclusion reason: Wrong outcomes

Rodríguez-Socarrás, M.; Vasq	2018	"Burnout syndrome": Stress, burnout and depression in Urology	Arch. Esp. Urol.	71	1	Exclusion reason: Wrong language
Schiller, H.; Lekander, M.; Ra	2017	The impact of reduced worktime on sleep and perceived stress – A group randomized intervention st	Scand. J. Work Environ. Health	43	2	Exclusion reason: Wrong population
Mikkola, L.; Suutala, E.; Parvi	2018	Social support in the workplace for physicians in specialization training	Med. Educ. Online	23	1	Exclusion reason: Wrong outcomes
Török, E.; Clark, A. J.; Jensen	2018	Work-unit social capital and long-term sickness absence: A prospective cohort study of 32 053 hospit	Occup. Environ. Med.	75	9	Exclusion reason: Wrong comparator
Jakobsen, M. D.; Vinstrup, J.;	2022	Factors associated with high physical exertion during healthcare work: Cross-sectional study among h	Work	71	4	Exclusion reason: Wrong outcomes
Sousa-Ribeiro, M.; Lindfors, I	2022	Sustainable Working Life in Intensive Care: A Qualitative Study of Older Nurses	Int. J. Environ. Res. Public Health	19	10	Exclusion reason: Wrong outcomes
Arvidsson, I.; Gremark Simor	2020	The impact of occupational and personal factors on musculoskeletal pain - A cohort study of female	BMC Musculoskelet. Disord.	21	1	Exclusion reason: Wrong comparator
Sandberg, L.; Borell, L.; Edva	2018	Job strain: A cross-sectional survey of dementia care specialists and other staff in Swedish home care	J. Multidiscip.Healthc.	11		Exclusion reason: Wrong outcomes
Ericsson, C. R.; Lindström, V	2022	Paramedics' perceptions of job demands and resources in Finnish emergency medical services: a qual	BMC Health Serv. Res.	22	1	Exclusion reason: Wrong outcomes
Roczniewska, M.; Richter, A.;	2020	Predicting sustainable employability in swedish healthcare: The complexity of social job resources	Int. J. Environ. Res. Public Health	17	4	Exclusion reason: Wrong comparator
Török, E.; Rod, N. H.; Ersbø	2020	Can work-unit social capital buffer the association between workplace violence and long-term sicknes	Int. Arch. Occup. Environ. Health	93	3	Exclusion reason: Wrong comparator
Virtanen, M.; Myllyntausta, S.	2021	Shift work, work time control, and informal caregiving as risk factors for sleep disturbances in an age	Scand. J. Work Environ. Health	47	3	Exclusion reason: Wrong population
Larsson, A.; Westerberg, M.; I	2018	Teamwork and safety climate in homecare: A mixed method study	Int. J. Environ. Res. Public Health	15	11	Exclusion reason: Wrong outcomes
Andersson, I.; Eklund, A. J.; N	2022	Prevalence, type, and reasons for missed nursing care in municipality health care in Sweden – A cross	BMC Nurs.	21	1	Exclusion reason: Wrong outcomes
Wålinder, R.; Runeson-Brobe	2018	A supportive climate and low strain promote well-being and sustainable working life in the operation	Upsala J. Med. Sci.	123	3	Exclusion reason: Wrong outcomes
Pennbrant, S.; Dåderman, A.	2021	Job demands, work engagement and job turnover intentions among registered nurses: Explained by v	Work	68	4	Exclusion reason: Wrong outcomes
Dalager, T.; Søgaard, K.; Boyl	2019	Surgery Is Physically Demanding and Associated With Multisite Musculoskeletal Pain: A Cross-Sectic	J. Surg. Res.	240		Exclusion reason: Wrong comparator
Berthelsen, H.; Conway, P. M	2018	Is organizational justice climate at the workplace associated with individual-level quality of care and o	Int. Arch. Occup. Environ. Health	91	2	Exclusion reason: Wrong outcomes
Hovlin, L.; Hallgren, J.; Dahl	2022	The role of the home health care physician in mobile integrated care: a qualitative phenomenograpic :	BMC Geriatr.	22	1	Exclusion reason: Wrong outcomes
Harrison, J.	2019	Organisational factors: impacting on health for ambulance personnel	Int. J. Emerg. Serv.	8	2	Exclusion reason: Wrong publication type
Jakobsen, M. D.; Sundstrup, I	2017	Factors affecting pain relief in response to physical exercise interventions among healthcare workers	Scand. J. Med. Sci. Sports	27	12	Exclusion reason: Wrong study design
Banerjee, S.; Califano, R.; Cor	2017	Professional burnout in European young oncologists: results of the European Society for Medical Or	Ann. Oncol.	28	7	Exclusion reason: Wrong population
Strid, E. N.; Wåhlin, C.; Ros,	2021	Health care workers' experiences of workplace incidents that posed a risk of patient and worker injur	BMC Health Serv. Res.	21	1	Exclusion reason: Wrong outcomes
Mehta, A. J.; Mathisen, J.; Ngu	2022	Chronic disorders, work-unit leadership quality and long-term sickness absence among 33 025 public	Scand. J. Work Environ. Health	48	7	Exclusion reason: Wrong comparator
Hamnerius, N.; Svedman, C.;	2018	Wet work exposure and hand eczema among healthcare workers: a cross-sectional study	Br. J. Dermatol.	178	2	Exclusion reason: Wrong comparator
Kihlberg, J.; Hansson, B.; Hal	2022	Magnetic resonance imaging incidents are severely underreported: a finding in a multicentre interview	Eur. Radiol.	32	1	Exclusion reason: Wrong outcomes
Van Laethem, M.; Beckers, D	2019	Challenge and hindrance demands in relation to self-reported job performance and the role of restora	J. Occup. Organ. Psychol.	92	2	Exclusion reason: Wrong outcomes
Hansson, M.; Lundgren, I.; H	2021	Professional courage to create a pathway within midwives' fields of work: a grounded theory study	BMC Health Serv. Res.	21	1	Exclusion reason: Wrong outcomes
Allesøe, K.; Holtermann, A.; I	2017	Does influence at work modify the relation between high occupational physical activity and risk of he	Int. Arch. Occup. Environ. Health	90	5	Exclusion reason: Wrong outcomes
Pekurinen, V.; Willman, L.; V	2017	Patient aggression and the wellbeing of nurses: A cross-sectional survey study in psychiatric and non-	Int. J. Environ. Res. Public Health	14	10	Exclusion reason: Wrong comparator
Martinussen, P. E.; Davidsen,	2021	'Professional-supportive' versus 'economic-operational' management: the relationship between leade	BMC Health Serv. Res.	21	1	Exclusion reason: Wrong comparator
Eriksson, A.; Jutengren, G.; D	2021	Job demands and functional resources moderating assistant and Registered Nurses' intention to leave	Nurs. Open	8	2	Exclusion reason: Wrong outcomes
Gold, J. E.; Punnett, L.; Gore	2017	Predictors of low back pain in nursing home workers after implementation of a safe resident handling	Occup. Environ. Med.	74	6	Exclusion reason: Wrong population
Nurmeksela, A.; Mikkonen, S.	2021	Relationships between nurse managers' work activities, nurses' job satisfaction, patient satisfaction, ar	BMC Health Serv. Res.	21	1	Exclusion reason: Wrong outcomes
Hamnerius, N.; Svedman, C.;	2018	Hand eczema and occupational contact allergies in healthcare workers with a focus on rubber additiv	Contact Dermatitis	79	3	Exclusion reason: Wrong outcomes
Silén, M.; Skytt, B.; Engström	2019	Relationships between structural and psychological empowerment, mediated by person-centred proce	Geriatr. Nurs.	40	1	Exclusion reason: Wrong comparator
Aalto-Korte, K.; Koskela, K.;	2021	Allergic contact dermatitis and other occupational skin diseases in health care workers in the Finnish	Contact Dermatitis	84	4	Exclusion reason: Wrong comparator
Olsen, E.; Jensen, M. T.; Bjaa	2019	Job resources and outcomes in the process of bullying: a study in a Norwegian healthcare setting	Increasing Occupational Health and Saf. i			Exclusion reason: Wrong publication type
Rosenberg, M. K.; Bonsaksen	2022	Job Satisfaction Among Psychomotor Physiotherapists in Norway	Inquiry	59		Exclusion reason: Wrong comparator
Vauhkonen, A.; Saaranen, T.;	2021	Work community factors, occupational well-being and work ability in home care: A structural equatio	Nurs. Open	8	6	Exclusion reason: Wrong outcomes
Engström, M.; Högberg, H.; S	2021	Staff working life and older persons' satisfaction with care: A multilevel, correlational design	J. Nurs. Care Qual.	36	1	Exclusion reason: Wrong outcomes
Appel, A. M.; Török, E.; Jens	2020	The longitudinal association between shift work and headache: results from the Danish PRISME coh	Int. Arch. Occup. Environ. Health	93	5	Exclusion reason: Wrong comparator
Jäppinen, K.; Roos, M.; Slater	2022	Connection between nurse managers' stress from workload and overall job stress, job satisfaction anc	Nordic J. Nurs. Res.	42	2	Exclusion reason: Wrong outcomes
Ylitörmänen, T.; Turunen, H.	2019	Good nurse–nurse collaboration implies high job satisfaction: A structural equation modelling appro	Nurs. Open	6	3	Exclusion reason: Wrong outcomes
Waage, S.; Bjorvatn, B.	2017	Health, psychosocial and workplace characteristics may identify nurses and midwives at risk of high a	Evid.- Based Nurs.	20	3	Exclusion reason: Wrong publication type
Wesołowska, K.; Elovainio, M	2020	Nativity status and workplace discrimination in registered nurses: Testing the mediating role of psych	J. Adv. Nurs.	76	7	Exclusion reason: Wrong outcomes
Hansen, M. C. T.; Schmidt, J.	2017	Noise exposure during prehospital emergency physicians work on Mobile Emergency Care Units anc	Scand. J. Trauma Resusc. Emerg. Med.	25	1	Exclusion reason: Wrong outcomes
Setrinen Hansen, N. M.; Mikk	2021	Physicians' experiences working in emergency medicine in a rural area in Northern Sweden: a qualitat	Rural Remote Health	21	3	Exclusion reason: Wrong outcomes
Ericsson, C. R.; Nordquist, H	2021	Finnish paramedics' professional quality of life and associations with assignment experiences and def	BMC Public Health	21	1	Exclusion reason: Wrong outcomes
Gustafsson, N.; Salzmann-Eri	2016	Effect of complex working conditions on nurses who exert coercive measures in forensic psychiatric	J. Psychosocial Nurs. Ment. Health Serv.	54	9	Exclusion reason: Wrong outcomes
Gemark Simonsen, J.; Gard, C	2016	Swedish Sonographers' perceptions of ergonomic problems at work and their suggestions for improv	BMC Musculoskelet. Disord.	17	1	Exclusion reason: Wrong outcomes

Kuokkanen, L.; Leino-Kilpi, I	2016	Newly graduated nurses' empowerment regarding professional competence and other work-related fa	BMC Nurs.	15	1	Exclusion reason: Wrong outcomes
Aagestad, C.; Tyssen, R.; Steru	2016	Do work-related factors contribute to differences in doctor-certified sick leave? A prospective study c	BMC Public Health	16	1	Exclusion reason: Wrong population
Vammen, M. A.; Mikkelsen, S	2016	Emotional demands at work and the risk of clinical depression a longitudinal study in the danish pub	J. Occup. Environ. Med.	58	10	Exclusion reason: Wrong outcomes
Rehnström, K.; Dahlborg-Lyc	2016	Proactive Interventions: An Observational Study at a Swedish Emergency Department	SAGE Open	6	3	Exclusion reason: Wrong outcomes
Genç, A.; Kahraman, T.; Göz	2016	The prevalence differences of musculoskeletal problems and related physical workload among hospit	J. Back Musculoskelet. Rehabil.	29	3	Exclusion reason: Wrong population
Allesøe, K.; Søgaard, K.; Aada	2016	Are hypertensive women at additional risk of ischaemic heart disease from physically demanding wor	Eur. J. Prev. Cardiol.	23	10	Exclusion reason: Wrong comparator
Brännström, K. J.; Holm, L.; I	2016	Occupational stress among Swedish audiologists in clinical practice: Reasons for being stressed	Int. J. Audiol.	55	8	Exclusion reason: Wrong study design
Heikkilä, T. J.; Hyppölä, H.; V	2016	What predicts doctors' satisfaction with their chosen medical specialty? A Finnish national study	BMC Med. Educ.	16	1	Exclusion reason: Wrong comparator
Ree, E.; Wiig, S.	2020	Linking transformational leadership, patient safety culture and work engagement in home care servic	Nurs Open	7	1	Exclusion reason: Wrong comparator
Assander, S.; Bergström, A.; (	2022	Individual and organisational factors in the psychosocial work environment are associated with home	BMC Health Serv Res	22	1	Exclusion reason: Wrong outcomes
Sulander, J.; Sinervo, T.; Elov	2016	Does Organizational Justice Modify the Association Between Job Involvement and Retirement Inten	Res Nurs Health	39	5	Exclusion reason: Wrong outcomes
Håkansson, C.; Lexén, A.	2022	Work conditions as predictors of Swedish occupational therapists' occupational balance	Scand J Occup Ther			Exclusion reason: Wrong outcomes
Nunstedt, H.; Eriksson, M.; C	2020	Salutary factors and hospital work environments: a qualitative descriptive study of nurses in Sweden	BMC Nursing	19	1	Exclusion reason: Wrong outcomes
Mattsson, S.; Gustafsson, M.	2020	Job Satisfaction among Swedish Pharmacists	Pharmacy (Basel)	8	3	Exclusion reason: Wrong comparator
Reknes, I.; Notelaers, G.; Mag	2017	Aggression from Patients or Next of Kin and Exposure to Bullying Behaviors: A Conglomerate Expo	Nurs Res Pract	2017		Exclusion reason: Wrong comparator
Astvik, Wanja; Welander, Jon	2021	A comparative study of how social workers' voice and silence strategies relate to organisational resou	Journal of Social Work	21	2	Exclusion reason: Wrong population
Ventovaara, Päivi; af Sandebe	2022	A cross-sectional survey of moral distress and ethical climate – Situations in paediatric oncology care	Nursing Open	9	4	Exclusion reason: Wrong outcomes
Norrman Harling, Malin; Hög	2020	Breaking the taboo: eight Swedish clinical psychologists' experiences of compassion fatigue	International Journal of Qualitative Studie	15	1	Exclusion reason: Wrong outcomes
Fischer, Shelly A.; Jones, Jacq	2018	Consensus achievement of leadership, organisational and individual factors that influence safety clima	Journal of Nursing Management (John W	26	1	Exclusion reason: Wrong population
Arvidsson, Inger; Gremark Si	2016	Cross-sectional associations between occupational factors and musculoskeletal pain in women teache	BMC Musculoskeletal Disorders	17		Exclusion reason: Wrong population
Hansen, Åse Marie; Brødsgaa	2018	Does Workplace Bullying Affect Long-Term Sickness Absence Among Coworkers?	Journal of Occupational & Environmenta	60	2	Exclusion reason: Wrong comparator
Allerby, Katarina; Goulding, A	2019	F132. PERSON-CENTERED PSYCHOSIS CARE (PCPC) IN AN INPATIENT SETTING: WAF	Schizophrenia Bulletin	45		Exclusion reason: Wrong publication type
Tuononen, Tiina; Lammintak	2017	Factors supporting dentist leaders' retention in leadership	Community Dental Health	34	4	Exclusion reason: Wrong outcomes
Lampinen, Mai-Stiina; Konu,	2018	Factors that foster or prevent sense of belonging among social and health care managers	Leadership in Health Services (1751-1879)	31	4	Exclusion reason: Wrong outcomes
Sørensen, Tanja; Tingleff, Ell	2018	Feeling Safe and Taking on Responsibilities: Newly Graduated Nurses' Perceptions and Evaluations c	Journal of Forensic Nursing	14	3	Exclusion reason: Wrong outcomes
André, Beate; Jacobsen, Frod	2022	How is leadership experienced in joy-of-life-nursing-homes compared to ordinary nursing homes: a c	BMC Nursing	21	1	Exclusion reason: Wrong outcomes
Paunova, Minna; Li-Ying, Jas	2023	Interactive effects of self-concept and social context on perceived cohesion in intensive care nursing	Applied Psychology: An International Rev	72	1	Exclusion reason: Wrong outcomes
Molin, Jenny; Strömbäck, Ma	2021	It's Not Just in the Walls: Patient and Staff Experiences of a New Spatial Design for Psychiatric Inpat	Issues in Mental Health Nursing	42	12	Exclusion reason: Wrong outcomes
Hansson, Malin; Dencker, An	2022	Job satisfaction in midwives and its association with organisational and psychosocial factors at work:	BMC Health Services Research	22	1	Exclusion reason: Wrong study design
Greenslade, Jaimi H.; Wallis, I	2020	Key occupational stressors in the ED: an international comparison	Emergency Medicine Journal	37	2	Exclusion reason: Wrong outcomes
Lundgren, Dan; Ernsth-Bravel	2016	Leadership and the psychosocial work environment in old age care	International Journal of Older People Nu	11	1	Exclusion reason: Wrong outcomes
Arakelian, Erebouni; Rudolfs	2021	Managerial challenges faced by Swedish nurse managers in perioperative settings– a qualitative study	BMC Nursing	20	1	Exclusion reason: Wrong outcomes
Cajander, Åsa; Moll, Jonas; E	2018	Medical Records Online for Patients and Effects on the Work Environment of Nurses	Studies in Health Technology & Informat	247		Exclusion reason: Wrong publication type
Andresen, Ida Hellum; Hanse	2017	Norwegian nurses' quality of life, job satisfaction, as well as intention to change jobs	Nordic Journal of Nursing Research	37	2	Exclusion reason: Wrong outcomes
Niinihuhta, Milja; Terkamo-M	2022	Nurse leaders' work-related well-being—Relationships to a superior's transformational leadership styl	Journal of Nursing Management (John W	30	7	Exclusion reason: Wrong comparator
Stevens, Matthew L.; Karstad,	2022	Nursing Home, Ward and Worker Level Determinants of Perceived Quantitative Work Demands: A	Annals of Work Exposures & Health	66	8	Exclusion reason: Wrong outcomes
Tuvesson, Hanna; Eklund, M	2017	Nursing Staff Stress and Individual Characteristics in Relation to the Ward Atmosphere in Psychiatric	Issues in Mental Health Nursing	38	9	Exclusion reason: Wrong outcomes
Arnetz, Judith E.; Zhdanova,	2016	Patient Involvement: A New Source of Stress in Health Care Work?	Health Communication	31	12	Exclusion reason: Wrong outcomes
TÄHtinen, Katja; Remes, Jou	2020	Perceived indoor air quality and psychosocial work environment in office, school and health care env	International Journal of Occupational Me	33	4	Exclusion reason: Wrong comparator
Heponiemi, Tarja; Hyppönen	2018	Predictors of physicians' stress related to information systems: a nine-year follow-up survey study	BMC Health Services Research	18	1	Exclusion reason: Wrong outcomes
Bonsaksen, Tore; Nerdrum, F	2021	Psychological distress and its associations with psychosocial work environment factors in four profes	Nursing & Health Sciences	23	3	Exclusion reason: Wrong outcomes
Karlsson, Ann-Christin; Guni	2019	Registered nurses' perspectives of work satisfaction, patient safety and intention to stay – A double-e	Journal of Nursing Management (John W	27	7	Exclusion reason: Wrong outcomes
Dean, Erin	2017	Sickness absence halved in trial of six-hour day	Nursing Standard	31	20	Exclusion reason: Wrong publication type
Crilly, Julia; Greenslade, Jaimi	2019	Staff perceptions of the emergency department working environment: An international cross-section	Emergency Medicine Australasia	31	6	Exclusion reason: Wrong population
Öhman, Ann; Keisu, Britt-Ing	2017	Team social cohesion, professionalism, and patient-centeredness: Gendered care work, with special r	BMC Health Services Research	17		Exclusion reason: Wrong outcomes
Sveinsdóttir, Herdís; Blöndal,	2018	The content of nurse unit managers' work: a descriptive study using daily activity diaries	Scandinavian Journal of Caring Sciences	32	2	Exclusion reason: Wrong outcomes
Kaihlanen, Anu-Marja; Gluscl	2021	The information system stress, informatics competence and well-being of newly graduated and exper	BMC Health Services Research	21	1	Exclusion reason: Wrong comparator
Saukkonen, Petra; Elovainio,	2022	The Interplay of Work, Digital Health Usage, and the Perceived Effects of Digitalization on Physiciar	Journal of Medical Internet Research	24	8	Exclusion reason: Wrong outcomes
Allwood, Carl Martin; Geisler	2022	The relationship between personality, work, and personal factors to burnout among clinical psycholo	Counselling Psychology Quarterly	35	2	Exclusion reason: Wrong comparator
Lyyra, Eeva; Roos, Mervi; Suc	2021	The workplace culture in addiction psychiatry in Finland as described by healthcare personnel	Advances in Dual Diagnosis	14	3	Exclusion reason: Wrong outcomes
Anskär, Eva; Lindberg, Malou	2018	Time utilization and perceived psychosocial work environment among staff in Swedish primary care s	BMC Health Services Research	18		Exclusion reason: Wrong outcomes

Lohikoski, K.; Roos, M.; Suor	2019	Workplace culture assessed by radiographers in Finland	Radiography	25	4	Exclusion reason: Wrong study design
Gadolin, Christian; Larsman,	2022	How do healthcare unit managers promote nurses' perceived organizational support, and which work	Scandinavian Journal of Psychology	63	6	Exclusion reason: Wrong outcomes
Eklöf, Britta; Larsson, Hanna	2022	The role of self-reported stressors in recovery from exhaustion disorder: A longitudinal study	BMC Psychiatry	22		Exclusion reason: Wrong outcomes
Frennert, Susanne; Erlingsdót	2022	'it increases my ability to influence my ways of working': A qualitative study on digitally mediated pati	Scandinavian Journal of Caring Sciences			Exclusion reason: Wrong setting
Ineland, Jens; Starke, Mikaela	2022	Factors associated with positive work experience among professionals supporting people with intelle	International Journal of Developmental D	68	4	Exclusion reason: Wrong outcomes
Honkalampi, Kirsi; Kupari, Sa	2022	The association between chronotype and sleep quality among female home care workers performing	Chronobiology International	39	5	Exclusion reason: Wrong outcomes
Larsson, Ing-Marie; Aronssor	2022	Healthcare workers' structured daily reflection on patient safety, workload and work environment in i	Intensive and Critical Care Nursing	68		Exclusion reason: Wrong outcomes
Bunkenborg, Gitte; Barfod O	2022	Balancing responsibilities, rewards and challenges: A qualitative study illuminating the complexity of l	Journal of Clinical Nursing			Exclusion reason: Wrong outcomes
Wadman, Cecilia	2022	Psychosocial work conditions and musculoskeletal complaint: The role of affective stress response as			AAI28426849	Exclusion reason: Wrong publication type
Bujacz, Aleksandra; Rudman,	2021	Psychosocial working conditions of shiftworking nurses: A long-term latent transition analysis	Journal of Nursing Management	29	8	Exclusion reason: Wrong outcomes
Brubakk, Kirsten; Svendsen, I	2021	Hospital work environments affect the patient safety climate: A longitudinal follow-up using a logistic	PLoS ONE	16	10	Exclusion reason: Wrong outcomes
Söderbacka, Tina; Nyholm, L	2021	What is giving vitality to continue at work? A qualitative study of older health professionals' vitality so	Scandinavian Journal of Caring Sciences			Exclusion reason: Wrong comparator
Tangsgaard, Emily Rose	2021	How do public service professionals behave in risky situations? The importance of organizational cul	The American Review of Public Administ	51	7	Exclusion reason: Wrong outcomes
Huhtala, Mari; Geurts, Sabine	2021	Intensified job demands in healthcare and their consequences for employee well-being and patient sa	Journal of Advanced Nursing	77	9	Exclusion reason: Wrong comparator
Pettersson, Cecilia; Nilsson, M	2021	The impact of the physical environment for caregiving in ordinary housing: Experiences of staff in ho	Applied Ergonomics	92		Exclusion reason: Wrong outcomes
Munch, Pernille Kold; Nørreg	2021	Which work environment challenges are top of mind among eldercare workers and how would they s	Applied Ergonomics	90		Exclusion reason: Wrong outcomes
Ejlertsson, Lina; Heijbel, Bod	2020	Is it possible to gain energy at work? A questionnaire study in primary health care	Primary Health Care Research and Develo	21		Exclusion reason: Wrong outcomes
Ahlstedt, Carina; Eriksson Lir	2020	Flourishing at work: Nurses' motivation through daily communication—An ethnographic approach	Nursing & Health Sciences	22	4	Exclusion reason: Wrong outcomes
Kalanlar, Bilge; Kuru Alici, N	2020	The effect of care burden on formal caregiver's quality of work life: A mixed-methods study	Scandinavian Journal of Caring Sciences	34	4	Exclusion reason: Wrong population
Bragadóttir, Helga; Burmeiste	2020	The association of missed nursing care and determinants of satisfaction with current position for dire	Journal of Nursing Management	28	8	Exclusion reason: Wrong population
Jensen, Johan Høy; Flachs, Es	2020	Work-unit social capital and incident purchase of psychotropic medications: A longitudinal cohort-stu	Journal of Affective Disorders	276		Exclusion reason: Wrong outcomes
Karhula, Kati; Wöhrmann, An	2020	Working time dimensions and well-being: A cross-national study of Finnish and German health care	Chronobiology International	37	9-10	Exclusion reason: Wrong comparator
Midjo, Turid; Redzovic, Skend	2020	The complexity of work expectations of staff in supported housing	Social Work in Mental Health	18	5	Exclusion reason: Wrong outcomes
Alenius, Lisa Smeds; Lindqvis	2020	Between a rock and a hard place: Registered nurses' accounts of their work situation in cancer care in	European Journal of Oncology Nursing	47		Exclusion reason: Wrong outcomes
Török, Eszter; Clark, Alice Je	2020	Physical workload, long-term sickness absence, and the role of social capital. Multi-level analysis of a	Scandinavian Journal of Work, Environm	46	4	Exclusion reason: Wrong outcomes
Vanttola, Päivi; Puttonen, San	2020	Prevalence of shift work disorder among hospital personnel: A cross-sectional study using objective v	Journal of Sleep Research	29	3	Exclusion reason: Wrong study design
Ibrahim, Maha E.; Cheval, Bo	2020	Back pain occurrence and treatment-seeking behavior among nurses: The role of work-related emoti	Quality of Life Research: An Internationa	29	5	Exclusion reason: Wrong population
Gustafsson, Klas; Marklund, S	2020	Interaction effects of physical and psychosocial working conditions on the risk of disability pension a	International Journal of Nursing Studies	102		Exclusion reason: Wrong outcomes
Gyllensten, Kristina; Wentz, F	2019	Older assistant nurses' motivation for a full or extended working life	Ageing & Society	39	12	Exclusion reason: Wrong outcomes
Johansen, Ayna B.; Kristianse	2019	Secondary traumatic stress in Norwegian SUD-therapists: Symptoms and related factors	Nordic Studies on Alcohol and Drugs	36	6	Exclusion reason: Wrong outcomes
Pergert, Pernilla; Bartholdson	2019	Moral distress in paediatric oncology: Contributing factors and group differences	Nursing Ethics	26	7-8	Exclusion reason: Wrong outcomes
Hagerman, Heidi; Engström,	2019	How do first-line managers in elderly care experience their work situation from a structural and psych	Journal of Nursing Management	27	6	Exclusion reason: Wrong outcomes
Leineweber, Constanze; Mark	2019	Work-related psychosocial risk factors and risk of disability pension among employees in health and p	International Journal of Nursing Studies	93		Exclusion reason: Wrong comparator
Jakobsen, Markus D.; Aust, B	2019	Participatory organizational intervention for improved use of assistive devices in patient transfer: A si	Scandinavian Journal of Work, Environm	45	2	Exclusion reason: Wrong outcomes
Burmeister, Elizabeth A.; Kal	2019	Determinants of nurse absenteeism and intent to leave: An international study	Journal of Nursing Management	27	1	Exclusion reason: Wrong population
Schön Persson, Sophie; Nilss	2018	Resources for work-related well-being: A qualitative study about healthcare employees' experiences o	Journal of Clinical Nursing	27	23-24	Exclusion reason: Wrong comparator
Wahlberg, Anna Carin; Bjork	2018	Expert in nursing care but sometimes disrespected—Telenurses' reflections on their work environme	Journal of Clinical Nursing	27	21-22	Exclusion reason: Wrong comparator
Ylitörmänen, Tuija; Turunen,	2018	Job satisfaction among registered nurses in two Scandinavian acute care hospitals	Journal of Nursing Management	26	7	Exclusion reason: Wrong outcomes
Aalto, Anna-Mari; Heponiem	2018	Social relationships in physicians' work moderate relationship between workload and wellbeing—9-ye	European Journal of Public Health	28	5	Exclusion reason: Wrong comparator
Røen, Irene; Kirkevold, Øyvir	2018	Person-centered care in Norwegian nursing homes and its relation to organizational factors and staff	International Psychogeriatrics	30	9	Exclusion reason: Wrong outcomes
Gustafsson, Maria; Mattsson,	2018	Pharmacists' satisfaction with their work: Analysis of an alumni survey	Research in Social & Administrative Phar	14	7	Exclusion reason: Wrong outcomes
Hylén, Ulrika; Kjellin, Lars; P	2018	Psychosocial work environment within psychiatric inpatient care in Sweden: Violence, stress, and valu	International Journal of Mental Health Nu	27	3	Exclusion reason: Wrong outcomes
Lepistö, Sari; Alanen, Seija; A	2018	Healthcare professionals' work engagement in Finnish university hospitals	Scandinavian Journal of Caring Sciences	32	2	Exclusion reason: Wrong comparator
Backman, Annica; Sjögren, K	2018	Job strain in nursing homes—Exploring the impact of leadership	Journal of Clinical Nursing	27	7-8	Exclusion reason: Wrong outcomes
Holmberg, Christopher; Caro	2018	Job satisfaction among Swedish mental health nursing personnel: Revisiting the two-factor theory	International Journal of Mental Health Nu	27	2	Exclusion reason: Wrong outcomes
Kirkegaard, Marie Louise; Kir	2018	Occupational safety across jobs and shifts in emergency departments in Denmark	Safety Science	103		Exclusion reason: Wrong outcomes
Keisu, Britt-Inger; Öhman, A	2018	Employee effort—Reward balance and first-level manager transformational leadership within elderly	Scandinavian Journal of Caring Sciences	32	1	Exclusion reason: Wrong outcomes
Rugulies, Reiner; Jakobsen, Lo	2018	Managerial quality and risk of depressive disorders among Danish eldercare workers: A multilevel col	Journal of Occupational and Environmen	60	2	Exclusion reason: Wrong outcomes
Ejlertsson, Lina; Heijbel, Bod	2018	Variation, companionship and manageability important for recovery during working hours: A qualitat	Work: Journal of Prevention, Assessment	61	1	Exclusion reason: Wrong outcomes
Nielsen, Helena B.; Larsen, A	2018	Risk of injury after evening and night work—Findings from the Danish Working Hour Database	Scandinavian Journal of Work, Environm	44	4	Exclusion reason: Wrong comparator
Nourollahi, Maryam; Afshari,	2018	Awkward trunk postures and their relationship with low back pain in hospital nurses	Work: Journal of Prevention, Assessment	59	3	Exclusion reason: Wrong population

Ejlertsson, Lina; Heijbel, Bod	2018	Recovery, work-life balance and work experiences important to self-rated health: A questionnaire stu	Work: Journal of Prevention, Assessment	59	1	Exclusion reason: Wrong outcomes
Yepes-Baldó, Montserrat; Ror	2018	Job crafting, employee well-being, and quality of care	Western Journal of Nursing Research	40	1	Exclusion reason: Wrong outcomes
Billsten, Johan; Fridell, Mats;	2018	Organizational Readiness for Change (ORC) test used in the implementation of assessment instrume	Journal of Substance Abuse Treatment	84		Exclusion reason: Wrong outcomes
Kurjenluoma, K.; Rantanen, A	2017	Workplace culture in psychiatric nursing described by nurses	Scandinavian Journal of Caring Sciences	31	4	Exclusion reason: Wrong comparator
Astala, Lena; Roos, Mervi; Ha	2017	Staff experiences of appreciative management in the institutional care of people with intellectual and	Scandinavian Journal of Caring Sciences	31	4	Exclusion reason: Wrong outcomes
Andreassen, Cecilie S.; Bakker	2017	Working conditions and individual differences are weakly associated with workaholism: A 2-3-year pr	Frontiers in Psychology	8		Exclusion reason: Wrong outcomes
Trudel-Fitzgerald, Claudia; Po	2017	The association of work characteristics with ovarian cancer risk and mortality	Psychosomatic Medicine	79	9	Exclusion reason: Wrong population
Casalicchio, Giuseppe; Lesaff	2017	Nonlinear analysis to detect if excellent nursing work environments have highest well-being	Journal of Nursing Scholarship	49	5	Exclusion reason: Wrong population
Karhula, Kati; Puttonen, Sam	2017	Objective working hour characteristics and work–life conflict among hospital employees in the Finni	Chronobiology International	34	7	Exclusion reason: Wrong outcomes
Hakanen, Jari J.; Seppälä, Piia	2017	High job demands, still engaged and not burned out? The role of job crafting	International Journal of Behavioral Medic	24	4	Exclusion reason: Wrong comparator
Pekurinen, Virve Maaret; Väli	2017	Organizational justice and collaboration among nurses as correlates of violent assaults by patients in	Psychiatric Services	68	5	Exclusion reason: Wrong outcomes
Riisgaard, Helle; Søndergaard	2017	Work motivation, task delegation and job satisfaction of general practice staff: A cross-sectional study	Family Practice	34	2	Exclusion reason: Wrong outcomes
Nesje, Kjersti	2017	Professional commitment: Does it buffer or intensify job demands?	Scandinavian Journal of Psychology	58	2	Exclusion reason: Wrong comparator
Uronen, L.; Heimonen, J.; Pu	2017	Health check documentation of psychosocial factors using the WAI	Occupational Medicine	67	2	Exclusion reason: Wrong population
Olafsen, Anja H.; Niemiec, Cl	2017	On the dark side of work: A longitudinal analysis using self-determination theory	European Journal of Work and Organizat	26	2	Exclusion reason: Wrong comparator
Mahmood, Javed Iqbal; Grotr	2017	Contextual factors and mental distress as possible predictors of hazardous drinking in norwegian me	European Addiction Research	23	1	Exclusion reason: Wrong outcomes
Lantta, Tella; Anttila, Minna;	2016	Violent events, ward climate and ideas for violence prevention among nurses in psychiatric wards: A	International Journal of Mental Health Sy	10		Exclusion reason: Wrong outcomes
Meagher, Gabrielle; Szebehely	2016	How institutions matter for job characteristics, quality and experiences: A comparison of home care	Work, Employment and Society	30	5	Exclusion reason: Wrong population
Jakobsen, Louise M.; Jorgens	2016	Emotion work within eldercare and depressive symptoms: A cross-sectional multi-level study assessi	International Journal of Nursing Studies	62		Exclusion reason: Wrong comparator
Eskola, Suvi; Roos, Mervi; Mc	2016	Workplace culture among operating room nurses	Journal of Nursing Management	24	6	Exclusion reason: Wrong outcomes
Saksvik-Lehouillier, Ingvild; F	2016	Hardiness, psychosocial factors and shift work tolerance among nurses – A 2-year follow-up study	Journal of Advanced Nursing	72	8	Exclusion reason: Wrong outcomes
Päätalo, Kati; Kyngäs, Helvi	2016	Well-being at work: Graduating nursing students' perspective in Finland	Contemporary Nurse	52	5	Exclusion reason: Wrong population
Vedaa, Øystein; Krossbakken	2016	Prospective study of predictors and consequences of insomnia: Personality, lifestyle, mental health, a	Sleep Medicine	20		Exclusion reason: Wrong outcomes
Sveinsdóttir, Herdís; Ragnars	2016	Praise matters: The influence of nurse unit managers' praise on nurses' practice, work environment a	Journal of Advanced Nursing	72	3	Exclusion reason: Wrong comparator
Tuisku, Katinka; Pulkki-Råba	2016	Cultural events provided by employer and occupational wellbeing of employees: A cross-sectional stu	Work: Journal of Prevention, Assessment	55	1	Exclusion reason: Wrong outcomes
Freimann, Tiina; Pääsuke, Ma	2016	Work-related psychosocial factors and mental health problems associated with musculoskeletal pain i	Pain Research & Management	2016		Exclusion reason: Wrong population
Heponiemi, Tarja; Presseau, J	2016	On-call work and physicians' turnover intention: The moderating effect of job strain	Psychology, Health & Medicine	21	1	Exclusion reason: Wrong outcomes
Kinnunen-Amoroso, Maritta;	2016	Work-related stress management between workplace and occupational health care	Work: Journal of Prevention, Assessment	54	3	Exclusion reason: Wrong population
Strömgren, Marcus; Eriksson,	2016	Social capital among healthcare professionals: A prospective study of its importance for job satisfacti	International Journal of Nursing Studies	53		Exclusion reason: Wrong comparator
