# Supplementary material for: Organisational-level risk and health-promoting factors within the healthcare sector—a systematic search and review
Source: Front Med (Lausanne). 2025 Jan 17;11:1509023. doi: 10.3389/fmed.2024.1509023 (PMC11783186; doi:10.3389/fmed.2024.1509023)
Supplement: Supplementary file 6 [file Data_Sheet_5.PDF]

First author, year	SCREENING QUESTIONS		1. QUALITATIVE STUDIES					COMMENTS
	S1. Are there clear research questions?	S2. Do the collected data allow to address the research questions?	1.1. Is the qualitative approach appropriate to answer the research question?	1.2. Are the qualitative data collection methods adequate to address the research question?	1.3. Are the findings adequately derived from the data?	1.4. Is the interpretation of results sufficiently substantiated by data?	1.5. Is there coherence between qualitative data sources, collection, analysis and interpretation?	
Ahlstedt et al 2019	Yes	Yes	Yes	Yes	Yes	Yes	Yes	No information on the use of reporting framework.
Fallman et al 2022	Yes	Yes	Yes	Yes	Yes	Yes	Yes	No information on the use of reporting framework.
Gamskjaer et al 2022	Yes	Yes	Yes	Yes	Yes	Yes	Yes	No information on the use of reporting framework.
Golay et al 2022a	Yes	Yes	Yes	Yes	Yes	Yes	Yes	No information on the use of reporting framework.
Golay et al 2022b	Yes	Yes	Yes	Yes	Yes	Yes	Yes	No information on the use of reporting framework.
Golvani 2021	Yes	Yes	Yes	Yes	Yes	Yes	Yes	
Grønoset Grasmo et al 2021	Yes	Yes	Yes	Yes	Yes	Yes	Yes	

Gyllensten et al 2017	Yes	Yes	Yes	Yes	Yes	Yes	Yes	No information on the use of reporting framework.
Herttuala et al 2020	Yes	Yes	Yes	Yes	Yes	Yes	Yes	No information on the use of reporting framework.
Jepsen et al 2016	Yes	Yes	Yes	Yes	Yes	Yes	Yes	No information on the use of reporting framework.
Kjellström 2017	Yes	Yes	Yes	Yes	Yes	Yes	Yes	No information on the use of reporting framework.
Lee et al 2021	Yes	Yes	Yes	Yes	Yes	Yes	Yes	No information on the use of reporting framework.
Loft et al 2020	Yes	Yes	Yes	Yes	Yes	Yes	Yes	
Nielsen and Jørgensen 2016	Yes	Yes	Yes	Yes	Yes	Yes	Yes	No information on the use of reporting framework.
Ose et al 2019	Yes	Yes	Yes	Yes	Yes	Yes	Yes	No information on the use of reporting framework.
Seitovirta et al 2017	Yes	Yes	Yes	Yes	Yes	Yes	Yes	No information on the use of reporting framework.
Stadin et al 2020	Yes	Yes	Yes	Yes	Yes	Yes	Yes	No information on the use of

								reporting framework.
Svedahl et al 2019	Yes	Yes	Yes	Yes	Yes	Yes	Yes	No information on the use of reporting framework.
Thapa et al 2021	Yes	Yes	Yes	Yes	Yes	Yes	Yes	
Westergren and Lindberg 2022	Yes	Yes	Yes	Yes	Yes	Yes	Yes	No information on the use of reporting framework.

	**SCREENING QUESTIONS**		**3. RANDOMIZED CONTROLLED STUDIES**					
First author, year	S1. Are there clear research questions?	S2. Do the collected data allow to address the research questions?	2.1. Is randomization appropriately performed?	2.2. Are the groups comparable at baseline?	2.3. Are there complete outcome data?	2.4. Are outcome assessors blinded to the intervention provided?	2.5 Did the participants adhere to the assigned intervention?	**COMMENTS**
Jakobsen et al 2018	Yes	Yes	Yes	No	Yes	Yes	Yes	
Pedersen et al 2020	Yes	Yes	Yes	Can't tell	Can't tell	Yes	Can't tell	

	**SCREENING QUESTIONS**		**3. NON-RANDOMIZED STUDIES**					

First author, year	S1. Are there clear research questions?	S2. Do the collected data allow to address the research questions?	3.1. Are the participants representative of the target population?	3.2. Are measurements appropriate regarding both the outcome and intervention (or exposure)?	3.3. Are there complete outcome data?	3.4. Are the confounders accounted for in the design and analysis?	3.5. During the study period, is the intervention administered (or exposure occurred) as intended?	**COMMENTS**
Andersen et al 2019	Yes	Yes	Yes	Yes	Yes	Yes	Yes	
Beltagy et al 2018	Yes	Yes	Yes	Yes	Yes	Yes	Yes	
Bernstrøm and Houkes 2020	Yes	Yes	Yes	Yes	Yes	Yes	Yes	
Bigert et al 2022	Yes	Yes	Yes	Yes	Yes	Yes	Yes	
Blomberg et al 2016	Yes	Yes	Yes	Yes	Yes	No	Yes	
Cheng et al 2021	Yes	Yes	Yes	Yes	Yes	Yes	Yes	
Cohidon et al 2019	Yes	Yes	Yes	Yes	Yes	Yes	Yes	
Dahlgren et al 2021	Yes	Yes	Can't tell	Yes	Yes	Yes	Yes	Unknown response rate
Erdem et al 2017	Yes	Yes	Yes	Can't tell	Yes	Yes	Can't tell	Self-reported retrospective working hours for cases and controls
Fallman et al 2019	Yes	Yes	Yes	Yes	Yes	Yes	Yes	
Grønstad et al 2019	Yes	Yes	Yes	Yes	Yes	Yes	Yes	
Grønstad et al 2020	Yes	Yes	Yes	Yes	Yes	Yes	Yes	
Hammer et al 2019	Yes	Yes	Yes	Yes	Yes	Yes	Yes	

Hansen et al 2016	Yes	Yes	Yes	Yes	Yes	Yes	Can't tell	Exposure registered in 1993 or 1999 only
Henriksen and Lukasse 2016	Yes	Yes	Yes	Yes	Yes	No	Yes	
Heponiemi et al 2017	Yes	Yes	Yes	Yes	Yes	Yes	Yes	
Heponiemi et al 2019	Yes	Yes	Can't tell	Yes	Yes	Yes	Yes	Low response rate
Heponiemi et al 2021	Yes	Yes	Can't tell	Yes	Yes	Yes	Yes	Unknown response rate
Holmberg et al 2016	Yes	Yes	Yes	Yes	Yes	Yes	Yes	
Hult et al 2022	Yes	Yes	Can´t tell	Yes	Yes	Yes	Yes	Low response rate
Härmä et al 2018	Yes	Yes	Yes	Yes	Yes	Yes	Yes	
Härmä et al 2019	Yes	Yes	Yes	Yes	Yes	Yes	Yes	
Härmä et al 2020	Yes	Yes	Yes	Yes	Yes	Yes	Yes	
Jacobsen et al 2022	Yes	Yes	Yes	Yes	Yes	Yes	Yes	
Jensen et al 2018	Yes	Yes	No	Yes	Yes	No	Yes	
Jepsen et al 2017	Yes	Yes	Yes	Yes	Yes	No	Yes	
Johnsen et al 2022	Yes	Yes	Can't tell	Yes	Yes	No	Yes	Low response rate
Jørgensen et al 2017	Yes	Yes	Yes	Yes	Yes	Yes	Can't tell	Exposure registered in 1993, 1999 and/or 2009 only
Jørgensen et al 2020	Yes	Yes	Yes	Yes	Yes	Yes	Can't tell	Exposure registered in 1993, 1999 and/or 2009 only
Jørgensen et al 2021a	Yes	Yes	Yes	Yes	Yes	Yes	Can't tell	Exposure registered in 1993, 1999 and/or 2009 only

Jørgensen et al 2021b	Yes	Yes	Yes	Yes	Yes	Yes	Can't tell	Exposure registered in 1993, 1999 and/or 2009 only
Kader et al 2021	Yes	Yes	Yes	Yes	Yes	Yes	Yes	
Kader et al 2022	Yes	Yes	Yes	Yes	Yes	Yes	Yes	
Kaltenbrunner et al 2019	Yes	Yes	No	Yes	Yes	Yes	Yes	Convenience sampling
Karhula et al 2018	Yes	Yes	Yes	Yes	Yes	Can´t tell	Yes	
Karhula et al 2020	Yes	Yes	Yes	Yes	Yes	Yes	Yes	
Kjørstad et al 2022	Yes	Yes	No	Yes	Yes	No	Yes	Convenience sampling, low response rate
Larsen et al 2020	Yes	Yes	Yes	Yes	Yes	Yes	Can't tell	
Lindegård et al 2016	Yes	Yes	Can't tell	Yes	Yes	No	Yes	
Liss et al 2018	Yes	Yes	Yes	Yes	Yes	Yes	Yes	
Lunde et al 2021	Yes	Yes	No	Yes	Yes	Yes	Yes	
Mauno et al 2016	Yes	Yes	Yes	Yes	Yes	Yes	Yes	
Møller et al 2022	Yes	Yes	Yes	Yes	Yes	No	Yes	
Nielsen et al 2019a	Yes	Yes	Yes	Yes	Yes	Yes	Yes	
Nielsen et al 2019b	Yes	Yes	Yes	Yes	Yes	Yes	Yes	
Olsen et al 2017	Yes	Yes	Yes	Yes	Yes	No	Yes	
Persson et al 2018	Yes	Yes	Yes	Yes	Yes	Yes	Yes	
Poikkeus et al 2020	Yes	Yes	Can't tell	Yes	Yes	No	Yes	
Rantanen et al 2016	Yes	Yes	Yes	Yes	Yes	No	Yes	
Riisgaard et al 2017	Yes	Yes	Yes	Yes	Yes	Yes	Yes	

Ropponen et al 2019	Yes	Yes	Yes	Yes	Yes	Yes	Yes	
Ropponen et al 2020	Yes	Yes	Yes	Yes	Yes	Yes	Yes	
Ropponen et al 2022	Yes	Yes	Yes	Yes	Yes	Yes	Yes	
Ropponen et al 2023	Yes	Yes	Yes	Yes	Yes	Yes	Yes	
Rosenström et al 2021	Yes	Yes	Yes	Yes	Yes	Yes	Yes	
Ruotsalainen et al 2023	Yes	Yes	Can't tell	Yes	Yes	Yes	Can't tell	
Sigursteinsdóttir et al 2020	Yes	Yes	Yes	Yes	Yes	Yes	Yes	
Slåtten et al 2022	Yes	Yes	No	Yes	Yes	Yes	Yes	Convenice sampling, low response rate
Spännargård et al 2022	Yes	Yes	Can't tell	Yes	Yes	Yes	Yes	Unknown response rate
Thun et al 2018	Yes	Yes	Yes	Yes	Yes	No	Yes	
Vainiomäki et al 2020	Yes	Yes	Yes	Yes	Yes	Yes	Yes	
Vedaa et al 2017a	Yes	Yes	No	Yes	Yes	No	Yes	Convenience sampling
Vedaa et al 2017b	Yes	Yes	Yes	Yes	Yes	Yes	Yes	
Vedaa et al 2019	Yes	Yes	Yes	Yes	Yes	Yes	Yes	
Vedaa et al 2020	Yes	Yes	Yes	Yes	Yes	Yes	Yes	
Vifladt et al 2016	Yes	Yes	Yes	Yes	Yes	Yes	Yes	
Vilén and Putus 2021	Yes	Yes	Can't tell	Yes	Yes	No	Yes	Unknown response rate
Vilén et al 2022	Yes	Yes	Can't tell	Yes	Yes	No	Yes	Unknown response rate
Vinstrup et al 2020	Yes	Yes	Yes	Yes	Yes	Yes	Yes	

Waage et al 2021	Yes	Yes	Yes	Yes	Yes	Yes	Yes	
Westergren et al 2020	Yes	Yes	Yes	Yes	Yes	Yes	Yes	

	**SCREENING QUESTIONS**		**5. MIXED METHODS STUDIES**					**COMMENTS**
First author, year	S1. Are there clear research questions?	S2. Do the collected data allow to address the research questions?	5.1. Is there an adequate rationale for using a mixed methods design to address the research question?	5.2. Are the different components of the study effectively integrated to answer the research question?	5.3. Are the outputs of the integration of qualitative and quantitative components adequately interpreted?	5.4. Are divergences and inconsistencies between quantitative and qualitative results adequately addressed?	5.5. Do the different components of the study adhere to the quality criteria of each tradition of the methods involved?	
Ose et al 2022	Yes	Yes	Yes	Yes	Yes	Yes	No	Low response rate. No information on the use of reporting framework.
Ruotsalainen et al 2020	Yes	Yes	Yes	Yes	Yes	Yes	Can't tell	No information on the use of reporting framework.
